# Delimitation of five astome ciliate species isolated from the digestive tube of three ecologically different groups of lumbricid earthworms, using the internal transcribed spacer region and the hypervariable D1/D2 region of the 28S rRNA gene

**DOI:** 10.1186/s12862-020-1601-2

**Published:** 2020-03-14

**Authors:** Tomáš Obert, Peter Vďačný

**Affiliations:** Department of Zoology, Faculty of Natural Sciences, Comenius University in Bratislava, Bratislava, 842 15 Slovak Republic

**Keywords:** *Anoplophrya*, Astomatia, CBC analysis, ITS2 secondary structure, *Metaradiophrya*, *Subanoplophrya* gen. n.

## Abstract

**Background:**

Various ecological groups of earthworms very likely constitute sharply isolated niches that might permit speciation of their symbiotic ciliates, even though no distinct morphological features appear to be recognizable among ciliates originating from different host groups. The nuclear highly variable ITS1–5.8S-ITS2 region and the hypervariable D1/D2 region of the 28S rRNA gene have proven to be useful tools for the delimitation of species boundaries in closely related free-living ciliate taxa. In the present study, the power of these molecular markers as well as of the secondary structure of the ITS2 molecule were tested for the first time in order to discriminate the species of endosymbiotic ciliates that were isolated from the gastrointestinal tract of three ecologically different groups of lumbricid earthworms.

**Results:**

Nineteen new ITS1–5.8S-ITS2 region and D1/D2-28S rRNA gene sequences were obtained from five astome species (*Anoplophrya lumbrici*, *A. vulgaris*, *Metaradiophrya lumbrici*, *M. varians*, and *Subanoplophrya nodulata* comb. n.), which were living in the digestive tube of three ecological groups of earthworms. Phylogenetic analyses of the rRNA locus and secondary structure analyses of the ITS2 molecule robustly resolved their phylogenetic relationships and supported the distinctness of all five species, although previous multivariate morphometric analyses were not able to separate congeners in the genera *Anoplophrya* and *Metaradiophrya*. The occurrence of all five taxa, as delimited by molecular analyses, was perfectly correlated with the ecological groups of their host earthworms.

**Conclusions:**

The present study indicates that morphology-based taxonomy of astome ciliates needs to be tested in the light of molecular and ecological data as well. The use of morphological identification alone is likely to miss species that are well delimited based on molecular markers and ecological traits and can lead to the underestimation of diversity and overestimation of host range. An integrative approach along with distinctly increased taxon sampling would be helpful to assess the consistency of the eco-evolutionary trend in astome ciliates.

## Background

The association of symbionts with a certain systematic and/or ecological group of host species within the same geographic region could lead to sympatric speciation, since different host groups might constitute sharply isolated niches [1, 2]. Host-driven diversification in ciliates, which is a highly diverse group of eukaryotic microbes classified in the phylum Ciliophora, is still a contentious and insufficiently explored topic [3, 4]. Although nearly one third of described ciliates (~ 2600 species) are symbionts of a huge variety of invertebrates and vertebrates [5], mechanisms that govern their speciation are still poorly understood. Symbiotic ciliates are often thought of as rather promiscuous to their hosts [6, 7], albeit the ciliate-animal symbiotic associations are only seldomly analyzed with the aid of sophisticated molecular taxonomic/systematic methods [8–10]. A part of the “promiscuity problem” might have been caused by a lack of reliable data on the associations of ciliate species with their hosts [11, 12]. These issues can now be independently tested with molecular phylogenetic tools. Recent phylogenetic studies have suggested that some endosymbiotic ciliates cluster according to associations with higher taxa of their hosts. For instance, astome ciliates originating from terrestrial oligochaetes form a clade that is sister to the cluster of astomes from marine polychaetes [11–14]; clevelandellid ciliates, which live in the hindgut of panesthiine cockroaches, form a monophyletic group [4, 15]; and trichostome ciliates exhibit a clustering specific for higher taxa of their vertebrate hosts and individual gastrointestinal compartments [10, 16–18].

Reliable taxonomic data are indispensable in order to assess the level of host specificity of symbiotic ciliates. However, subtle morphological differences and their tiny sizes hinder proper species identification for many ciliates and molecular data are often required to corroborate the identity of the species. This applies also to the species-rich astome genera *Anoplophrya* Stein, 1860 and *Metaradiophrya* Jankowski, 2007 [12]. Both have a difficult taxonomic history due to the paucity of morphologically diagnostic features available for sound species identification as well as due to the lack of information on intraspecific variability and host spectrum [12, 19–23]. The best way to obtain more robust species identification is an integrative approach that combines morphological and molecular data with ecological features [24]. We adopted this strategy in order to examine species boundaries in five endosymbiotic ciliate species that had been isolated from the gastrointestinal tract of lumbricid earthworms in Western Slovakia, Central Europe [12]. Our previous multivariate morphometric analyses revealed an overlap between two *Metaradiophrya* species on one hand and two *Anoplophrya* species on the other one. Moreover, 18S rRNA gene sequences of *Metaradiophrya lumbrici* did not group together in likelihood and Bayesian phylogenetic analyses, which is likely due to the “plesiomorphic/homoplastic trap” [12]. Therefore, in the present study, we have attempted to address the problem of delimitation of these five astome species by using the more variable nuclear internal transcribed spacers of the rRNA genes, along with the first two barcoding domains of the 28S rRNA gene.

## Results

### Characterization of new sequences

In total, 19 new ITS1–5.8S-ITS2 region (referred to as ‘ITS region’ henceforth) and partial 28S rRNA gene (D1/D2) sequences were obtained from *Anoplophrya lumbrici* (Schrank, 1803) Stein, 1860 (2 sequences), *A. vulgaris* de Puytorac, 1954 (5 sequences), *Metaradiophrya lumbrici* (Dujardin, 1841) Jankowski, 2007 (6 sequences), *M. varians* (de Puytorac, 1954) Jankowski, 2007 (4 sequences), and *Subanoplophrya nodulata* (Dujardin, 1841) comb. n. (2 sequences) (for nomenclatural changes, see Discussion and Taxonomic summary). These new sequences were derived from 11 populations isolated from the gastrointestinal tract of lumbricid oligochaetes that belong to three different ecological groups: the endogeic (subsoil-dwelling) *Octolasion tyrtaeum* (Savigny, 1826), the anecic (subsoil- and topsoil-dwelling) *Lumbricus terrestris* Linné, 1758 as well as the epigeic (litter- or surface-dwelling) *Eisenia fetida* (Savigny, 1826) and *Dendrobaena veneta* (Rosa, 1886) (Additional file 1: Table S1).

The length, GC content, and GenBank accession numbers of the new ciliate ITS region-28S rRNA gene sequences are summarized in Table 1. Their total length varies from 1326 to 1343 nt and their GC content ranges from 46.02 to 51.06%. Intraspecies sequence similarity is 100%, except for *S. nodulata* where one polymorphic nucleotide position was detected.

**Table 1:** Characterization of the ITS region and partial 28S rRNA gene sequences newly obtained from astome ciliates isolated from lumbricid oligochaetes

Species	Specimen^a^	Host species	Locality code^b^	Length (nt)	GC (%)	GenBank entry
*Anoplophrya lumbrici*	KR 11 LT	*L. terrestris*	KR	1339	48.24	MN897871
	RZ 6 LT	*L. terrestris*	RZ	1339	48.24	MN897872
*Subanoplophrya nodulata*	PU 29 OT	*O. tyrtaeum*	PU	1330	41.73	MN897873
	PU 30 OT	*O. tyrtaeum*	PU	1330	41.80	MN897874
*Anoplophrya vulgaris*	JA-1 20 EF	*E. fetida*	JA-1	1326	51.06	MN897875
	JA-1 21 EF	*E. fetida*	JA-1	1326	51.06	MN897876
	NG 27 DV	*D. veneta*	NG	1326	51.06	MN897877
	NG 28 DV	*D. veneta*	NG	1326	51.06	MN897878
	BZ 13 EF	*E. fetida*	BZ	1326	51.06	MN897879
*Metaradiophrya lumbrici*	JA-2 25 LT	*L. terrestris*	JA-2	1341	46.83	MN897880
	JA-2 26 LT	*L. terrestris*	JA-2	1341	46.83	MN897881
	KR 8 LT	*L. terrestris*	KR	1341	46.83	MN897882
	KR 10/1 LT	*L. terrestris*	KR	1341	46.83	MN897883
	RZ 4 LT	*L. terrestris*	RZ	1341	46.83	MN897884
	RZ 5 LT	*L. terrestris*	RZ	1341	46.83	MN897885
*Metaradiophrya varians*	BZ 12 EF	*E. fetida*	BZ	1343	46.02	MN897886
	BZ 14 EF	*E. fetida*	BZ	1343	46.02	MN897887
	JA-1 19 EF	*E. fetida*	JA-1	1343	46.02	MN897888
	JA-1 22 EF	*E. fetida*	JA-1	1343	46.02	MN897889

^a^ Specimen code consists of a locality code as specified in Additional file 1: Table S1, an isolate code, and an abbreviation of host species name specified as follows: DV: *Dendrobaena veneta*; EF: *Eisenia fetida*; LT: *Lumbricus terrestris*; and OT: *Octolasion tyrtaeum*

^b^ For locality codes and further details, see Additional file 1: Table S1

### Phylogenetic analyses

**Fig. 1:**
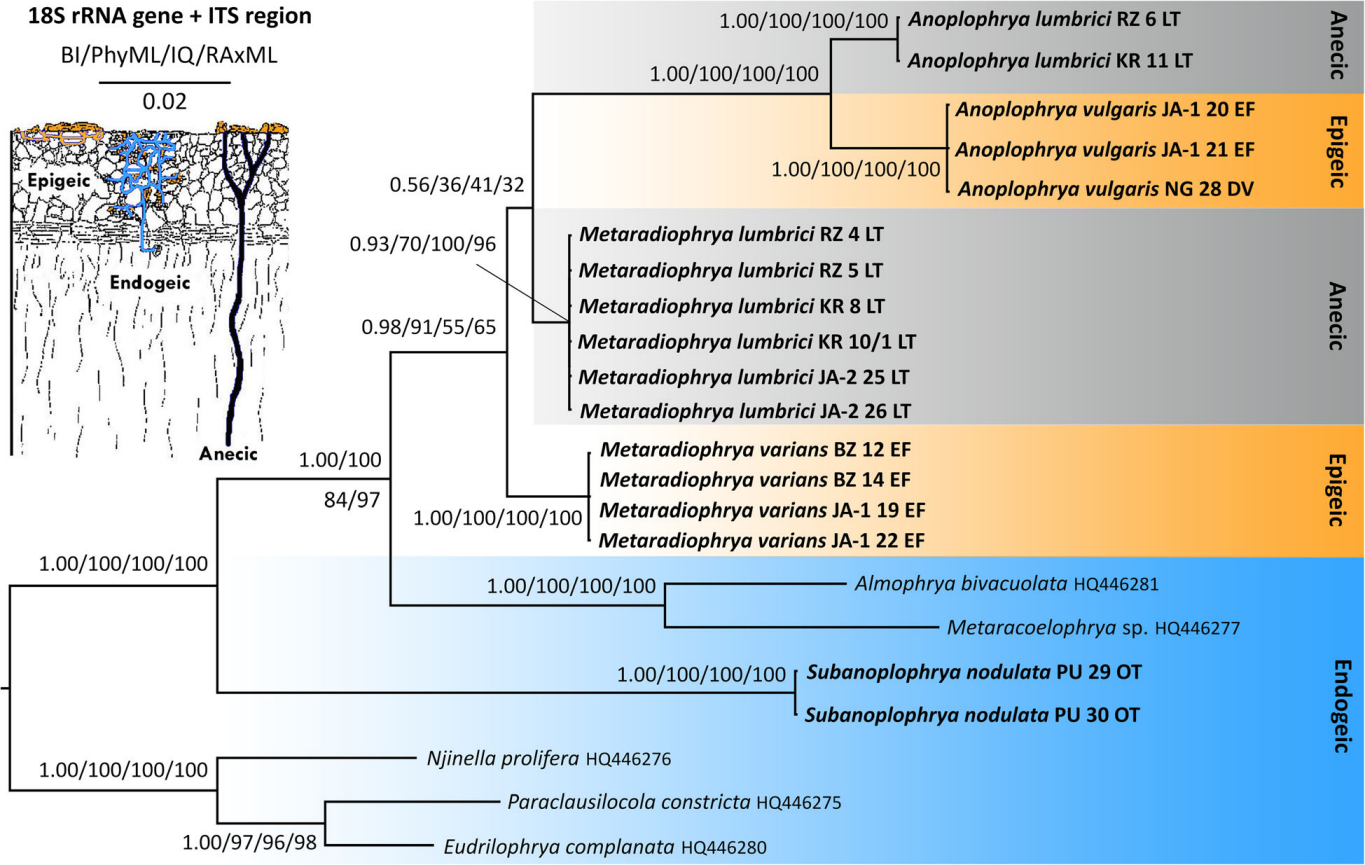
Phylogenetic tree based on the nuclear 18S rRNA gene and the ITS1–5.8S-ITS2 region, showing the phylogenetic positions of astome ciliates isolated from lumbricid earthworms. The tree was rooted according Obert and Vďačný [12]. Posterior probabilities for Bayesian Inference (BI) conducted in MrBayes and bootstrap values for Maximum Likelihood conducted in PhyML, IQTrees, and RAxML were mapped onto the 50%-majority rule Bayesian consensus tree. The phylogenetic tree suggests that the evolution of endosymbiotic astome ciliates has proceeded through specialization to ecological groups of their host earthworms. Sequences in bold face were obtained during this study. For specimen codes and further details, see Table 1. The scale bar denotes two substitutions per one hundred nucleotide positions

**Fig. 2:**
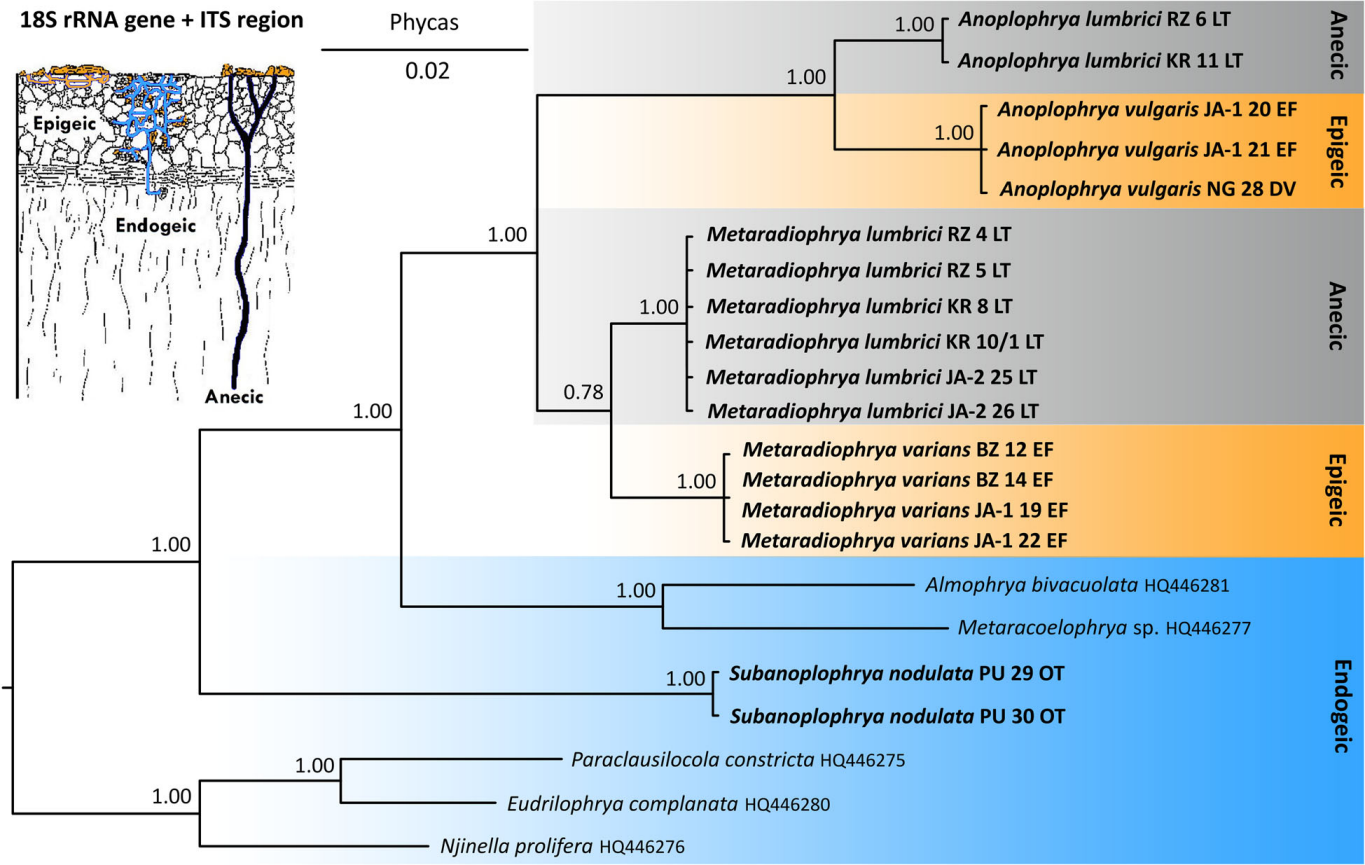
Phylogenetic tree based on the nuclear 18S rRNA gene and the ITS1–5.8S-ITS2 region, showing the phylogenetic positions of astome ciliates isolated from lumbricid earthworms. The tree was rooted according Obert and Vďačný [12]. Posterior probabilities for Bayesian Inference conducted in Phycas were mapped onto the 50%-majority rule consensus tree. Sequences in bold face were obtained during this study. For specimen codes and further details, see Table 1. The scale bar denotes two substitutions per one hundred nucleotide positions

**Fig. 3:**
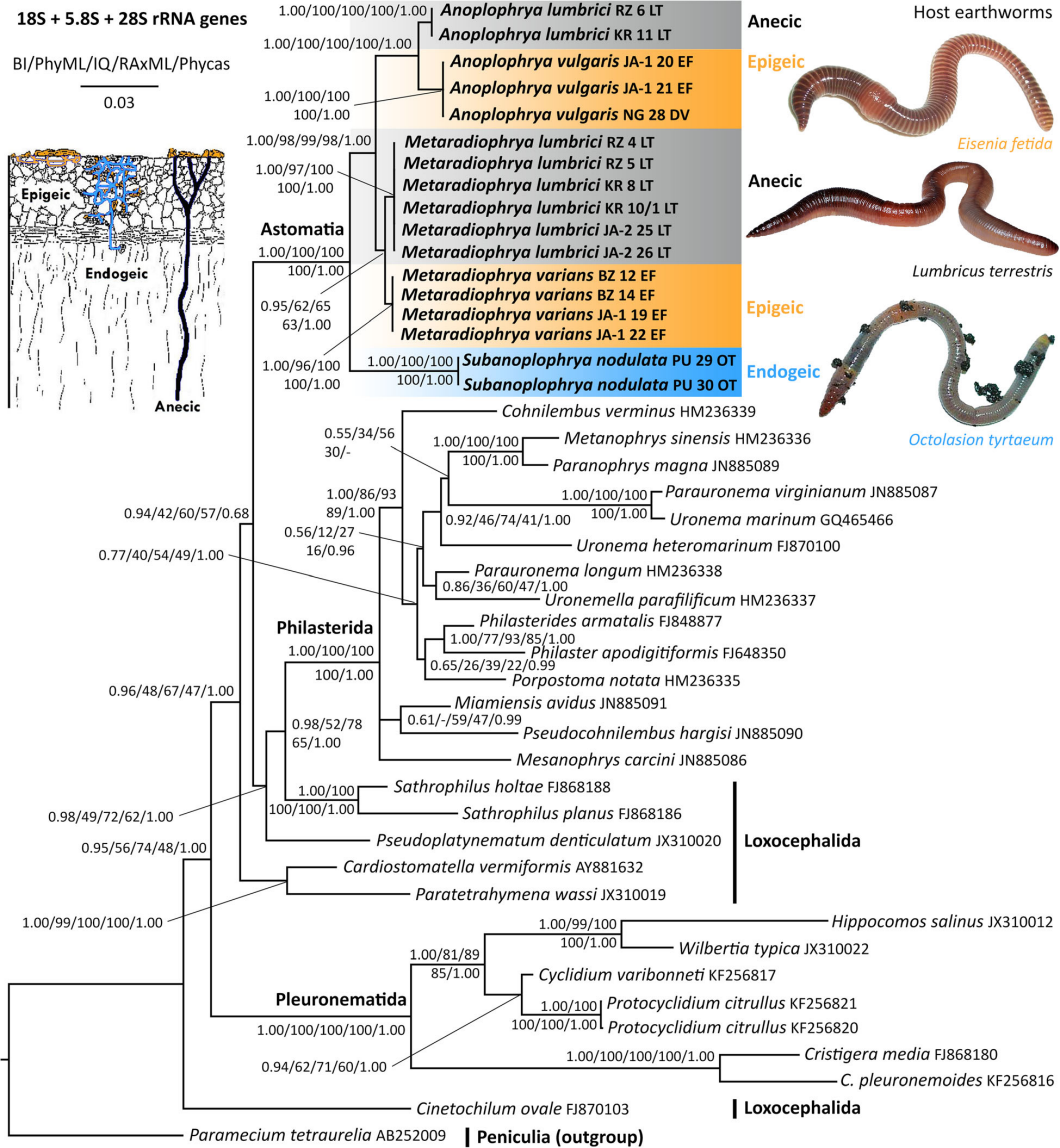
Phylogenetic tree based on the nuclear 18S, 5.8S, and 28S rRNA genes, showing the phylogenetic positions of astome ciliates isolated from lumbricid earthworms. Members of the subclass Scuticociliatia, which is represented here by the orders Philasterida, Pleuronematida, and the polyphyletic Loxocephalida, are the nearest relatives of astome ciliates. The peniculine *Paramecium tetraurelia* was used to a posteriori root the trees. Posterior probabilities for Bayesian inferences conducted in MrBayes (BI) and Phycas as well as bootstrap values for Maximum Likelihood conducted in PhyML, IQTrees, and RAxML were mapped onto the 50%-majority rule Bayesian consensus tree. The phylogenetic tree suggests that the evolution of endosymbiotic astome ciliates has proceeded through specialization to ecological groups of their host earthworms. Sequences in bold face were obtained during this study. For specimen codes and further details, see Table 1. The scale bar denotes three substitutions per one hundred nucleotide positions

Bayesian inferences and maximum likelihood analyses were conducted to determine the phylogenetic position of the new sequences within the subclass Astomatia based on the 18S rRNA gene and the newly obtained ITS region sequences (Figs. 1 and 2). Astomes, which had been isolated from endogeic earthworms, formed a paraphyletic assemblage that encompassed *Subanoplophrya nodulata* and members of the genera *Almophrya* de Puytorac & Dragesco, 1969, *Eudrilophrya* de Puytorac, 1969, *Metaracoelophrya* de Puytorac & Dragesco, 1969, *Njinella* Ngassam, 1983, and *Paraclausilocola* Fokam et al., 2011. Their evolutionary interrelationships might indicate that astomes associated with oligochaetes have ancestrally colonized the gut of endogeic earthworms. Astomes isolated from anecic and epigeic earthworms formed a monophylum that was depicted to be sister of the *Almophrya*–*Metaracoelophrya* clade. *Anoplophrya lumbrici*, which was isolated from anecic earthworms, was classified in a sister position to *A. vulgaris*, which inhabited the gastrointestinal tract of two epigeic earthworm species, with full statistical support in all phylogenetic analyses. However, the phylogenetic position of *M. lumbrici*, which was isolated from anecic earthworms, and of *M. varians*, which was isolated from epigeic earthworms, was left unresolved in the 18S-ITS region trees. Specifically, the clade joining *M. lumbrici* to *A. lumbrici* + *A. vulgaris* received very low support in trees constructed using MrBayes, PhyML, IQTrees, and RAxML (Fig. 1), indicating that there is an alternative relationship. This is very likely a clade of *M. lumbrici* + *M. varians*, as suggested by Bayesian analyses conducted in Phycas (Fig. 2) as well as by the 18S–5.8S-28S rRNA gene tree shown in Fig. 3 (for further details, see below).

28S rRNA gene sequences were not available for the astome ciliates prior to the present study. A comparison of the new ITS region and partial 28S rRNA gene sequences, using the bioinformatics BLASTn tool, revealed that the astome sequences are most similar to those of the subclass Scuticociliatia, or more specifically, to members belonging to its orders Loxocephalida, Philasterida, and Pleuronematida (E-values ranging from 1e–131 to 7e–109 and identity from 84.17 to 87.07%). The concatenated 18S–5.8S-28S rRNA gene trees did not support the monophyletic origin of the three above-mentioned scuticociliate orders, but indicated paraphyly of the subclass Scuticociliatia (Fig. 3). Furthermore, the monophyletic order Philasterida and several members of the non-monophyletic order Loxocephalida formed a variably supported clade together with the astomes (0.96 BI, 48% PhyML, 67% IQTrees, 47% RAxML, 1.00 Phycas). The evolutionary relationships among the astome ciliates corresponded very well to those in the 18S-ITS region trees, i.e., astomes from endogeic earthworms branched off first (represented here by *S. nodulata*) and the astomes from anecic and epigeic earthworms formed strongly statistically supported monophyly. *Anoplophrya lumbrici* specimens from anecic earthworms were sister to *A. vulgaris* individuals from epigeic earthworms and *M. lumbrici* isolates from anecic earthworms were sister to *M. varians* specimens from epigeic earthworms. However, this relationship was not recognized in the tree shown in Fig. 1, as mentioned above.

### Putative secondary structure of ITS2 molecules

The putative secondary structure of ITS2 molecules of the five astome ciliates isolated from lumbricid earthworms were predicted on the Mfold webserver, using the free-energy minimization approach and homology modelling. The length of the ITS2 transcripts varies from 184 nt in *A. vulgaris* to 194 nt in *S. nodulata* and their GC content ranges from 37.63% in *S. nodulata* to 53.80% in *A. vulgaris*. The estimated thermodynamic energy of the putative ITS2 secondary structures is from − 41.10 kcal/mol in *S. nodulata* to − 52.30 kcal/mol in *M. lumbrici* (Table 2).

**Table 2:** Characterization of the ITS2 molecules of astome ciliates

Taxon	Total length	GC cont-ent	Length of helix					Number of unpaired bases in							No. of bulges in helix III	No. of GU pairings	Δ*G* (37 °C, kcal/mol)
			I	E1	II	III	IV	Common loop(s)	Terminal loop of helix II	Terminal loop of helix III	Terminal loop of helix IV	Bulge(s) of helix II	Bulges of helix III	Bulges of helix IV			
*Almophrya bivacuolata*	182	46.15	–	10	26	75	26	45	4	3	11	4	18	1	5	4	−48.10
*Anoplophrya lumbrici*	191	48.17	–	10	26	80	24	51	4	5	11	4	19	1	5	3	−48.30
*Subanoplophrya nodulata*	194	37.63	–	8	28	81	17	60	4	3	11	4	18	–	5	4	−41.10
*Anoplophrya vulgaris*	184	53.80	–	10	24	78	19	53	4	5	11	4	19	–	5	5	−42.80
*Eudrilophrya complanata*	186	43.55	–	6	26	73	19	62	4	5	11	4	26	–	5	3	−32.10
*Metaracoelophrya* sp.	185	36.76	–	10	26	74	17	58	4	3	11	4	21	–	5	3	−35.90
*Metaradiophrya lumbrici*	188	44.68	19	–	24	89	29	27	4	7	4	4	18	7	6	8	−52.30
*Metaradiophrya varians*	193	44.04	25	–	29	89	29	21	5	7	4	4	18	7	6	9	−49.70
*Njinella prolifera*	188	50.53	–	6	26	77	17	62	4	3	11	6	24	–	5	3	−35.70
*Paraclausilocola constricta*	183	47.54	–	6	25	74	19	59	3	4	11	4	20	–	5	3	−37.50
Minimum	182	36.76	19	6	24	73	17	21	3	3	4	4	18	–	5	3	−52.30
Maximum	194	53.80	25	10	29	89	29	62	5	7	11	6	26	–	6	9	−32.10
Arithmetic mean	187.4	45.30	–	–	26.0	79.0	21.6	49.8	–	–	–	–	20.1	–	–	4.5	−42.35
Standard deviation	4.17	5.27	–	–	1.56	5.89	4.93	14.67	–	–	–	–	2.81	–	–	2.22	6.97

**Fig. 4:**
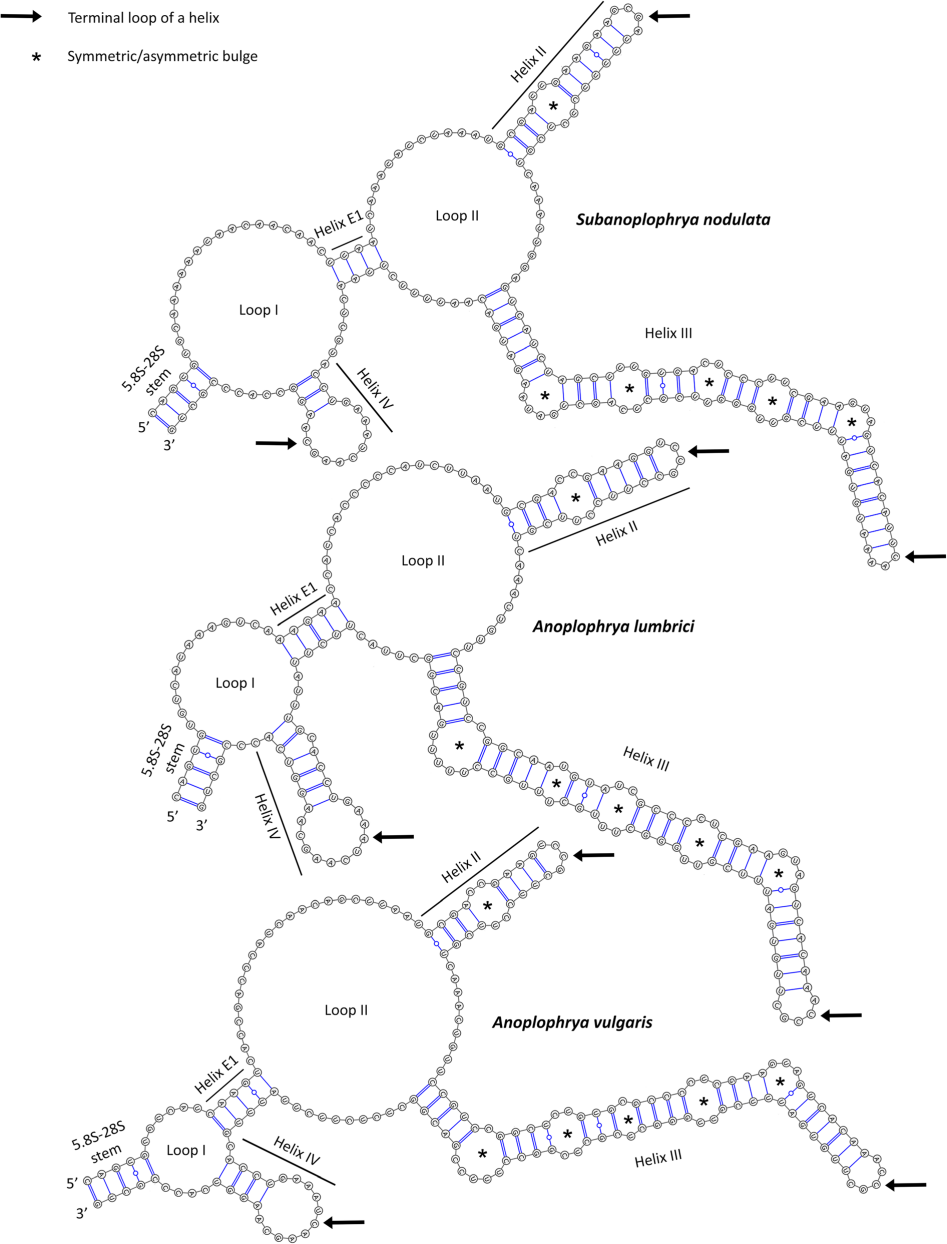
Putative secondary structure of ITS2 molecules of *Subanoplophrya* and two *Anoplophrya* species

**Fig. 5:**
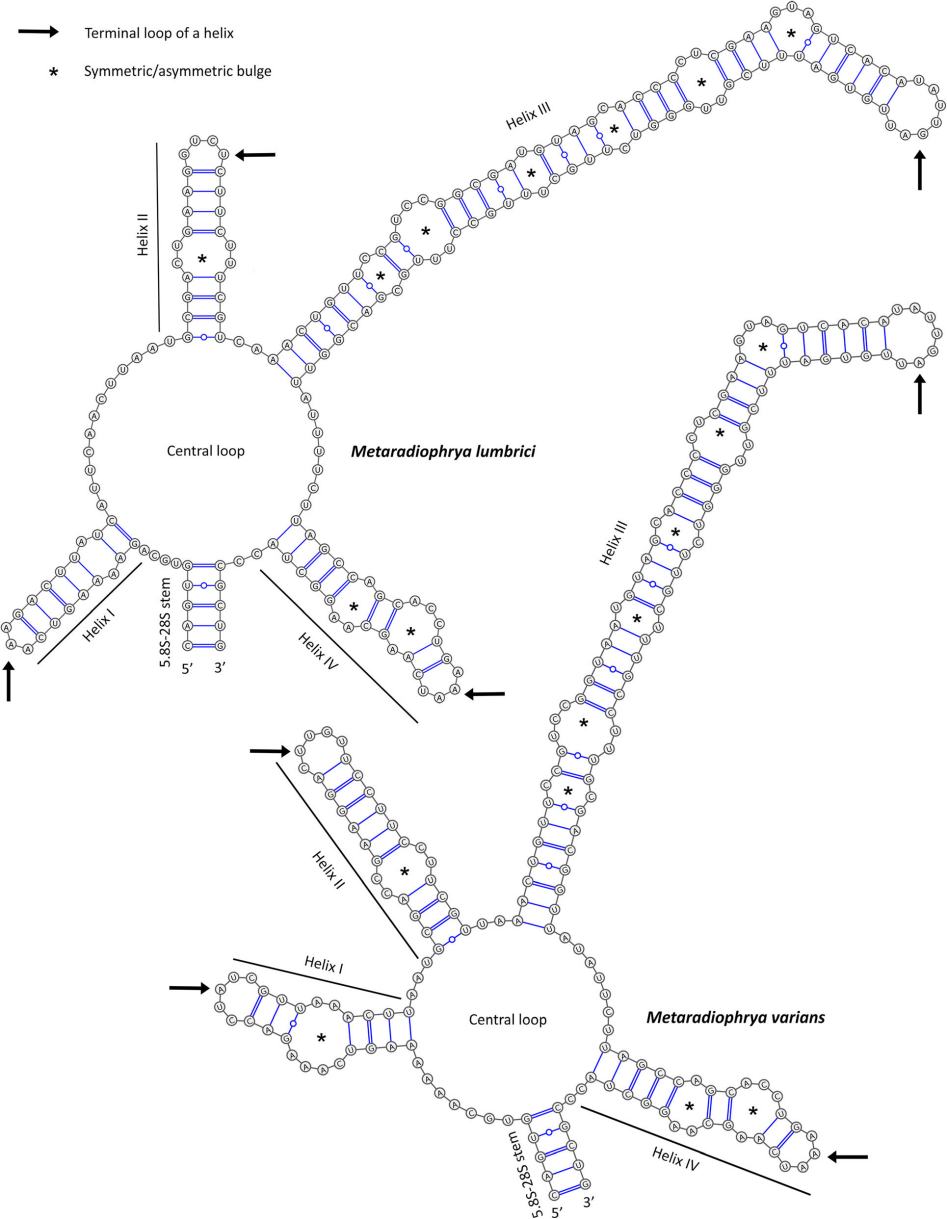
Putative secondary structure of ITS2 molecules of two *Metaradiophrya* species

The folding pattern of the ITS2 molecules differs considerably among *Anoplophrya/Subanoplophrya* and *Metaradiophrya* species (cp. Figs. 4 and 5). *Anoplophrya* and *Subanoplophrya* species shared a hairpin model in which the common loop was started and closed by an extra helix E1 and radiated two helices corresponding to helices II and III of other ciliates (Fig. 4). However, the two *Metaradiophrya* species had a ring model with common loop that radiated four unequally long helices (Fig. 5). The structure of helix I in *Metaradiophrya* species quite varied. Helix I of *M. lumbrici* was 19 nt long and had a single purine-purine mismatch and a terminal loop composed of three nucleotides, while helix I of *M. varians* was 25 nt long and had a conspicuous bulge loop of six unpaired nucleotides and a terminal pentaloop (Fig. 5; Table 2). Helix II was conserved the most among *Anoplophrya*, *Subanoplophrya*, and *Metaradiophrya* species, consisted of 24–29 nt, and exhibited a motif 5′-GCGAYYGAAG vs. YUUCYYUCGU-3′, a single pyrimidine-pyrimidine bulge, and a terminal loop of 4 or 5 nt. Helix III was the longest and most varied among the five taxa. In *Anoplophrya* and *Subanoplophrya* species, it consisted of 79 to 81 nt and invariably had five bulges. The two closely related taxa, *A. lumbrici* and *A. vulgaris*, displayed a motif 5′-CCGUC vs. GACGG-3′ at its base. Helix III was consistently 89 nt long, contained six bulges, and had a motif of 5′-AACUGUU vs. GACGGUU-3′ at its base in the two *Metaradiophrya* species. The position and structure of helix IV was also considerably different among the astome species. In *Anoplophrya* and *Subanoplophrya*, helix IV emerged from a loop situated in the front of the extra helix E1, consisted of 17–24 nt, and had a conserved motif 5′-CCU vs. AGG-3′ at the base of its terminal loop, which invariably contained 11 nt*.* In *Metaradiophrya*, helix IV was situated on the central loop, consistently contained 29 nt and had a motif of 5′-UAGCC vs. GGCUA-3′ at its base and a terminal loop of invariably 4 nt (Figs. 4 and 5; Table 2).

We also predicted the secondary structure of the ITS2 molecules for further five astome ciliate species isolated from the gut of glossoscolecid and megascolecid earthworms, whose ITS region sequences were deposited in the GenBank by Fokam et al. [25]. Their putative secondary structure matched the hairpin model of *Anoplophrya/Subanoplophrya* species in that the common loop was started and closed by an extra helix E1 and radiated only helices II and III (Table 2 and Additional file 2: Figure S1, Additional file 3: Figure S2, Additional file 4: Figure S3, Additional file 5: Figure S4, and Additional file 6: Figure S5).

### Consensus ITS2 secondary structure

**Fig. 6:**
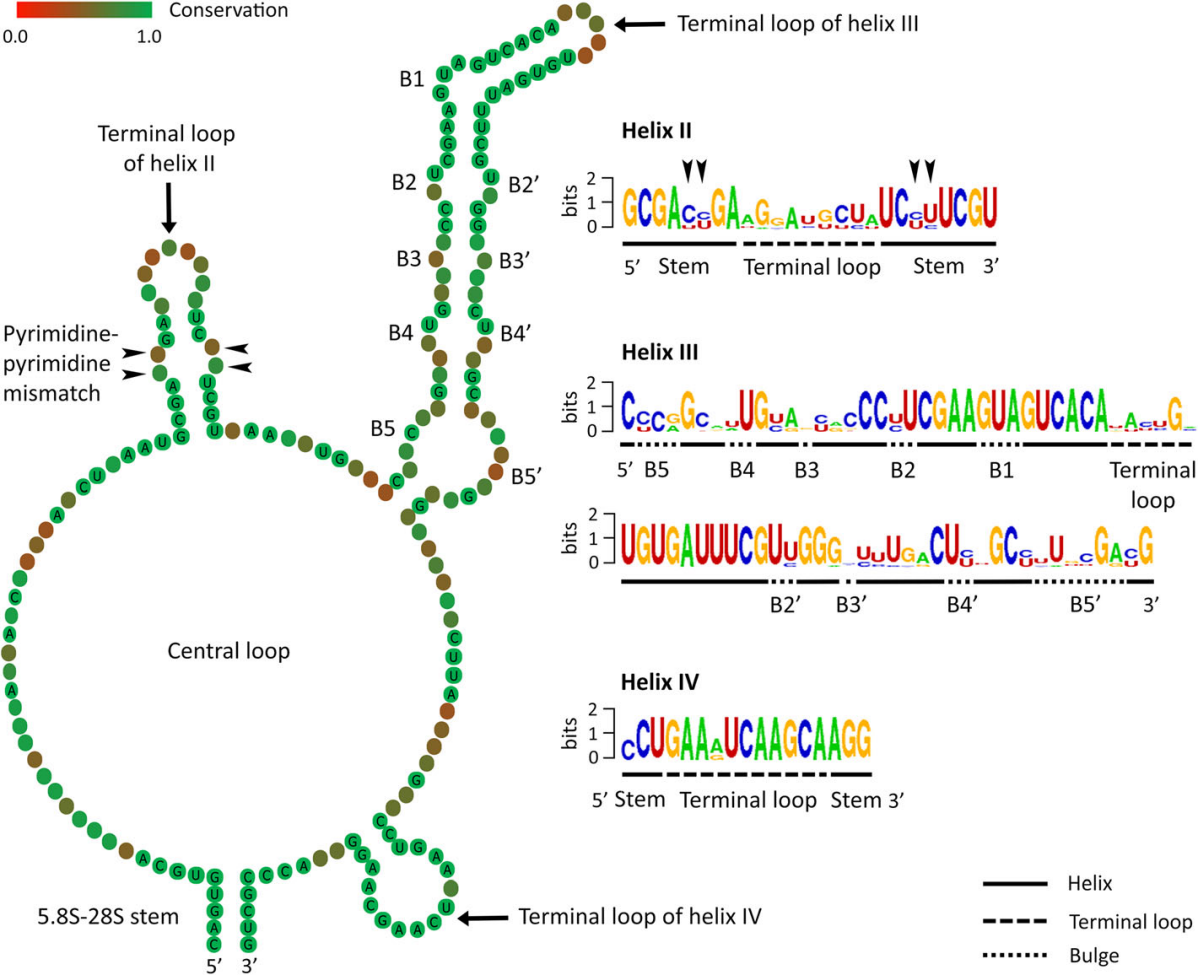
Consensus secondary structure of the astome ITS2 molecule, showing a central loop with three helices corresponding to helices II, III, and IV of other ciliates. Note that helix II has a pyrimidine-pyrimidine mismatch (arrowheads) and helix III bears five bugles (B1–5). Structure logo of the three conserved helices is shown in the right panel. The height of a base is proportional to its frequency in the multiple sequence alignment

The consensus secondary structure of all ten available astome ITS2 molecules was proposed using the 4SALE package. As shown in Fig. 6, the consensus structure consisted of a central loop bearing three helices that correspond to helices II, III, and IV of other ciliates. Helices I and E1 were not introduced into the consensus structure because helix I was present only in the two *Metaradiophrya* species and its structure as well as its nucleotide composition were quite different between the two species (Fig. 5). Although helix E1 was present in all remaining astome species (Fig. 4 and Additional file 2: Figure S1, Additional file 3: Figure S2, Additional file 4: Figure S3, Additional file 5: Figure S4, and Additional file 6: Figure S5), its structure and position were highly varied, thus raising the question of the homology of this extra helix across the astome ciliates studied. Therefore, we can speculate that helix I may not be present in some astomes, and helix E1 may be a variable constituent of their ITS2 molecules.

### Compensatory base changes in ITS2 molecule

**Fig. 7:**
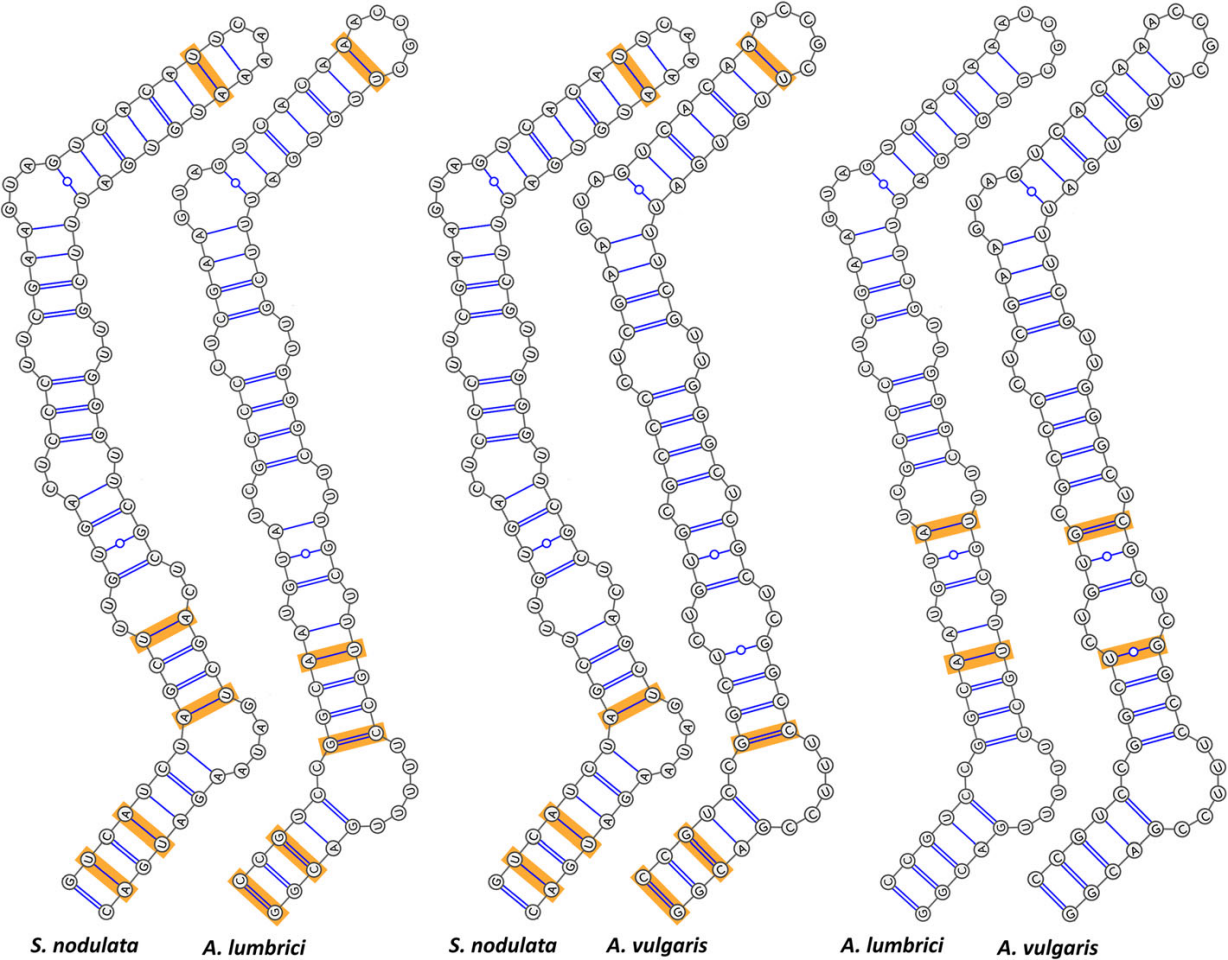
Comparison of helix III of the ITS2 molecule of *Subanoplophrya* and *Anoplophrya* species. CBCs are marked by yellow boxes. There are five CBCs between *S. nodulata* and *A. lumbrici*, four CBCs between *S. nodulata* and *A. vulgaris*, and two CBCs between *A. lumbrici* and *A. vulgaris*

Compensatory base changes (CBCs) are substitutions in two positions that retain pairing. No CBC was detected between the two *Metaradiophrya* species, while multiple CBCs were revealed in helix III of the *Anoplophrya* and *Subanoplophrya* species. More specifically, five CBCs were found between *S. nodulata* and *A. lumbrici*, which involved two changes A = U ↔ U = A and three changes C ≡ G ↔ U = A. Four CBCs separated *S. nodulata* from *A. vulgaris*: a single change A = U ↔ U = A and three changes C ≡ G ↔ U = A. The two closely related *A. lumbrici* and *A. vulgaris* were separated by two CBCs, viz., A = U ↔ U–G and A = U ↔ G ≡ C (Fig. 7).

## Discussion

### Comparison of single gene and multi-gene phylogenies

Although less astome taxa are available for multi-gene phylogenies, the general picture is similar to that obtained in analyses based only on the 18S rRNA gene [11–14, 25]. However, the concatenated datasets provide a better resolution and higher statistical support. Moreover, concatenation of three ribosomal RNA genes helps to overcome the “plesiomorphic/homoplastic trap” that caused inconsistencies in the classification of the two *Metaradiophrya* species in the 18S rRNA gene phylogenies [12]. The problem of the plesiomorphic trap in phylogenetic inferences was introduced by Wägele and Mayer [26]. Although plesiomorphies are homologies, they are old, conserved character states that do not substantiate monophyly of a clade. If old character states are substituted along only some lineages of a clade and/or reversals (“back mutations”) occur along only some branches of that clade, then old common similarities may have the effect of synapomorphies. Such a mosaic-like preservation of plesiomorphies and/or reversals to plesiomorphies might increase the probability of obtaining a false tree. Obert and Vďačný [12] recognized that 19 out of the 25 variable nucleotide positions in the 18S rRNA gene of *M. lumbrici* and *M. varians* are either retained old plesiomorphies or reversals (homoplasies) (see Fig. 16 in [12]). Due to the significantly increased ratio of plesiomorphies (homoplasies) to apomorphies (19:6) in the *Metaradiophrya* sequences, Obert and Vďačný [12] ascribed the topological inconsistencies to the plesiomorphic trap, which causes false paraphyly of *Metaradiophrya*. In the present study, we included further molecular markers and tested the power of three likelihood and two Bayesian approaches in order to overcome the plesiomorphic trap. Monophyly of the genus *Metaradiophrya* and its two subclades was indeed better supported in the concatenated 18S–5.8S-28S rRNA gene dataset than in the 18S rRNA gene and the 18S rRNA gene + ITS region tree (cp. Figs. 1, 2 and 3). The Bayesian approach implemented in Phycas outperformed all other methods used and recognized monophyly of the genus *Metaradiophrya* and its two subclades, not only in the concatenated 18S–5.8S-28S rRNA gene dataset (Fig. 3), but also in the 18S rRNA gene + ITS region tree, though with poor statistical support (Fig. 2). This branching pattern, which is consistent with morphological data, was also revealed in the 18S rRNA gene tree constructed with the distance neighbor-joining algorithm [12]. The log likelihoods of 18S rRNA gene trees where *Metaradiophrya* and its two clusters are/are not monophyletic differ by only 2.6 log units, which is statistically insignificant (Table 6 in [12]). Since the present Phycas analyses exclude the possibility of the “star-tree paradox”, we assume that the *Metaradiophrya* topological inconsistencies are to be ascribed to the plesiomorphic trap of the 18S rRNA gene, which was already discussed by Obert and Vďačný [12]. Our previous study [12] as well as the present analyses (Figs. 1, 2 and 3) indicate that this problem can be overcome by adding further molecular markers and by employing either the distance neighbor-joining method or the Bayesian approach implemented in Phycas.

Like the single gene analyses, the multi-gene approach also shows that the genus *Anoplophrya*, as delimited by Cépède [27] is polyphyletic. More specifically, *A. lumbrici* and *A. vulgaris* cluster together with members of the genus *Metaradiophrya*, while *S. nodulata* (traditionally classified in *Anoplophrya* by Cépède [27]) is placed within the paraphyletic group of astomes isolated from endogeic earthworms (Figs. 1, 2 and 3). Because Jankowski [28] fixed *A. lumbrici* as type species of *Anoplophrya*, *A. nodulata* (Dujardin, 1841) Cépède, 1910 [= *Leucophrys nodulata* Dujardin, 1841] needs to be transferred to a new genus, *Subanoplophrya*, which is proposed in the ‘Taxonomic summary’. This nomenclatural act is supported by morphological data as well. In fact, *S. nodulata* has two rows of contractile vacuoles, while *A. lumbrici* and *A. vulgaris* exhibit only one row.

### Putative secondary structure of the astome ITS2 molecule

On the basis of five astome species isolated from the gastrointestinal tube of lumbricid earthworms and five astome ciliates isolated from glossoscolecid and megascolecid earthworms, the putative secondary structure of the ITS2 molecule was proposed for ciliates of the subclass Astomatia for the first time (Figs. 4 and 5; Table 2 and Additional file 2: Figure S1, Additional file 3: Figure S2, Additional file 4: Figure S3, Additional file 5: Figure S4, and Additional file 6: Figure S5). The consensus structure consisted of a central loop bearing three helices corresponding to helices II, III, and IV of other ciliates [29–36]. As mentioned above, helix I might not be present in some astomes and helix E1 may be a variable constituent of their ITS2 molecules. Helix I is also quite likely absent in other ciliate groups, for instance, in some heterotricheans [37], peritrichs [30], and litostomateans [35, 36]. The presence of helix E1, which starts and closes the common loop of the ITS2 molecule, can be explained by the dynamic conformational model proposed by Côté et al. [38]. More specifically, the ring structure without helix E1 forms during the early stages of rRNA maturation, while the hairpin structure formed by helix E1 develops during the subsequent processing events.

In contrast to helices I and E1, the RNA logo analyses revealed highly conserved motifs in helices II–IV (Fig. 6). Structural and positional homology of these three helices is corroborated also by (1) the conserved GU pairings at the base of helix II and in the terminal stem of helix III, (2) several conserved unpaired spacer nucleotides between helices II and III, and (3) the highly conserved primary nucleotide structure of helix IV. Moreover, there is a pyrimidine-pyrimidine mismatch in helix II of all astome ciliates studied, which is also typical of most ciliates [29–31, 33, 34, 37, 39]. However, this highly characteristic mismatch is absent in spirotrichean [40–43] and litostomatean ciliates [32, 35, 36]. In astomes, helix III displays five or six bulges and a highly conserved region between bulge 3 and terminal loop (Figs. 4, 5 and 6 and Additional file 2: Figure S1, Additional file 3: Figure S2, Additional file 4: Figure S3, Additional file 5: Figure S4, and Additional file 6: Figure S5). Scuticociliates, which are considered to be the closest relatives of astome ciliates [11, 12, 14, 25], also exhibit a highly conserved region between bulge 3 and terminal loop of helix III, but possess only three or four bulges in helix III [29, 33, 34].

### Nucleotide and structural evolution of the ITS2 molecule in ciliates

The evolution of the ITS2 region is generally characterized by an increase in length and GC content [44]. However, this rule is hardly kept in ciliates due to the high variation in both parameters and no distinct trends in the length and GC content of the ITS2 region across the ciliate tree of life.

The length of the ITS2 molecule of astomes spans a comparatively narrow range from 182 to 194 nt (Table 2). Similar values have been reported from other oligohymenophorean main lineages as well: 168 to 169 nt in the genus *Paramecium* from the subclass Peniculia [39], 168 to 217 nt in the subclass Scuticociliatia [29, 33, 34], and 165 to 175 nt in the subclass Peritrichia [30, 31]. In contrast, ciliates from the phylogenetically unrelated classes Heterotrichea and Litostomatea have the shortest ITS2 molecules, having only 79–81 nt [37] and 100–112 nt [32, 35, 36, 45], respectively. However, ciliates from the class Spirotrichea, which is related to the class Litostomatea, exhibit ITS2 sequences of about 200 nt [40–43].

In addition, the ITS2 molecules of astome ciliates present a dramatic range of variation from 36.76% in *Metaracoelophrya* sp. to 53.80% in *Anoplophrya vulgaris* in terms of their GC content (Table 2). A similar wide range was also detected in various groups of phylogenetically closely or distantly related ciliates: from 31.40 to 47.93% in scuticociliates [29], from 23.56 to 48.48% in peritrichs [30], and from 23.64 to 49.00% in litostomateans [36]. Although heterotricheans represent a deep branching ciliate lineage, they have a surprisingly high GC content ranging from 54.3 to 62.0% [37].

In spite of the distinct ITS2 sequence variation in length and GC content, the majority of main ciliate groups shares a very similar pattern with homologous sequence segments in helices II and III, which have homologous locations. As mentioned previously by Miao et al. [29], insertions and deletions account for a large proportion of variability in the ITS2 region and the small size of spacer fragments may obviate the need for GC-rich DNA, or the high AT content might favor a structure analogous to that preferred by high GC content. Moreover, it appears that the overall size of the ITS2 is not critical for correct transcription, and the evolution of the ITS2 region might be governed by the “minimum-nucleotide formula” in ciliates [29], or in other words, a minimum number of necessary nucleotides [46]. There is an obvious conservation of the structural domains of helices II and III in ciliates [29–36, 40–43], which indicates that these two helices play a crucial functional role in the folding of the secondary structure of the ITS2 during rRNA primary transcript processing. However, helices I and IV vary in length and form, indicating that they may be less functionally constrained.

### Molecular discrimination of astome ciliates

The species-rich astome genera *Anoplophrya* and *Metaradiophrya* have a difficult taxonomic history [19–23] and their molecular taxonomy is only in its infancy [12]. The ITS region of the nuclear rRNA locus is one of the most frequently utilized markers in molecular taxonomy of ciliates (e.g., [31, 32, 40, 41, 47, 48]), because the rate of evolutionary changes in the ITS region is more than 100 times higher than that of the 18S rRNA gene [49]. The present study confirms that the ITS region is also a promising tool for species discrimination of astome ciliates. Indeed, by using the secondary structure and CBCs analyses of the ITS2 molecule, we could unambiguously delimit five astome species isolated from the lumbricid earthworms. Occurrence of these five astome taxa is also correlated with ecological groups of their host earthworms (Figs. 1, 2 and 3). However, our previous morphometric analyses were not able to separate congeners in the genera *Metaradiophrya* and *Anoplophrya* [12]. Therefore, the morphology-based taxonomy of astome ciliates needs to be tested in the light of molecular and ecological data as well.

Genetic p-distances and the presence of CBCs are two molecular indicators that are most often utilized in delimiting species boundaries between closely related taxa. Although the p-distance criteria are arbitrary and often fail to identify species [37, 50], the CBC criterion tends to be well-correlated with species boundaries in both sexual [51, 52] and asexual organisms [53]. Specifically, occurrence of a single CBC within a helix can differentiate two species with a probability of 0.93, however, the probability decreases to 0.76 when there is no CBC [53].

With regards to the five astome ciliate species isolated from lumbricid earthworms, either just a very small intraspecific sequence variability (up to 0.1%) or none at all was revealed within individual species both for the 18S rRNA gene [12] and the ITS region + D1/D2-28S rRNA gene sequences (present study). The genetic p-distances among the five astome species ranged from 1.32 to 5.95% in the 18S rRNA gene, and from 4.11 to 18.31% in the ITS1–5.8S-ITS2-28S rRNA gene region. Within this region, the highest intraspecific distances were detected in the ITS1 sequences followed by the ITS2 and the D1/D2-28S rRNA gene sequences (Table 3). This shows that these three molecular markers possess a much higher power to discriminate between astome species than the 18S rRNA gene.

**Table 3:** Interspecific sequence p-distances of the 18S, 5.8S, and D1/D2-28S rRNA genes (below diagonal) as well as of the ITS1, ITS2, and ITS region + D1/D2-28S RNA gene sequences (above diagonal) among five astome taxa

	1.	2.	3.	4.	5.
1. *Metaradiophrya lumbrici*	–	0.2050/0.0585/0.0411	0.3050/0.1765/0.0810	0.3250/0.2033/0.0945	0.4150/0.2834/0.1669
2. *Metaradiophrya varians*	0.0132/0.0000/0.0294	–	0.2470/0.1799/0.0801	0.2600/0.2065/0.0928	0.4750/0.3194/0.1727
3. *Anoplophrya lumbrici*	0.0287/0.0134/0.0523	0.0337/0.0134/0.0556	–	0.1430/0.0870/0.0490	0.4560/0.3245/0.1831
4. *Anoplophrya vulgaris*	0.0276/0.0134/0.0665	0.0314/0.0134/0.0687	0.0160/0.0000/0.0414	–	0.4810/0.2826/0.1809
5. *Subanoplophrya nodulata*	0.0508/0.0201/0.1442	0.0518/0.0201/0.1398	0.0595/0.0201/0.1564	0.0573/0.0201/0.1608	–

The reliability of the *Anoplophrya/Subanoplophrya* species was also strongly corroborated by the presence of two to five CBCs in helix III (Fig. 7). Although no CBCs were detected between the two *Metaradiophrya* species, they distinctly differed by the structure and nucleotide composition of helix I of their ITS2 molecules (Fig. 5). In summary, the five astome species can be clearly separated by primary structure of all tested markers of the rRNA locus as well as by the secondary structure of the ITS2 molecules.

### Evolution of astome ciliates

Molecular phylogeny of astome ciliates conflicts with traditional classifications based on the presence and characteristics of the attachment apparatus [54], as already recognized by Fokam et al. [25]. Although the sampling of astome ciliates is quite limited, all phylogenetic analyses suggest an interesting eco-evolutionary trend. The astome *Haptophrya planariarum*, which is isolated from flatworms, branches off first, and all astomes isolated from annelids form a strongly statistically supported monophylum [11, 14]. Astomes from polychaetes (*Durchoniella* spp.) are depicted as a sister to the paraphyletic assemblage of astomes from endogeic oligochaetes (*Almophrya*, *Subanoplophrya*, *Eudrilophrya*, *Metaracoelophrya*, *Njinella*, and *Paraclausilocola*). Lineages of astome ciliates from anecic (*M. lumbrici* and *A. lumbrici*) and epigeic (*M. varians* and *A. vulgaris*) earthworms are nested within the crown radiation of this paraphyletic endogeic cluster [11–14, 25]. Interestingly, endogeic earthworms also form a paraphyletic group that contains anecic and epigeic lineages. Character reconstruction analyses conducted by Domínguez et al. [55] revealed that the endogeic lifestyle was the ancestral life history trait of earthworms and both epigeic and anecic earthworms evolved from endogeic antecessors multiple times. Whether the diversification of astomes, which inhabit the digestive tube of earthworms, was driven by the divergence of their host organisms or not, it is still an exciting emerging topic that needs to be tested by distinctly increased sampling of astome ciliates from various host groups and geographic regions.

The phylogenetic position of the subclass Astomatia within the highly diverse class Oligohymenophorea is another interesting issue. According to 18S rRNA gene phylogenies, the subclass Astomatia is most closely related to the subclass Scuticociliatia, although the statistical support is poor [11–14, 25]. The subclass Scuticociliatia is depicted paraphyletic, and some members of the non-monophyletic order Loxocephalida cluster with the astome *Haptophrya planariarum* in the 18S rRNA gene trees [11, 12, 14]. The present multigene phylogenies also indicate a close relationship of the Astomatia and the Scuticociliatia, however, their kinships could not be robustly solved (Fig. 3). The comparison of the secondary structure of the scuticociliate ITS2 molecules might cast some light on this problem. The consensus structure of the ITS2 molecule exhibits helix IV only in the scuticociliate order Loxocephalida, similarly to the subclass Astomatia. The single significant structural difference between loxocephalids and astomes is the number of bulges in helix III (4 in loxocephalids vs. 5 or 6 in astomes).

### Associations of astome ciliates with their hosts

Although this is still an insufficiently explored topic, our pioneer studies, which used the morpho-molecular approach [11, 12, 14], indicate that at least some astome species are associated with a certain systematic and/or ecological group of their host organisms. More specifically, *Haptophrya planariarum* (von Siebold, 1839) Stein, 1867 has so far been reported only from freshwater tricladid planarians and all isolates cluster together in 18S rRNA gene phylogenies [11, 14]. Likewise, members of the genus *Durchoniella* de Puytorac, 1954 have been detected only in polychaetes [28, 56] and three *Durchoniella* species isolated from the polychaete *Cirriformia tentaculata* (Montagu, 1808) also group together [13]. Astomes detected in anecic and epigeic earthworms form monophyla within the genera *Anoplophrya* and *Metaradiophrya* in multi-gene phylogenetic trees ([12], present study). However, the deeper branching lineages of astomes that inhabit endogeic earthworms show a weaker phylogenetic host specificity. Primarily, astomes isolated from endogeic lumbricid earthworms are placed among astomes from endogeic glossoscolecid and megascolecid earthworms, and multiple astome species may colonize the same earthworm species ([12, 25], present study). The endogeic lifestyle was the ancestral strategy in earthworms. Moreover, glossoscolecids and megascolecids branch deeper in phylogenetic trees than lumbricids [55]. This indicates that astome ciliates may conform to Szidat’s rule, which states that deeper branching hosts harbor deeper branching symbionts [57]. Nevertheless, much more molecular data are needed to test whether Szidat’s rule holds for astome ciliates and their earthworm hosts.

## Taxonomic summary

### *Subanoplophrya* gen. n.

#### ZooBank registration number of present work

urn:lsid:zoobank.org:pub:F5F52F1C-829A-4E0A-B1FE-1A04B84A2025.

#### Zoobank registration number of new genus

urn:lsid:zoobank.org:act:AEB435AD-8DEF-4AB2-9277-5B11338B6C3D.

#### Diagnosis

*Anoplophrya*-like Astromatida with two rows of contractile vacuoles. No attachment apparatus. Lives in digestive tract of endogeic lumbricid earthworms.

#### Type species

*Leucophrys nodulata* Dujardin, 1841 [= *Anoplophrya nodulata* (Dujardin, 1841) Cépède, 1910].

#### Etymology

Composite of the Latin prefix *sub*- (under, below) and the generic name *Anoplophrya*, alluding that *S. nodulata* is deeper branching than *A. lumbrici* in phylogenetic trees. Feminine gender.

#### Remarks

The new genus is established in order to solve the polyphyly problem of *Anoplophrya*. Specifically, *A. lumbrici* (type species of the genus *Anoplophrya*) and *A. vulgaris* cluster together with members of the genus *Metaradiophrya*, while *S. nodulata* (traditionally classified in *Anoplophrya* by Cépède [27]) is placed as a distinct lineage within the paraphyletic assemblage of astomes isolated from endogeic earthworms. The establishment of *Subanoplophrya* is also supported by morphological data, i.e., *Subanoplophrya* possesses two rows of contractile vacuoles, while *Anoplophrya* exhibits only a single row.

## Conclusions

In this research, we report nineteen ITS1–5.8S-ITS2 region and D1/D2-28S rRNA gene sequences from five astome species for the first time. Phylogenetic analyses of these molecular markers as well as the secondary structure analyses of the ITS2 molecule unequivocally support the distinctness of all five species. Moreover, the occurrence of the five astome taxa is perfectly correlated with ecological groups of their host earthworms: *S. nodulata* isolates from endogeic earthworms form a distinct clade, *A. lumbrici* specimens from anecic earthworms are sister to *A. vulgaris* individuals from epigeic earthworms, and *M. lumbrici* isolates from anecic earthworms are sister to *M. varians* specimens from epigeic earthworms. Phylogenetic trees also suggest a further evolutionary trend that astome ciliates might be associated with certain systematic groups of their host organisms: astomes from planarians branch off first and astomes from annelids form a monophylum in which astomes from polychaetes are sister to astomes from earthworms. Further molecular studies with distinctly increased taxon sampling are, however, required to assess the consistency of evolutionary associations of astomes with systematic/ecological groups of their host organisms more robustly.

## Methods

### Material collection and processing

In this study, we further analyzed the material collected in our previous contribution on the integrative taxonomy of astome ciliates, which inhabit the gastrointestinal tract of lumbricid earthworms from Central Europe [12]. Origin, identification, and dissection of the investigated lumbricids as well as morphological and numeric taxonomic methods used to study ciliates were described in detail by Obert and Vďačný [12].

### Molecular methods

Genomic DNA extracted from single cells in our previous study [12] was used to amplify the ITS1–5.8S-ITS2 region and the domains D1 and D2 of the 28S rRNA gene with the forward primer ITS-F (5′-GTA GGT GAA CCT GCG GAA GGA TCA TTA-3′) proposed by Miao et al. [29] and the reverse primer LO-R (5′-GCT ATC CTG AGR GAA ACT TCG-3′) designed by Pawlowski [58]. PCR included 5 μl of the extracted template DNA, 0.4 μl of the forward and reverse primers each (10 pmol/μl), and 10 μl of the GoTaq® Long PCR Master Mix (Promega, Fitchburg, Wisconsin, USA). The final volume was adjusted to 20 μl with deionized distilled water. PCR conditions were based on those in our previous study [12]: initial hot start denaturation at 95 °C for 15 min, 30 identical amplification cycles (denaturing at 95 °C for 45 s, annealing at 55 °C for 1 min, and extension at 72 °C for 2.5 min), and final extension at 72 °C for 10 min. The quality of the amplified DNA was verified by electrophoresing a 1% agarose gel. PCR products were purified using calf intestinal alkaline phosphatase and exonuclease I, *E. coli* (New England Biolabs® Inc.) and sequenced on an ABI 3730 automatic sequencer (Macrogen, Amsterdam, The Netherlands). Electropherograms of sequences were carefully examined in Chromas ver. 2.6.6 (Technelysium Pty Ltd.) and high-quality sequence fragments were assembled into contigs in BioEdit ver. 7.2.5 [59].

### Phylogenetic methods

Multi-sequence alignments were generated on the GUIDANCE2 server with the MAFFT algorithm and the following settings: 100 bootstrap repeats, the 6mer pairwise alignment method, and the maximum number of 100 iterations (URL: http://guidance.tau.ac.il/ver2/) [60]. Unmasked datasets and datasets masked with a cutoff value of 0.93 were analyzed in order to confirm the consistency of results. The first set of alignments included 20 astome taxa for which 18S rRNA gene and ITS region sequences are available in GenBank [12, 25]. The trees were rooted according to the work performed by Obert and Vďačný [12]. The second set of alignments comprised 1 peniculine, 15 astome, and 27 scuticociliate taxa for which 18S, 5.8S and 28S rRNA gene sequences are available [33, 34, 61]. Scuticociliates were selected because they are the closest relatives of astome ciliates within the class Oligohymenophorea according to literature [11, 14, 25, 62] as well as the BLASTn search. The peniculine *Paramecium tetraurelia* was used to a posteriori root the trees. Preliminary phylogenetic analyses of unmasked and masked datasets resulted in similar topologies. However, there were slightly higher nodal supports in trees inferred from the unmasked 18S rRNA gene + ITS region dataset and the masked 18S + 5.8S + 28S rRNA gene dataset. Therefore, only results obtained from these two alignments are presented. Both datasets, including the list of masked nucleotide positions, have been made freely available through Open Science Framework (DOI 10.17605/OSF.IO/SC53Q).

The best evolutionary substitution models were estimated and selected in jModelTest ver. 2.1.10 [63] under the Akaike Information Criterion on the CIPRES portal ver. 3.1 (URL: http://www.phylo.org/) [64]. The following parameters were used to run jModelTest: -s 11 -f -i -g 4 -t ML -AIC -AICc -BIC -DT -S BEST -p -a -w. Results are summarized in Additional file 7: Table S2 and Additional file 8: Table S3. Gene trees were constructed in the maximum likelihood and Bayesian frameworks. Maximum likelihood analyses were conducted in PHYML ver. 3.0 on the South of France bioinformatics platform (URL: http://www.atgc-montpellier.fr/phyml/), under the GTR + Γ + I evolutionary model and employing the SPR branch swapping algorithm. The reliability of branching patterns was assessed with 1000 non-parametric bootstrap replicates [65]. To confirm the consistency of results, two further likelihood methods were employed on the CIPRES portal: IQ-TREE on XSEDE ver. 1.6.10 using the implemented model tester [66] and RAxML-HPC BlackBox ver. 8.2.12 using the GTRGAMMA+I model [67]. Bayesian inferences were performed on the CIPRES portal in the program MrBayes on XSEDE ver. 3.2.6 [68]. Prior parameters of the GTR + Γ + I evolutionary model as estimated by jModelTest were implemented with the ‘lset’ and ‘prset’ commands in the MrBayes command block. Two simultaneous and independent Markov Chain Monte Carlo simulations were run for five million generations. Each run had four chains, one cold and three heated. The sampling frequency was set to one hundred and the burn-in fraction was specified as 25%. Convergence in Bayesian analyses was confirmed in that the average standard deviation of split frequencies was well below 0.01, the potential scale reduction factor approached 1, and no obvious trends were in the plots of generations versus log probability. Convergence and an adequate sample of the posterior were also checked using the R package RWTY [69]. To analyze the possibility of the “star-tree paradox”, another Bayesian method, as implemented in Phycas ver. 2.2 [70], was employed. The following settings were used to run the Phycas analyses: (1) the GTR + Γ + I evolutionary model as estimated by jModelTest, (2) the inverse gamma hyperprior with mean 2.1 and variance 0.90909 assigned to the hyperparameter *μ* governing the mean of the branch length prior, (3) the polytomy prior with C = exp.(1) favoring unresolved trees with polytomies over more-resolved trees when the difference is lower or equal to one likelihood unit on the log scale, (4) 10,000 burn-in cycles to autotune the updaters in MCMC simulations, (5) million cycles in MCMC simulations, and (6) sampling trees and parameters every 100 cycles. Two runs were launched for each set of analyses to confirm the consistency of the results obtained. Convergence and an adequate sample of the posterior were also checked using RWTY. Results of all RWTY analyses are summarized in Additional file 9: Figures S6–S18, Additional file 10: Figures S19–31, Additional file 11: Figures S32–44, and Additional file 12: Figures S45–57. The input and output files of MrBayes and Phycas analyses have been made freely available through Open Science Framework (DOI 10.17605/OSF.IO/SC53Q). All trees were computed as unrooted and were a posteriori rooted in FigTree ver. 1.2.3 (URL: http://tree.bio.ed.ac.uk/software/figtree/).

The ITS2 boundaries were determined by searching for the highly characteristic 5.8S–28S rRNA proximal stem of the ITS2 molecule [40]. Predictions of the putative secondary structure of the ITS2 molecules were carried out on the Mfold webserver ver. 3.0 (URL: http://unafold.rna.albany.edu/?q=mfold/), using the free-energy minimization approach [71]. All parameters were left default and only formation of the hybridized 5.8S–28S rRNA helix was forced during folding of the ITS2 molecules. Homologies of the secondary structures of the thermodynamically optimal ITS2 molecules were compared and manually edited in VARNA ver. 3.93 [72], taking into account the available ITS2 secondary structures of scuticociliates [29, 33, 34]. The numbers of conserved base pairs and unpaired bases in bulges and loops were counted and statistically evaluated for each structural domain of the ITS2 molecules. The base frequencies at each position of helices II–IV were calculated in the web program RNALogo (URL: http://rnalogo.mbc.nctu.edu.tw) [73]. The proposed ITS2 secondary structures were examined for CBCs with the CBCAnalyzer option [74] as implemented in 4SALE [75]. The consensus structure of the astome ITS2 molecule was also calculated in 4SALE.

Average between group p-distances among the five species of astome ciliates were calculated for the 18S, 5.8S, and D1/D2-28S rRNA genes as well as for the ITS1 and ITS2 sequences in MEGA X [76]. Pairwise deletion was used for alignment gaps.

## Supplementary information

**Additional file 1: Table S1.** Characterization of collection sites of earthworm species examined for the presence of astome ciliates.

**Additional file 2: Figure S1.** Putative secondary structure of the ITS2 molecule of *Almophrya bivacuolata*.

**Additional file 3: Figure S2.** Putative secondary structure of the ITS2 molecule of *Eudrilophrya complanata*.

**Additional file 4: Figure S3.** Putative secondary structure of the ITS2 molecule of *Metaracoelophrya* sp.

**Additional file 5: Figure S4.** Putative secondary structure of the ITS2 molecule of *Njinella prolifera*.

**Additional file 6: Figure S5.** Putative secondary structure of the ITS2 molecule of *Paraclausilocola constricta*.

**Additional file 7: Table S2** Evaluation of evolutionary substitution models fitted to the unmasked 18S rRNA gene + ITS region dataset, using the Akaike Information Criterion (AIC).

**Additional file 8: Table S3** Evaluation of evolutionary substitution models fitted to the 18S + 5.8S + 28S rRNA gene dataset masked with a cut-off value of 0.93, using the Akaike Information Criterion (AIC).

**Additional file 9: Figures S6–S18.** Results of RWTY analyses of MrBayes MCMC runs of the unmasked 18S rRNA gene + ITS region dataset.

**Additional file 10: Figures S19–S31.** Results of RWTY analyses of MrBayes MCMC runs of the 18S + 5.8S +28S rRNA gene dataset masked with a cut-off value of 0.93.

**Additional file 11: Figures S32–S44.** Results of RWTY analyses of Phycas MCMC runs of the unmasked 18S rRNA gene + ITS region dataset.

**Additional file 12: Figures S45–S57.** Results of RWTY analyses of Phycas MCMC runs of the 18S + 5.8S +28S rRNA gene dataset masked with a cut-off value of 0.93.

## Data Availability

New sequences have been deposited in the NCBI database under the accessions: MN897871–MN897889. Results of all analyses are included in this published article and in Additional file 1: Table S1, Additional file 2: Figure S1, Additional file 3: Figure S2, Additional file 4: Figure S3, Additional file 5: Figure S4, Additional file 6: Figure S5, Additional file 7: Table S2, Additional file 8: Table S3, Additional file 9: Figures S6–S18, Additional file 10: Figures S19–S31, Additional file 11: Figures S32–S44, Additional file 12: Figures S45–S57. In addition, the alignments used for phylogenetic inference and the input and output files of Bayesian analyses have been made freely available through Open Science Framework (DOI 10.17605/OSF.IO/SC53Q).

## Abbreviations

18S rRNA: small subunit ribosomal RNA
28S rRNA: large subunit ribosomal RNA
BI: Bayesian inference
bp: base pairs
CBC: Compensatory base change
GC: Guanine-cytosine
GTR + Γ + I: General time reversible model + gamma distribution of nucleotide substitutions + proportion of invariable sites
ITS: Internal transcribed spacer
ML: Maximum likelihood
nt: nucleotide
PCR: Polymerase chain reaction
