# Supplementary material for: Delimitation of five astome ciliate species isolated from the digestive tube of three ecologically different groups of lumbricid earthworms, using the internal transcribed spacer region and the hypervariable D1/D2 region of the 28S rRNA gene
Source: BMC Evol Biol. 2020 Mar 14;20:37. doi: 10.1186/s12862-020-1601-2 (PMC7071660; doi:10.1186/s12862-020-1601-2)
Supplement: Supplementary file 1 — Additional file 1: Table S1. Characterization of collection sites of earthworm species examined for the presence of astome ciliates. [file 12862_2020_1601_MOESM1_ESM.pdf]

Additional file 1: Table S1 Characterization of collection sites of earthworm species examined for the presence of astome ciliates

Collection date[a]	Collection site	Locality code	GPS coordinates	Host species
6/6/2017	Agricultural, brown soil from a garden, Šúrska ulica street, Rendez, Bratislava	RZ	48°11'57.6"N 17°10'25.0"E	*Lumbricus terrestris* Linné, 1758
10/3/ 2017	Floodplain soil from a riparian, willow-poplar forest near the Karlova Ves branch of the Danube river, Bratislava	KR	48°08'47.5"N 17°04'08.0"E	*Lumbricus terrestris* Linné, 1758
5/19/2018	Decomposing plant material from a compost heap in the Botanical Garden, Karlova Ves, Bratislava	BZ	48°08'43.5"N 17°04'21.1"E	*Eisenia fetida* (Savigny, 1826)
6/28/2018	Decomposing plant material and humous soil from a garden compost heap, Jakubská ulica street, Rača, Bratislava	JA-1	48°12'10.9"N 17°09'05.7"E	*Eisenia fetida* (Savigny, 1826)
6/28/2018	Loamy soil with fallen needles in the surroundings of a garden wall, Jakubská ulica street, Rača, Bratislava	JA-2	48°12'12.2"N 17°09'03.1"E	*Lumbricus terrestris* Linné, 1758
6/30/2018	Humous soil with high content of decomposing plant material from a garden at the foothill of the Malé Karpaty Mts., Na Grunte street, Krasňany, Bratislava	NG	48°11'44.4"N 17°07'47.5"E	*Dendrobaena veneta* (Rosa, 1886)
7/2/2018	Upper 50 cm turf layer in the riparian zone of the Rašelinisko pond in the vicinity of the Pusté Úľany village, Galanta district	PU	48°13'21.9"N 17°34'49.9"E	*Octolasion tyrtaeum* (Savigny, 1826)

[a] Dates are given as mo/d/yr.
