# Supplementary material for: Delimitation of five astome ciliate species isolated from the digestive tube of three ecologically different groups of lumbricid earthworms, using the internal transcribed spacer region and the hypervariable D1/D2 region of the 28S rRNA gene
Source: BMC Evol Biol. 2020 Mar 14;20:37. doi: 10.1186/s12862-020-1601-2 (PMC7071660; doi:10.1186/s12862-020-1601-2)

Additional file 4: Figure S3 Putative secondary structure of the ITS2 molecule of *Metaracoelophrya* sp.

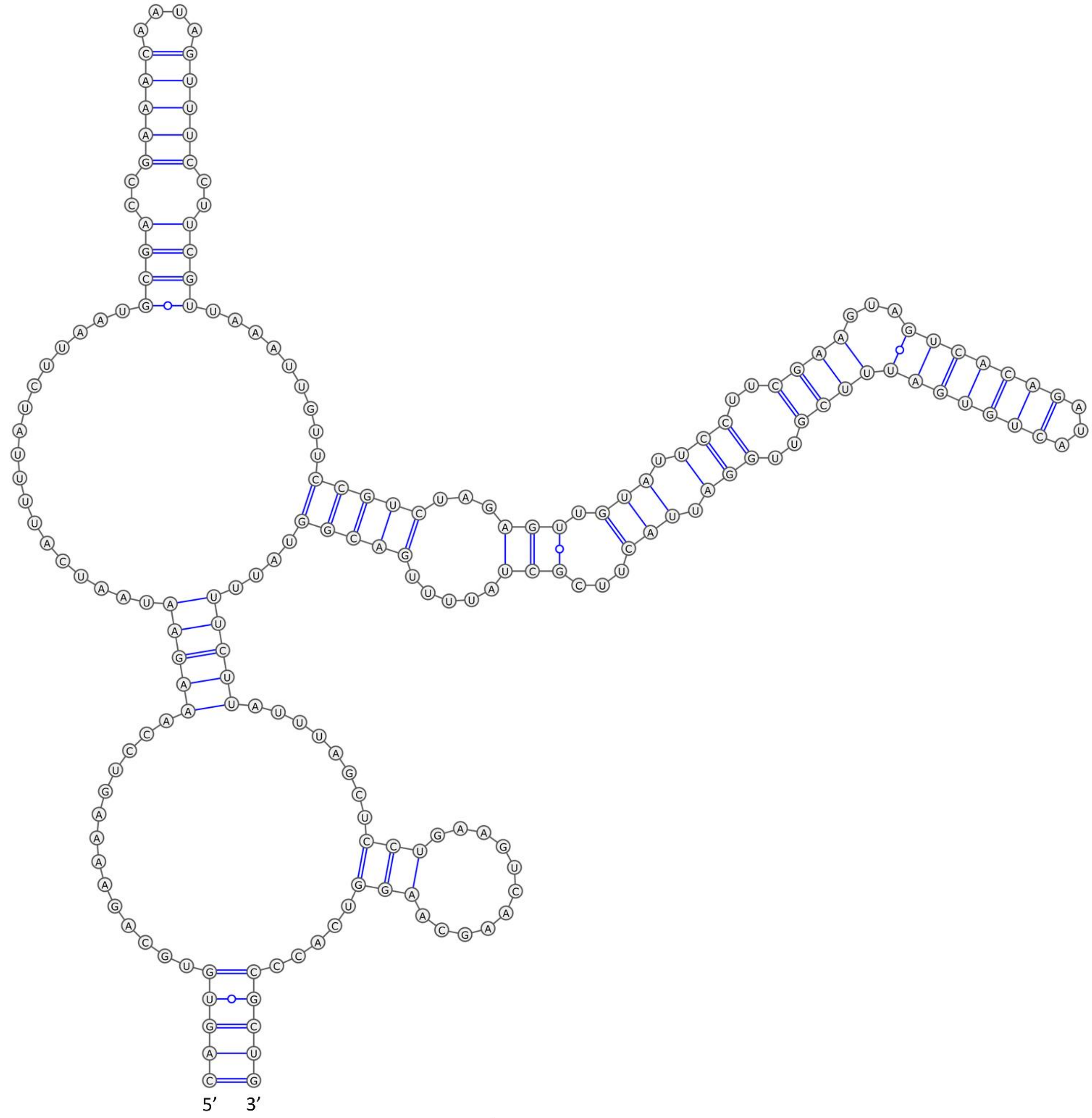

Supplement: Supplementary file 4 — Additional file 4: Figure S3. Putative secondary structure of the ITS2 molecule of Metaracoelophrya sp. [file 12862_2020_1601_MOESM4_ESM.pdf]
