# Supplementary material for: Delimitation of five astome ciliate species isolated from the digestive tube of three ecologically different groups of lumbricid earthworms, using the internal transcribed spacer region and the hypervariable D1/D2 region of the 28S rRNA gene
Source: BMC Evol Biol. 2020 Mar 14;20:37. doi: 10.1186/s12862-020-1601-2 (PMC7071660; doi:10.1186/s12862-020-1601-2)

**Additional file 6: Figure S5** Putative secondary structure of the ITS2 molecule of *Paraclausilocola constricta*

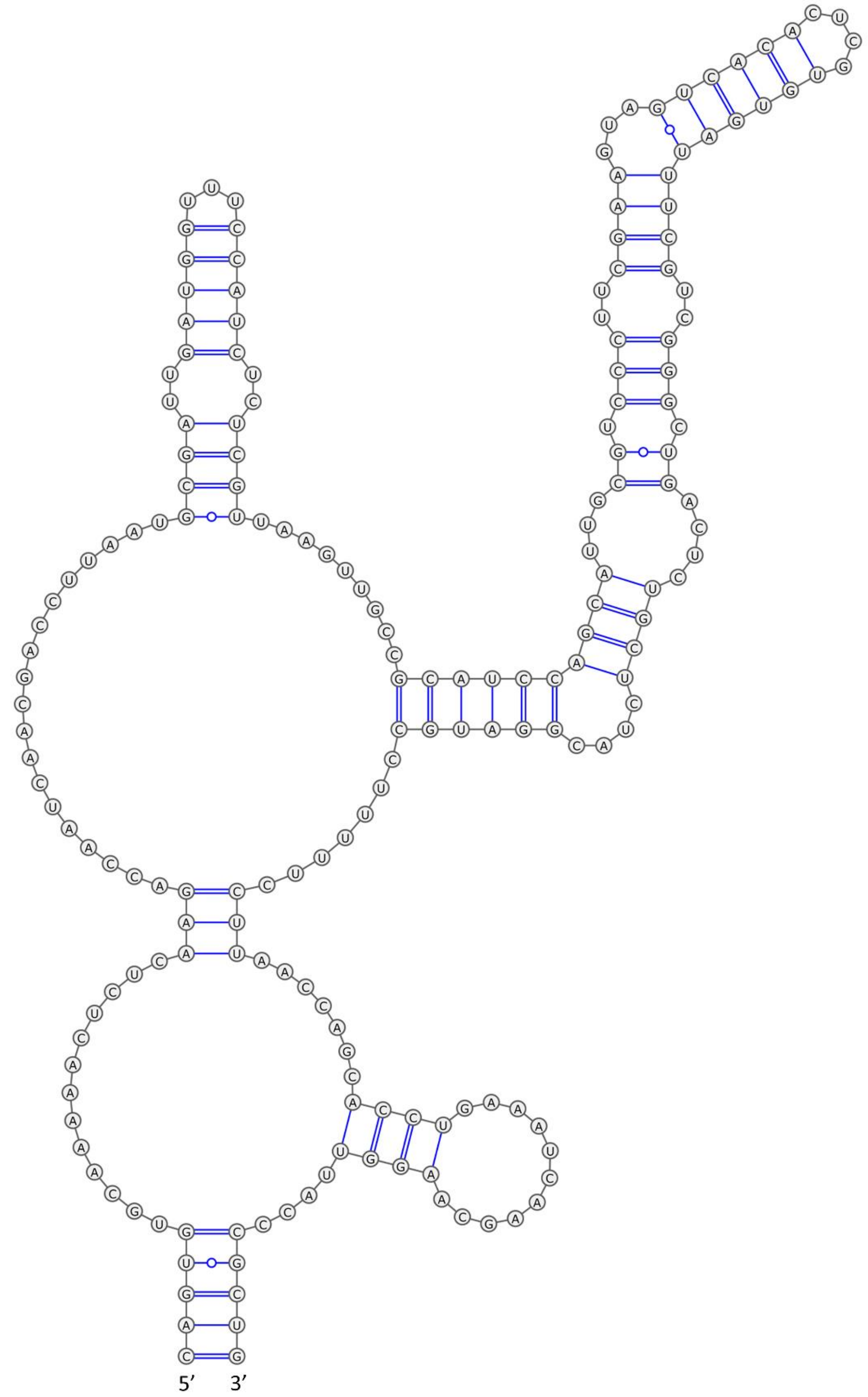

Supplement: Supplementary file 6 — Additional file 6: Figure S5. Putative secondary structure of the ITS2 molecule of Paraclausilocola constricta. [file 12862_2020_1601_MOESM6_ESM.pdf]
