# Supplementary material for: Delimitation of five astome ciliate species isolated from the digestive tube of three ecologically different groups of lumbricid earthworms, using the internal transcribed spacer region and the hypervariable D1/D2 region of the 28S rRNA gene
Source: BMC Evol Biol. 2020 Mar 14;20:37. doi: 10.1186/s12862-020-1601-2 (PMC7071660; doi:10.1186/s12862-020-1601-2)
Supplement: Supplementary file 7 — Additional file 7: Table S2 Evaluation of evolutionary substitution models fitted to the unmasked 18S rRNA gene + ITS region dataset, using the Akaike Information Criterion (AIC). [file 12862_2020_1601_MOESM7_ESM.pdf]

Additional file 7: Table S2 Evaluation of evolutionary substitution models fitted to the unmasked 18S rRNA gene + ITS region dataset, using the Akaike Information Criterion (AIC)

Evolutionary model	-lnL	K	AIC	Delta	Weight	Cumulative weight
GTR + I + G	5750.35660	46	11592.713200	0.0000000	0.759900	0.76
TIM2 + I + G	5749.50874	48	11595.017480	2.3042800	0.240100	1.00
TrN + I + G	5765.49835	45	11620.996700	28.283500	5.48E-07	1.00
TIM2 + I	5765.87868	45	11621.757360	29.044160	3.75E-07	1.00
TIM3 + I + G	5765.49469	46	11622.989380	30.276180	2.02E-07	1.00
TIM1 + I + G	5765.49818	46	11622.996360	30.283160	2.02E-07	1.00
GTR + I	5764.98132	47	11623.962640	31.249440	1.24E-07	1.00
SYM + I + G	5768.98553	45	11627.971060	35.257860	1.68E-08	1.00
TIM2ef + I + G	5771.43980	43	11628.879600	36.166400	1.06E-08	1.00
TPM2uf + I + G	5770.41052	45	11630.821040	38.107840	4.03E-09	1.00
TVM + I + G	5769.51636	47	11633.032720	40.319520	1.34E-09	1.00
TIM2 + G	5775.96668	45	11641.933360	49.220160	1.56E-11	1.00
GTR + G	5775.15393	47	11644.307860	51.594660	4.75E-12	1.00
TrN + I	5782.05292	44	11652.105840	59.392640	9.63E-14	1.00
SYM + I	5782.55588	44	11653.111760	60.398560	5.83E-14	1.00
TPM2uf + I	5782.85672	44	11653.713440	61.000240	4.31E-14	1.00
TIM3 + I	5781.88881	45	11653.777620	61.064420	4.18E-14	1.00
TIM2ef + I	5784.89386	42	11653.787720	61.074520	4.16E-14	1.00
TIM1 + I	5782.04315	45	11654.086300	61.373100	3.58E-14	1.00
TVM + I	5781.95963	46	11655.919260	63.206060	1.43E-14	1.00
HKY + I + G	5784.09814	44	11656.196280	63.483080	1.25E-14	1.00
TPM3uf + I + G	5784.05197	45	11658.103940	65.390740	4.80E-15	1.00
TPM1uf + I + G	5784.09436	45	11658.188720	65.475520	4.60E-15	1.00
TVMef + I + G	5786.29840	44	11660.596800	67.883600	1.38E-15	1.00
TPM2 + I + G	5788.64450	42	11661.289000	68.575800	9.77E-16	1.00
TIM3ef + I + G	5790.12589	43	11666.251780	73.538580	8.17E-17	1.00
TrNef + I + G	5791.57448	42	11667.148960	74.435760	5.21E-17	1.00
TIM1ef + I + G	5791.57236	43	11669.144720	76.431520	1.92E-17	1.00
TrN + G	5792.31887	44	11672.637740	79.924540	3.35E-18	1.00
SYM + G	5793.06460	44	11674.129200	81.416000	1.59E-18	1.00
TIM3 + G	5792.23684	45	11674.473680	81.760480	1.34E-18	1.00
TIM1 + G	5792.31241	45	11674.624820	81.911620	1.24E-18	1.00
TIM2ef + G	5795.55313	42	11675.106260	82.393060	9.76E-19	1.00
TVMef + I	5797.83821	43	11681.676420	88.963220	3.65E-20	1.00
TPM2 + I	5800.14259	41	11682.285180	89.571980	2.69E-20	1.00
TPM2uf + G	5797.38386	44	11682.767720	90.054520	2.12E-20	1.00
HKY + I	5798.89698	43	11683.793960	91.080760	1.27E-20	1.00

TVM + G	5796.56058	46	11685.121160	92.407960	6.53E-21	1.00
TPM3uf + I	5798.72326	44	11685.446520	92.733320	5.55E-21	1.00
TPM1uf + I	5798.89021	44	11685.780420	93.067220	4.69E-21	1.00
TIM3ef + I	5804.25485	42	11692.509700	99.796500	1.62E-22	1.00
TrNef + I	5805.74942	41	11693.498840	100.785640	9.90E-23	1.00
TIM1ef + I	5805.73279	42	11695.465580	102.752380	3.70E-23	1.00
TPM3 + I + G	5806.98505	42	11697.970100	105.256900	1.06E-23	1.00
K80 + I + G	5808.41375	41	11698.827500	106.114300	6.89E-24	1.00
TPM1 + I + G	5808.40451	42	11700.809020	108.095820	2.56E-24	1.00
TVMef + G	5812.17842	43	11710.356840	117.643640	2.16E-26	1.00
TPM2 + G	5814.58429	41	11711.168580	118.455380	1.44E-26	1.00
HKY + G	5813.54046	43	11713.080920	120.367720	5.54E-27	1.00
TIM3ef + G	5815.12387	42	11714.247740	121.534540	3.09E-27	1.00
TPM3uf + G	5813.45002	44	11714.900040	122.186840	2.23E-27	1.00
TPM1uf + G	5813.53798	44	11715.075960	122.362760	2.04E-27	1.00
TrNef + G	5816.75310	41	11715.506200	122.793000	1.65E-27	1.00
TIM1ef + G	5816.73443	42	11717.468860	124.755660	6.17E-28	1.00
TPM3 + I	5819.37530	41	11720.750600	128.037400	1.20E-28	1.00
TPM1 + I	5820.84061	41	11723.681220	130.968020	2.76E-29	1.00
K80 + I	5823.66101	40	11727.322020	134.608820	4.48E-30	1.00
TPM3 + G	5834.21321	41	11750.426420	157.713220	4.30E-35	1.00
K80 + G	5835.77661	40	11751.553220	158.840020	2.45E-35	1.00
TPM1 + G	5835.74948	41	11753.498960	160.785760	9.26E-36	1.00
F81 + I + G	5932.66443	43	11951.328860	358.615660	1.02E-78	1.00
F81 + I	5941.72147	42	11967.442940	374.729740	3.23E-82	1.00
JC + I + G	5952.90982	40	11985.819640	393.106440	3.30E-86	1.00
F81 + G	5954.23673	42	11992.473460	399.760260	1.19E-87	1.00
JC + I	5960.76743	39	11999.534860	406.821660	3.47E-89	1.00
JC + G	5973.05652	39	12024.113040	431.399840	1.60E-94	1.00
TIM2	6172.53705	44	12433.074100	840.360900	2.50E-183	1.00
GTR	6171.91029	46	12435.820580	843.107380	6.34E-184	1.00
TrN	6186.99219	43	12459.984380	867.271180	3.59E-189	1.00
TIM3	6186.86987	44	12461.739740	869.026540	1.49E-189	1.00
TIM1	6186.97511	44	12461.950220	869.237020	1.34E-189	1.00
SYM	6189.88388	43	12465.767760	873.054560	1.99E-190	1.00
TIM2ef	6192.30211	41	12466.604220	873.891020	1.31E-190	1.00
TPM2uf	6204.31490	43	12494.629800	901.916600	1.08E-196	1.00
TVM	6203.60420	45	12497.208400	904.495200	2.97E-197	1.00
TIM3ef	6208.32692	41	12498.653840	905.940640	1.44E-197	1.00
TrNef	6210.21879	40	12500.437580	907.724380	5.90E-198	1.00
TIM1ef	6210.11477	41	12502.229540	909.516340	2.41E-198	1.00
TVMef	6218.25424	42	12520.508480	927.795280	2.59E-202	1.00

TPM2	6220.68827	40	12521.376540	928.663340	1.68E-202	1.00
HKY	6218.97207	42	12521.944140	929.230940	1.26E-202	1.00
TPM3uf	6218.78594	43	12523.571880	930.858680	5.59E-203	1.00
TPM1uf	6218.95433	43	12523.908660	931.195460	4.72E-203	1.00
TPM3	6236.99904	40	12553.998080	961.284880	1.38E-209	1.00
K80	6238.89810	39	12555.796200	963.083000	5.62E-210	1.00
TPM1	6238.79109	40	12557.582180	964.868980	2.30E-210	1.00
F81	6340.03542	41	12762.070840	1169.357640	0.00E + 00	1.00
JC	6356.76924	38	12789.538480	1196.825280	0.00E + 00	1.00
