# Supplementary material for: Delimitation of five astome ciliate species isolated from the digestive tube of three ecologically different groups of lumbricid earthworms, using the internal transcribed spacer region and the hypervariable D1/D2 region of the 28S rRNA gene
Source: BMC Evol Biol. 2020 Mar 14;20:37. doi: 10.1186/s12862-020-1601-2 (PMC7071660; doi:10.1186/s12862-020-1601-2)
Supplement: Supplementary file 8 — Additional file 8: Table S3 Evaluation of evolutionary substitution models fitted to the 18S + 5.8S + 28S rRNA gene dataset masked with a cut-off value of 0.93, using the Akaike Information Criterion (AIC). [file 12862_2020_1601_MOESM8_ESM.pdf]

Additional file 8: Table S3 Evaluation of evolutionary substitution models fitted to the 18S + 5.8S + 28S rRNA gene dataset masked with a cut-off value of 0.93, using the Akaike Information Criterion (AIC)

Evolutionary model	-lnL	K	AIC	Delta	Weight	Cumulative weight
GTR + I + G	15184.06850	94	30556.137000	0.0000000	0.999962	1.00
TIM2 + I + G	15196.26173	92	30576.523460	20.386460	3.74E-05	1.00
TIM3 + I + G	15201.19719	92	30586.394380	30.257380	2.69E-07	1.00
TVM + I + G	15201.76446	93	30589.528920	33.391920	5.61E-08	1.00
TIM1 + I + G	15202.82872	92	30589.657440	33.520440	5.26E-08	1.00
TrN + I + G	15210.50432	91	30603.008640	46.871640	6.64E-11	1.00
TPM2uf + I + G	15215.77803	91	30613.556060	57.419060	3.40E-13	1.00
TPM3uf + I + G	15218.17769	91	30618.355380	62.218380	3.09E-14	1.00
TPM1uf + I + G	15221.12930	91	30624.258600	68.121600	1.61E-15	1.00
HKY + I + G	15228.09252	90	30636.185040	80.048040	4.15E-18	1.00
SYM + I + G	15232.64232	91	30647.284640	91.147640	1.61E-20	1.00
TVMef + I + G	15236.04866	90	30652.097320	95.960320	1.45E-21	1.00
GTR + G	15243.93584	93	30673.871680	117.734680	2.72E-26	1.00
TIM2 + G	15256.71216	91	30695.424320	139.287320	5.68E-31	1.00
TIM3 + G	15260.17653	91	30702.353060	146.216060	1.78E-32	1.00
TIM1 + G	15262.78991	91	30707.579820	151.442820	1.30E-33	1.00
TVM + G	15264.76510	92	30713.530200	157.393200	6.64E-35	1.00
TrN + G	15270.56396	90	30721.127920	164.990920	1.49E-36	1.00
TIM3ef + I + G	15273.95619	89	30725.912380	169.775380	1.36E-37	1.00
TPM3 + I + G	15277.05599	88	30730.111980	173.974980	1.67E-38	1.00
TPM2uf + G	15278.20283	90	30736.405660	180.268660	7.16E-40	1.00
TIM2ef + I + G	15279.28698	89	30736.573960	180.436960	6.59E-40	1.00
TPM2 + I + G	15282.34882	88	30740.697640	184.560640	8.38E-41	1.00
TPM3uf + G	15283.65505	90	30747.310100	191.173100	3.07E-42	1.00
TPM1uf + G	15284.58752	90	30749.175040	193.038040	1.21E-42	1.00
HKY + G	15291.11488	89	30760.229760	204.092760	4.81E-45	1.00
SYM + G	15291.50976	90	30763.019520	206.882520	1.19E-45	1.00
TVMef + G	15296.23237	89	30770.464740	214.327740	2.88E-47	1.00
TIM1ef + I + G	15306.19484	89	30790.389680	234.252680	1.36E-51	1.00
TPM1 + I + G	15309.29398	88	30794.587960	238.450960	1.66E-52	1.00
TrNef + I + G	15317.31812	88	30810.636240	254.499240	5.45E-56	1.00
K80 + I + G	15320.08959	87	30814.179180	258.042180	9.26E-57	1.00
TIM3ef + G	15334.57482	88	30845.149640	289.012640	1.74E-63	1.00
TPM3 + G	15339.04411	87	30852.088220	295.951220	5.43E-65	1.00
TIM2ef + G	15340.06950	88	30856.139000	300.002000	7.17E-66	1.00
TPM2 + G	15344.38443	87	30862.768860	306.631860	2.60E-67	1.00

TIM1ef + G	15367.43127	88	30910.862540	354.725540	9.38E-78	1.00
TPM1 + G	15373.52808	87	30921.056160	364.919160	5.74E-80	1.00
TrNef + G	15379.10053	87	30932.201060	376.064060	2.18E-82	1.00
K80 + G	15383.16188	86	30938.323760	382.186760	1.02E-83	1.00
F81 + I + G	15522.70440	89	31223.408800	667.271800	1.27E-145	1.00
F81 + G	15581.85987	88	31339.719740	783.582740	7.03E-171	1.00
GTR + I	15585.18169	93	31356.363380	800.226380	1.71E-174	1.00
JC + I + G	15604.19053	86	31380.381060	824.244060	1.04E-179	1.00
TIM2 + I	15599.34019	91	31380.680380	824.543380	8.97E-180	1.00
TIM3 + I	15603.55695	91	31389.113900	832.976900	1.32E-181	1.00
TIM1 + I	15607.49518	91	31396.990360	840.853360	2.58E-183	1.00
TVM + I	15610.43617	92	31404.872340	848.735340	5.01E-185	1.00
TrN + I	15615.48390	90	31410.967800	854.830800	2.38E-186	1.00
TPM2uf + I	15624.21498	90	31428.429960	872.292960	3.84E-190	1.00
TPM3uf + I	15630.41484	90	31440.829680	884.692680	7.79E-193	1.00
TPM1uf + I	15633.83990	90	31447.679800	891.542800	2.53E-194	1.00
HKY + I	15641.93869	89	31461.877380	905.740380	2.09E-197	1.00
SYM + I	15644.02801	90	31468.056020	911.919020	9.53E-199	1.00
TVMef + I	15651.84316	89	31481.686320	925.549320	1.05E-201	1.00
JC + G	15663.70886	85	31497.417720	941.280720	0.00E+00	1.00
TIM3ef + I	15680.18055	88	31536.361100	980.224100	1.40E-213	1.00
TIM2ef + I	15684.81748	88	31545.634960	989.497960	1.36E-215	1.00
TPM3 + I	15688.22642	87	31550.452840	994.315840	1.22E-216	1.00
TPM2 + I	15692.48495	87	31558.969900	1002.832900	0.00E+00	1.00
TIM1ef + I	15708.33257	88	31592.665140	1036.528140	0.00E+00	1.00
TPM1 + I	15716.25579	87	31606.511580	1050.374580	0.00E+00	1.00
TrNef + I	15718.53563	87	31611.071260	1054.934260	0.00E+00	1.00
K80 + I	15726.43075	86	31624.861500	1068.724500	0.00E+00	1.00
F81 + I	15912.54197	88	32001.083940	1444.946940	0.00E+00	1.00
JC + I	15994.24323	85	32158.486460	1602.349460	0.00E+00	1.00
GTR	16756.22741	92	33696.454820	3140.317820	0.00E+00	1.00
TIM2	16766.99094	90	33713.981880	3157.844880	0.00E+00	1.00
TIM3	16767.84395	90	33715.687900	3159.550900	0.00E+00	1.00
TIM1	16769.86078	90	33719.721560	3163.584560	0.00E+00	1.00
TrN	16776.53990	89	33731.079800	3174.942800	0.00E+00	1.00
TVM	16796.90524	91	33775.810480	3219.673480	0.00E+00	1.00
TPM2uf	16808.04306	89	33794.086120	3237.949120	0.00E+00	1.00
TPM3uf	16809.10559	89	33796.211180	3240.074180	0.00E+00	1.00
TPM1uf	16811.45230	89	33800.904600	3244.767600	0.00E+00	1.00
HKY	16818.18253	88	33812.365060	3256.228060	0.00E+00	1.00
SYM	16832.43429	89	33842.868580	3286.731580	0.00E+00	1.00
TVMef	16848.42518	88	33872.850360	3316.713360	0.00E+00	1.00

TIM3ef	16858.12603	87	33890.252060	3334.115060	0.00E+00	1.00
TIM2ef	16870.06868	87	33914.137360	3358.000360	0.00E+00	1.00
TPM3	16874.35541	86	33920.710820	3364.573820	0.00E+00	1.00
TIM1ef	16883.27642	87	33940.552840	3384.415840	0.00E+00	1.00
TPM2	16885.83622	86	33943.672440	3387.535440	0.00E+00	1.00
TrNef	16893.21082	86	33958.421640	3402.284640	0.00E+00	1.00
TPM1	16899.32060	86	33970.641200	3414.504200	0.00E+00	1.00
K80	16909.22740	85	33988.454800	3432.317800	0.00E+00	1.00
F81	17074.68965	87	34323.379300	3767.242300	0.00E+00	1.00
JC	17162.24314	84	34492.486280	3936.349280	0.00E+00	1.00
