# Supplementary material for: Delimitation of five astome ciliate species isolated from the digestive tube of three ecologically different groups of lumbricid earthworms, using the internal transcribed spacer region and the hypervariable D1/D2 region of the 28S rRNA gene
Source: BMC Evol Biol. 2020 Mar 14;20:37. doi: 10.1186/s12862-020-1601-2 (PMC7071660; doi:10.1186/s12862-020-1601-2)
Supplement: Supplementary file 9 — Additional file 9: Figures S6–S18. Results of RWTY analyses of MrBayes MCMC runs of the unmasked 18S rRNA gene + ITS region dataset. [file 12862_2020_1601_MOESM9_ESM.pdf]

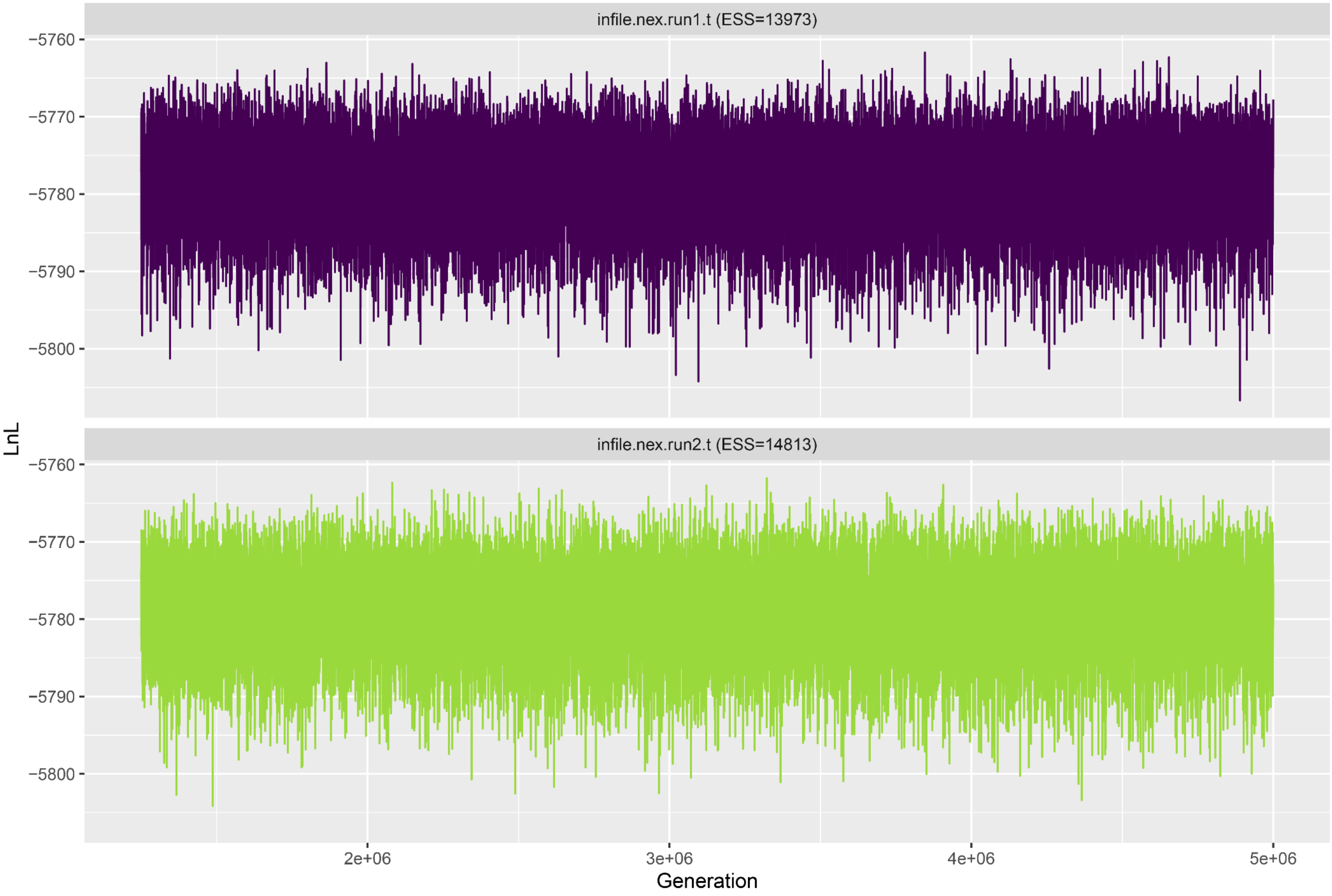

Figure S6. Trace plots of log likelihood (lnL) of phylogenetic trees of two runs of the MrBayes MCMC analyses of the unmasked 18S rRNA gene + ITS region dataset

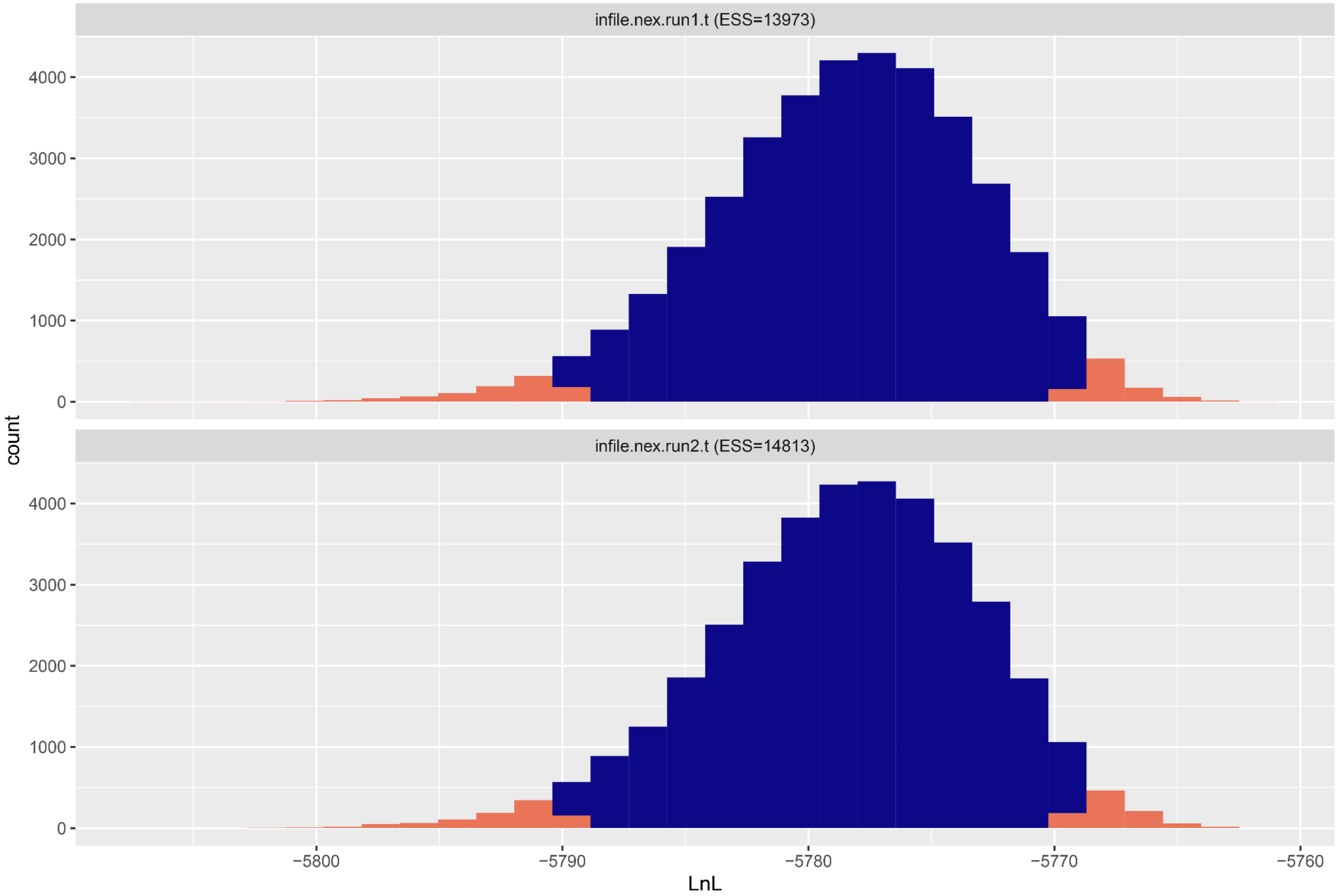

Figure S7. Density plots of log likelihood (lnL) of phylogenetic trees of two runs of the MrBayes MCMC analyses of the unmasked 18S rRNA gene + ITS region dataset. Red values indicate values outside the 95% credibility intervals

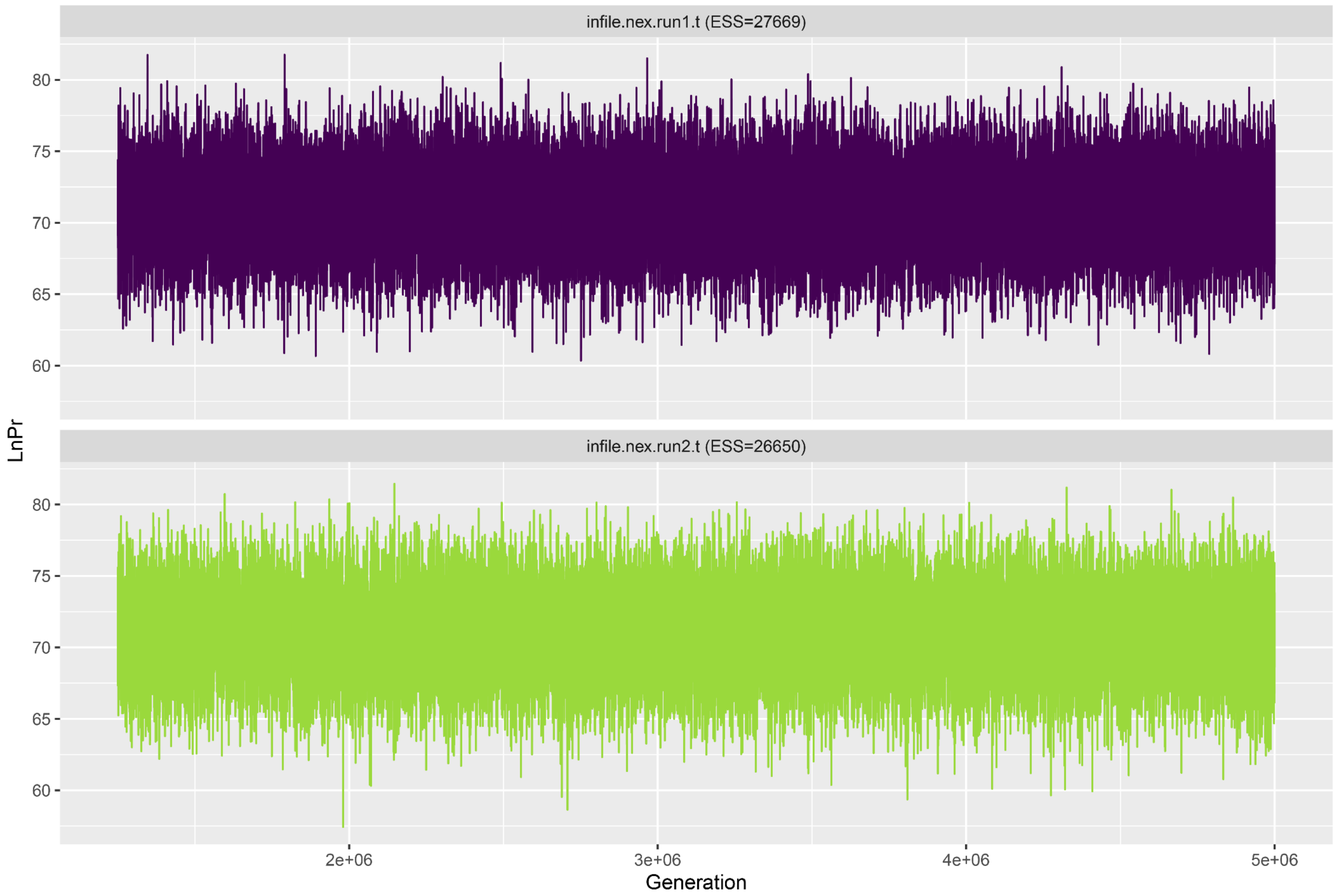

Figure S8. Trace plots of log model parameters (lnPr) of two runs of the MrBayes MCMC analyses of the unmasked 18S rRNA gene + ITS region dataset

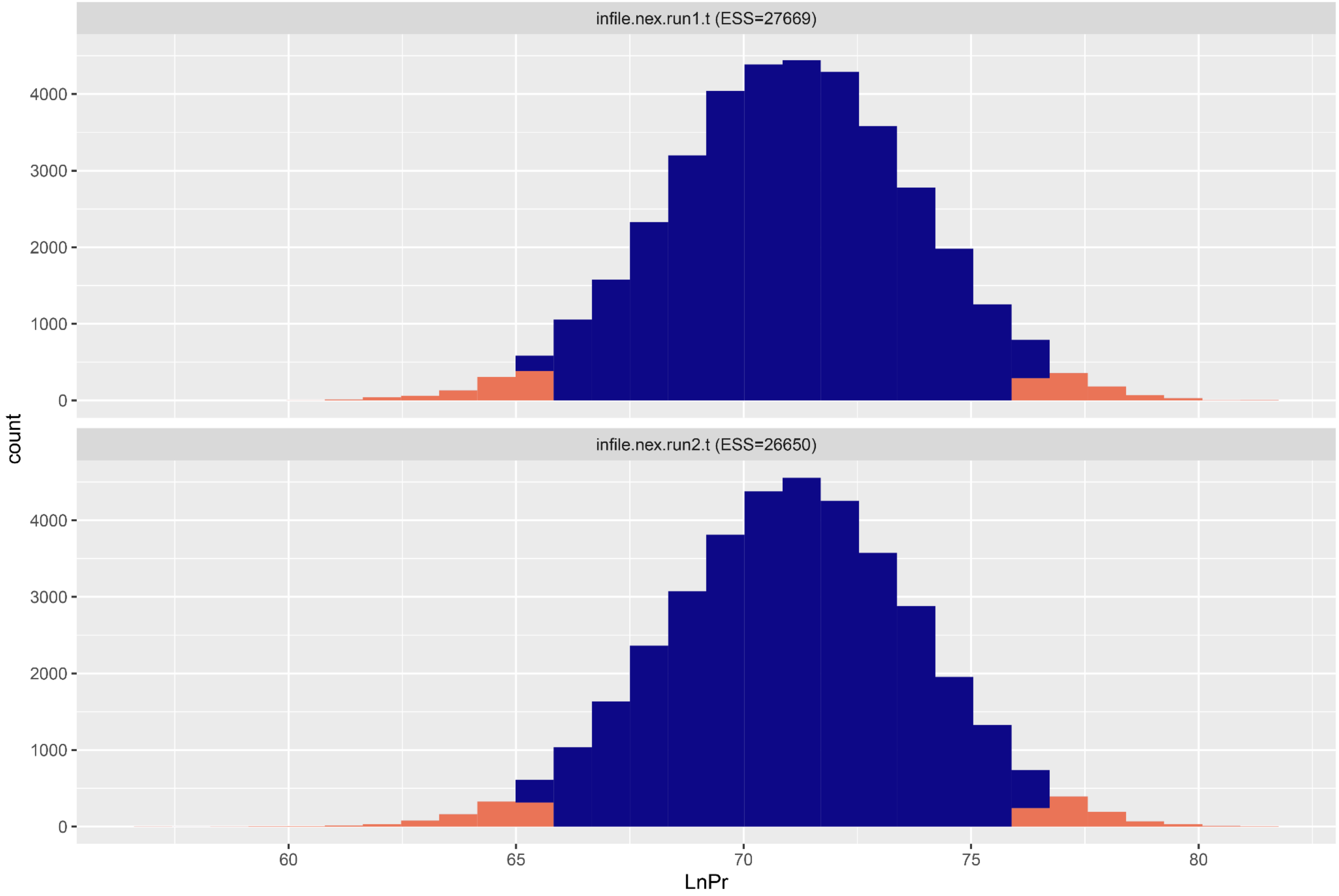

Figure S9. Density plots of log model parameters (lnPr) of two runs of the MrBayes MCMC analyses of the unmasked 18S rRNA gene + ITS region dataset. Red values indicate values outside the 95% credibility intervals

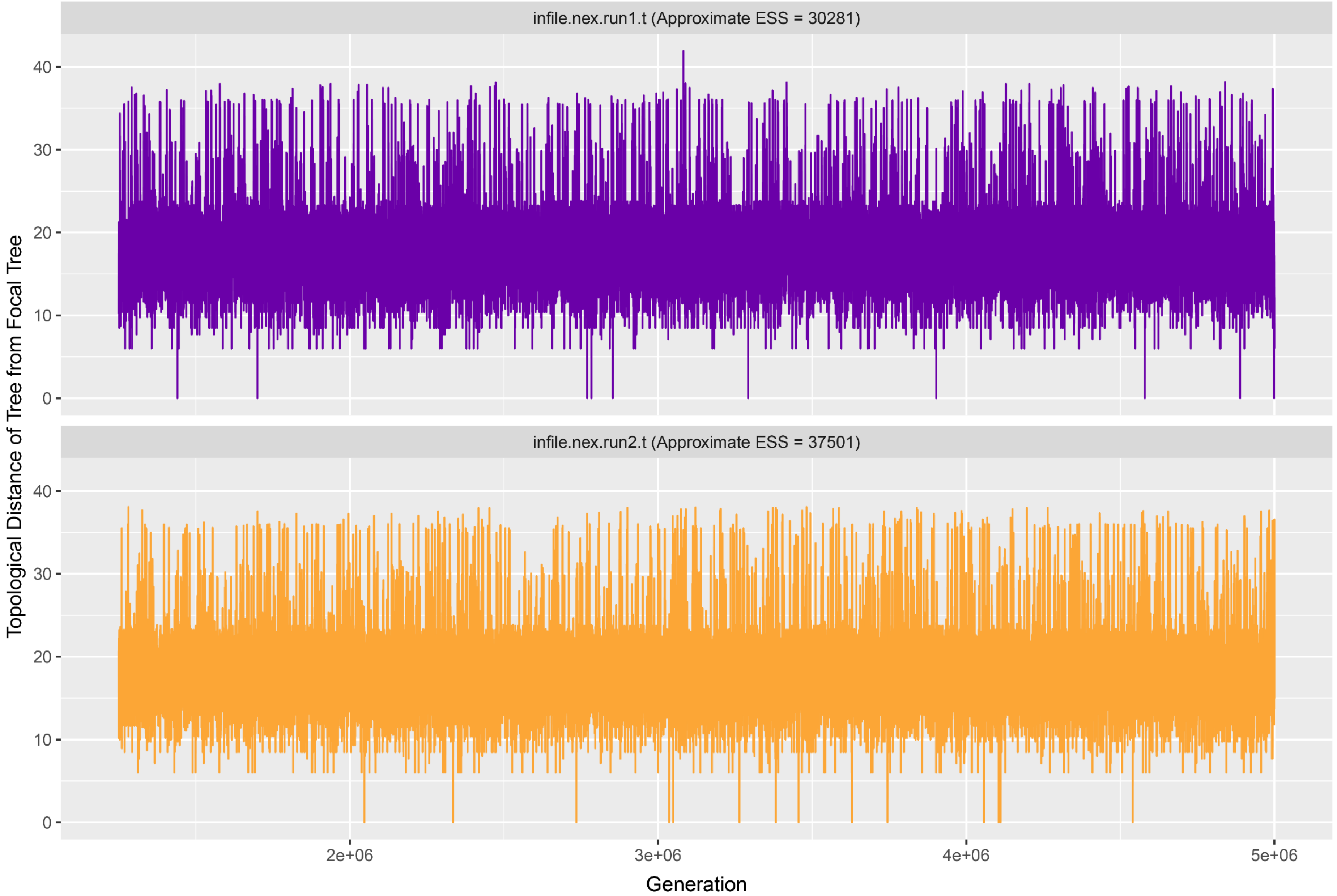

Figure S10. Tree topology trace plots of two runs of the MrBayes MCMC analyses of the unmasked 18S rRNA gene + ITS region dataset

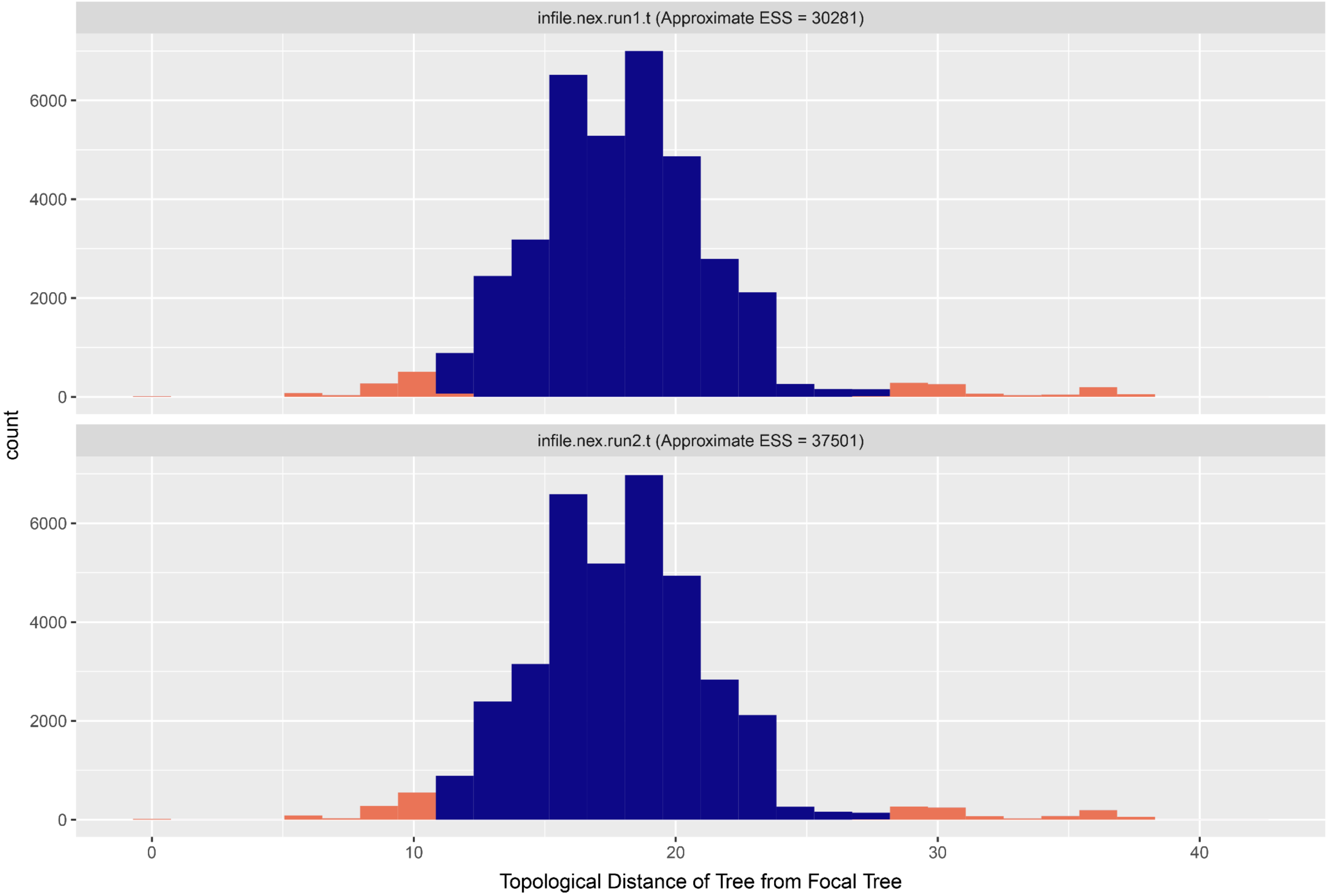

Figure S11. Density plots of tree topology trace of two runs of the MrBayes MCMC analyses of the unmasked 18S rRNA gene + ITS region dataset. Red values indicate values outside the 95% credibility intervals

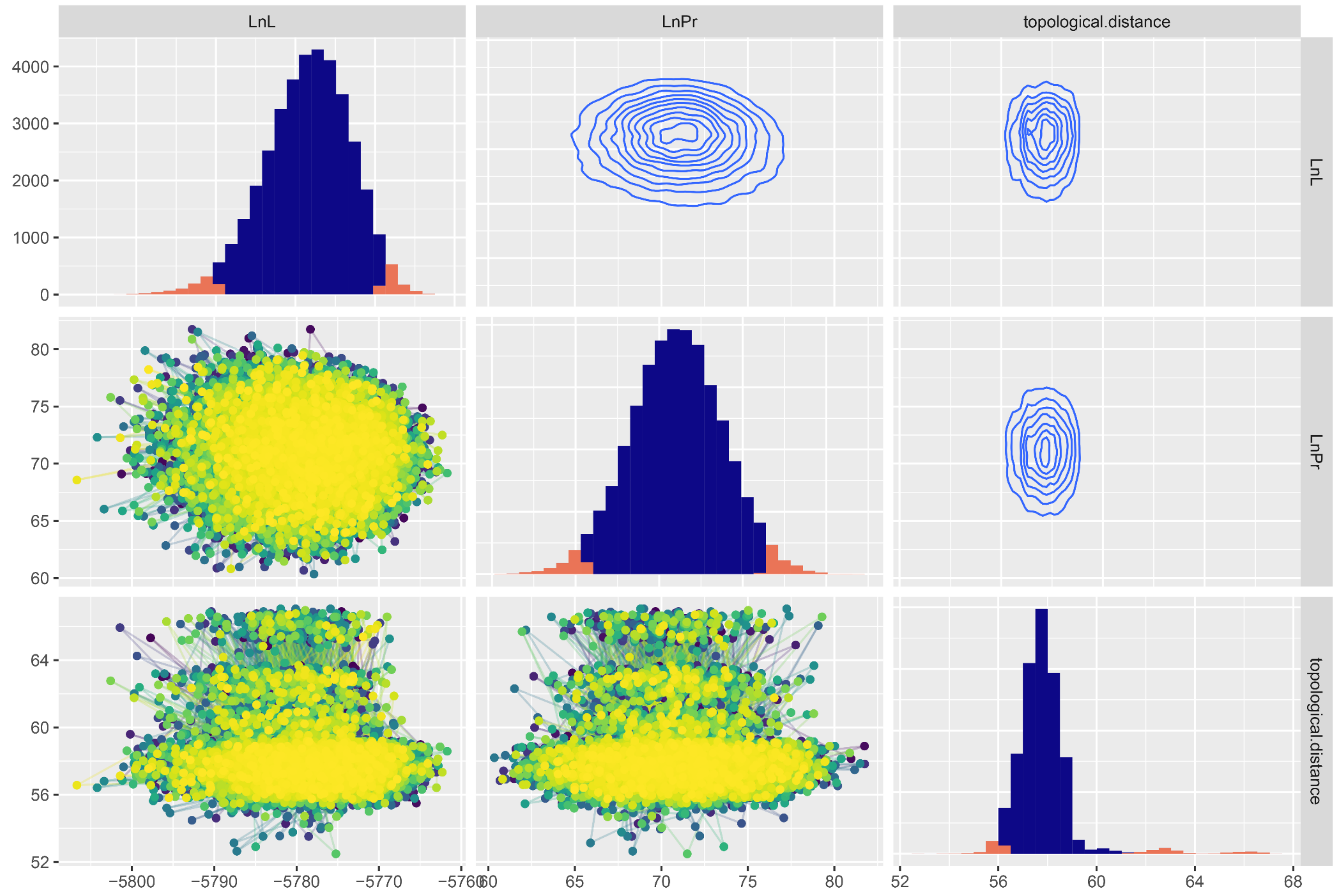

Figure S12. Plots showing correlations between tree topology and continuous model parameters of the first run of the MrBayes MCMC analyses of the unmasked 18S rRNA gene + ITS region dataset

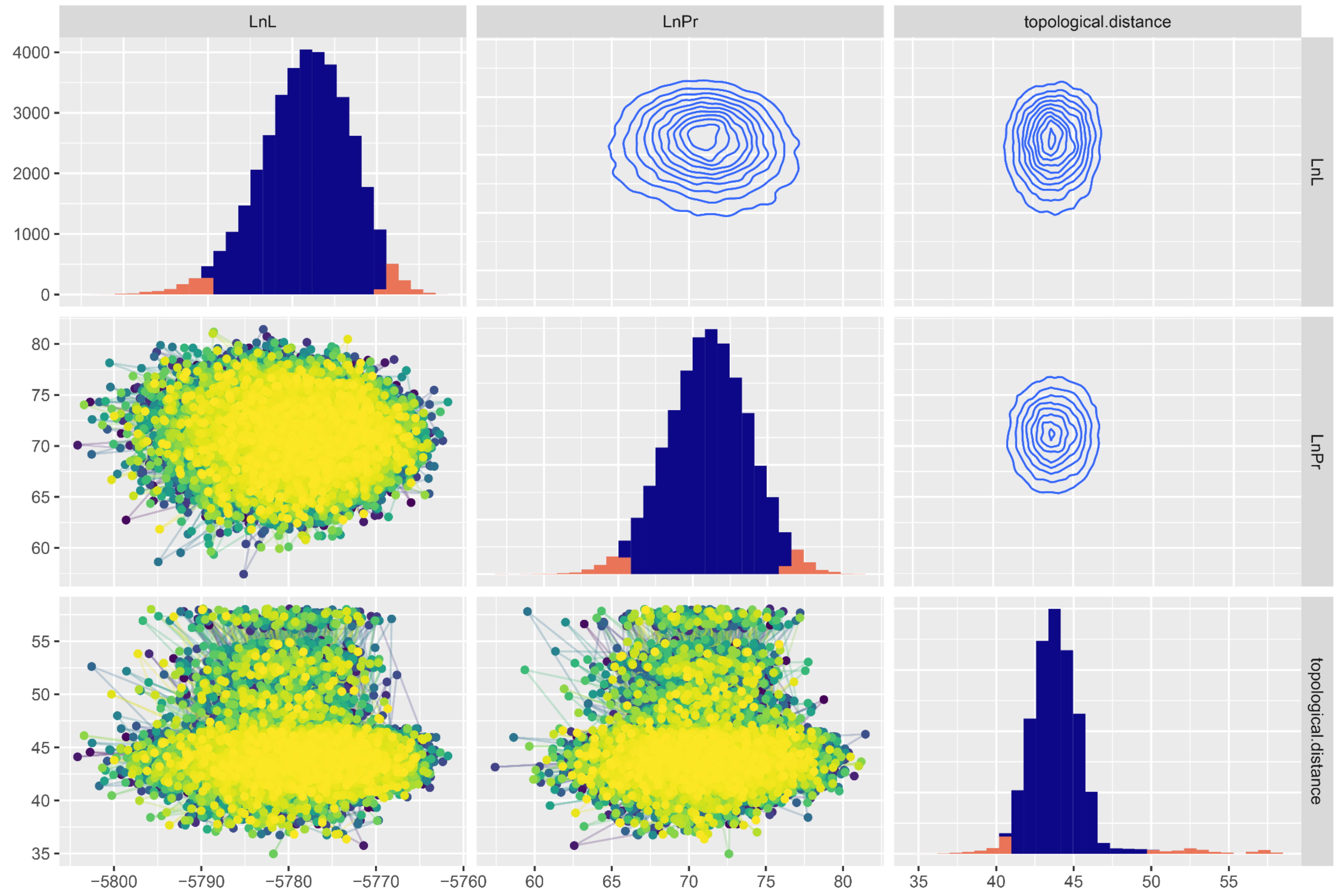

Figure S13. Plots showing correlations between tree topology and continuous model parameters of the second run of the MrBayes MCMC analyses of the unmasked 18S rRNA gene + ITS region dataset

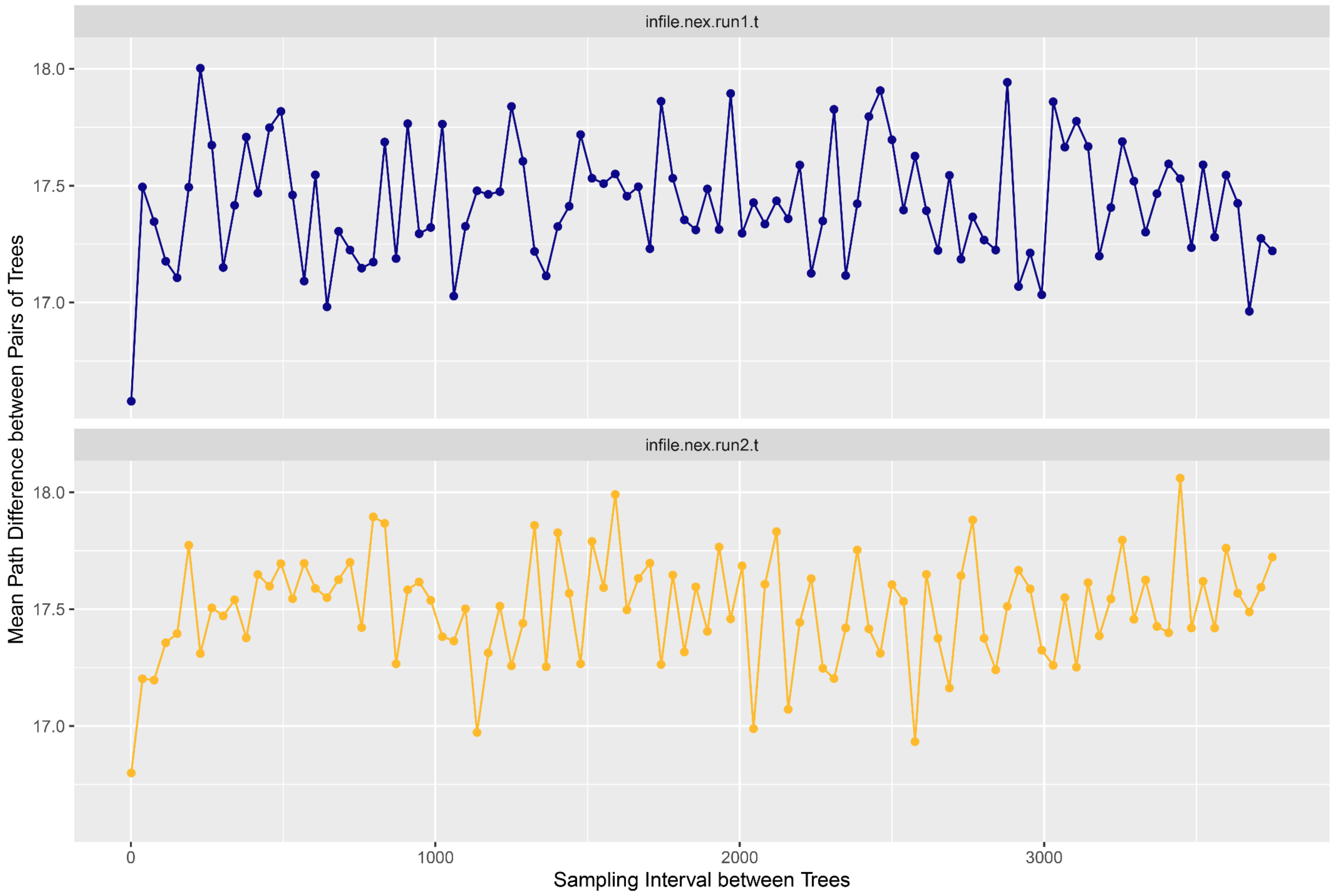

Figure S14. Topological autocorrelation plots of two runs of the MrBayes MCMC analyses of the unmasked 18S rRNA gene + ITS region dataset

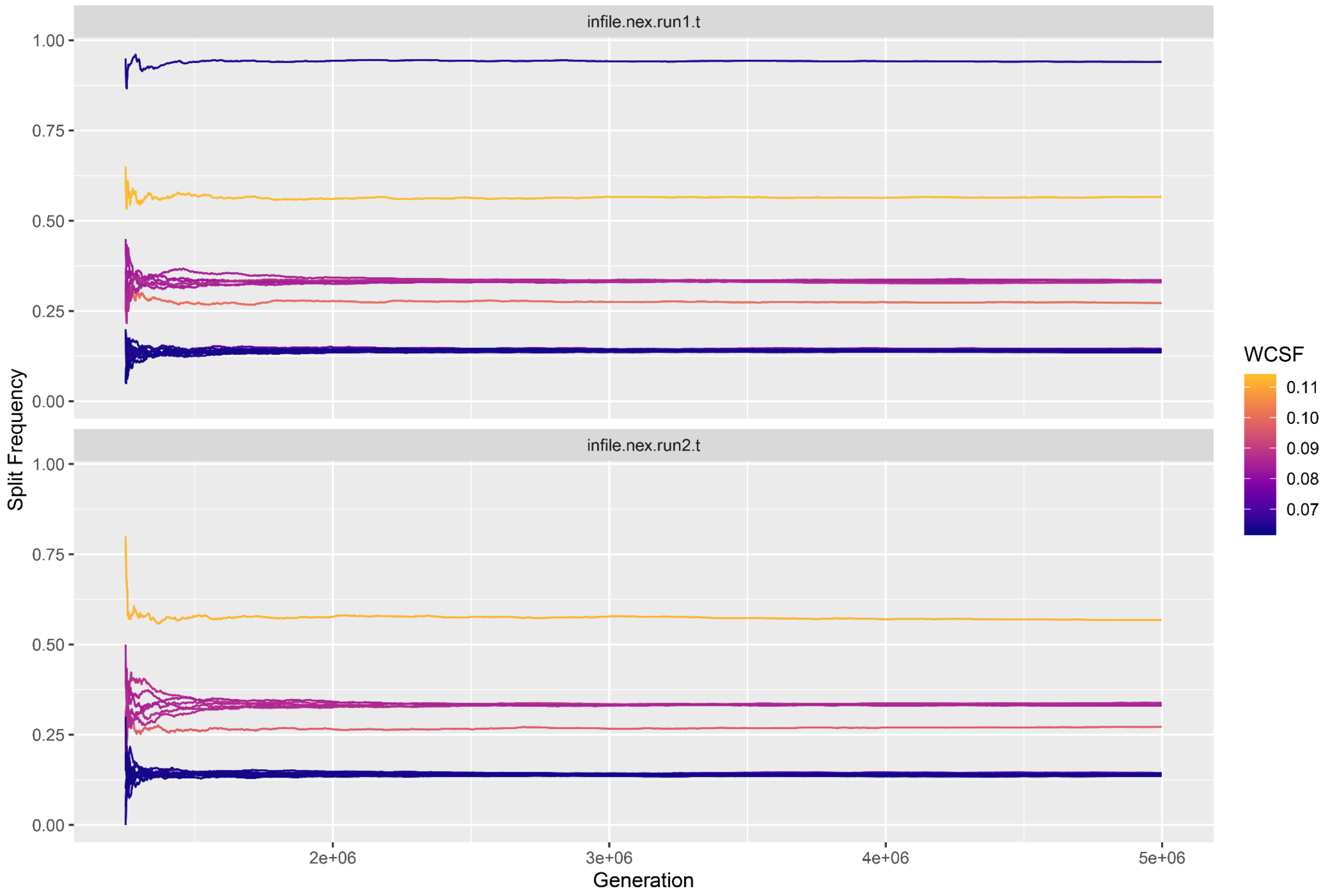

Figure S15. Plots showing cumulative split frequencies for 20 most variable clades of two runs of the MrBayes MCMC analyses of the unmasked 18S rRNA gene + ITS region dataset

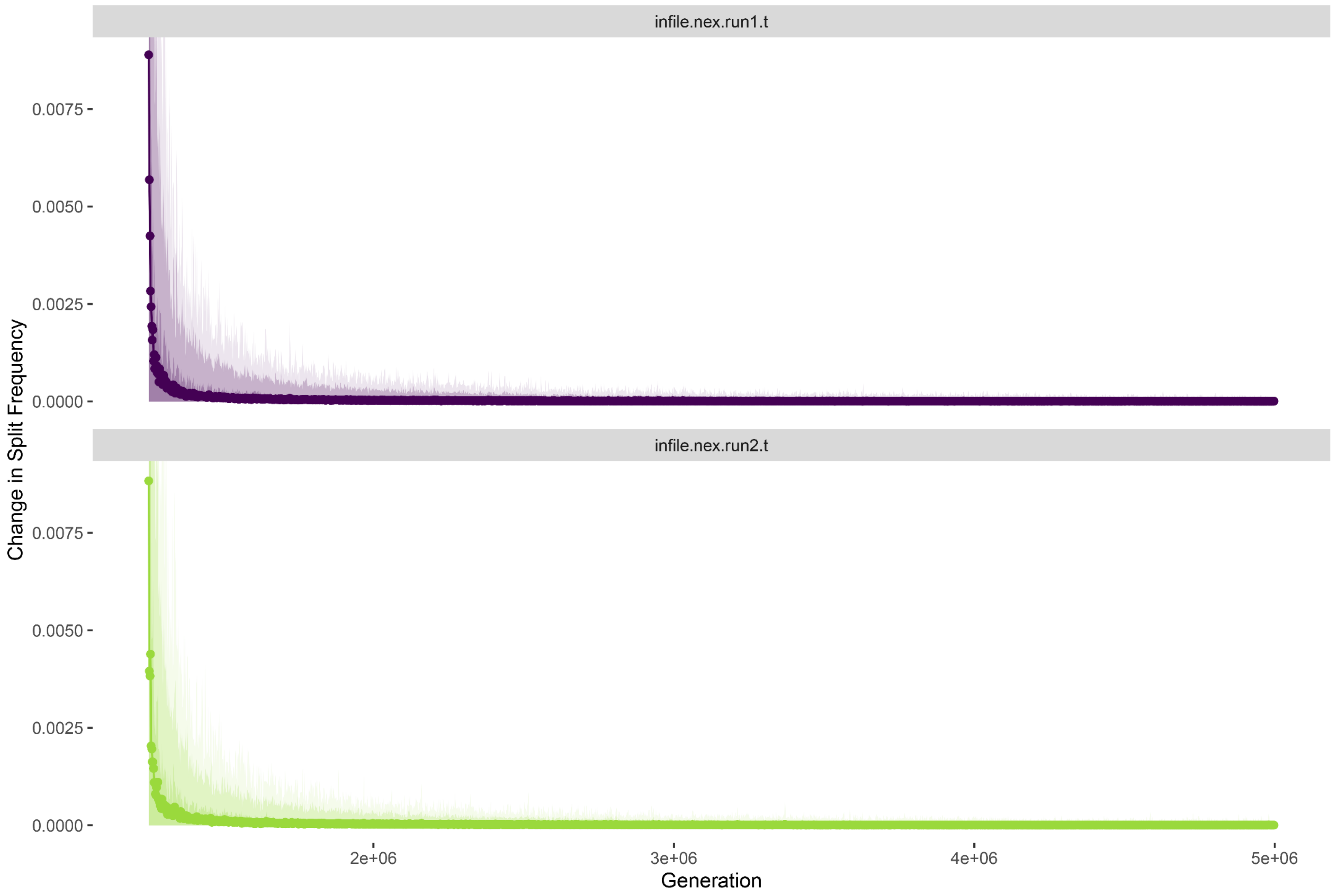

Figure S16. Plots showing cumulative change in split frequencies of two runs of the MrBayes MCMC analyses of the unmasked 18S rRNA gene + ITS region dataset

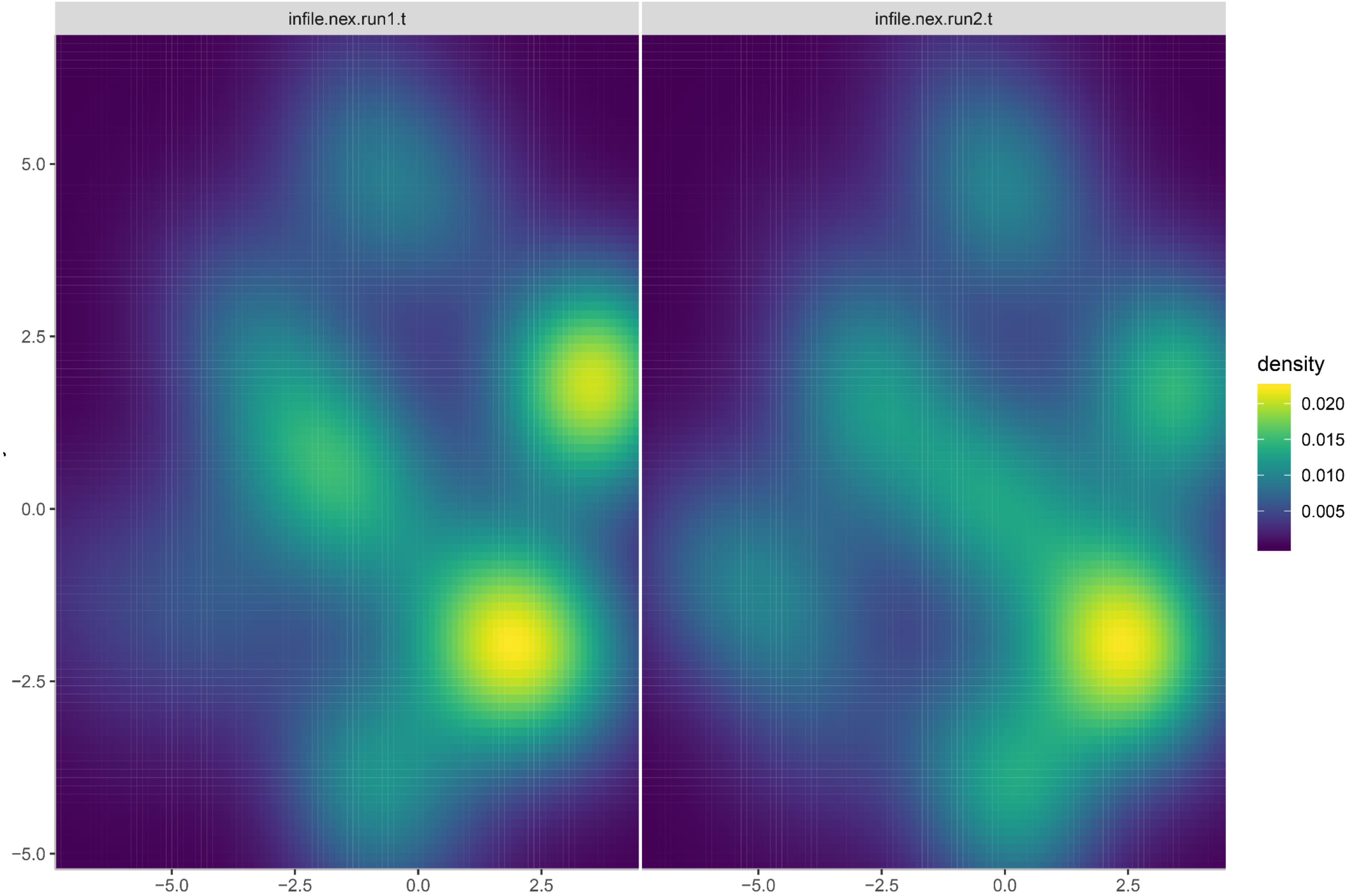

Figure S17. Tree space heatmaps for 100 trees of two runs of the MrBayes MCMC analyses of the unmasked 18S rRNA gene + ITS region dataset

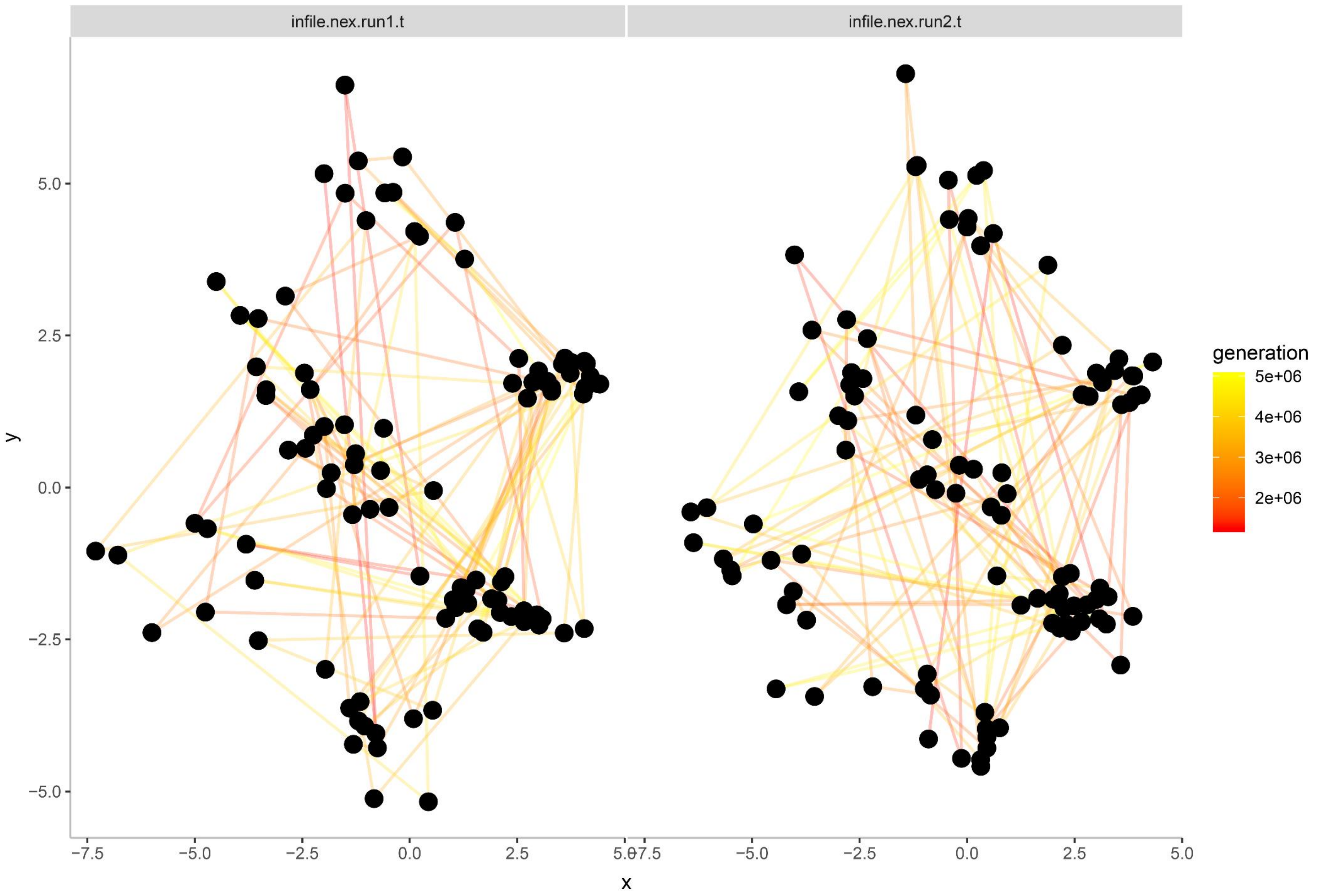

Figure S18. NMDS plots for 100 trees of two runs of the MrBayes MCMC analyses of the unmasked 18S rRNA gene + ITS region dataset
