# Supplementary material for: Delimitation of five astome ciliate species isolated from the digestive tube of three ecologically different groups of lumbricid earthworms, using the internal transcribed spacer region and the hypervariable D1/D2 region of the 28S rRNA gene
Source: BMC Evol Biol. 2020 Mar 14;20:37. doi: 10.1186/s12862-020-1601-2 (PMC7071660; doi:10.1186/s12862-020-1601-2)
Supplement: Supplementary file 11 — Additional file 11: Figures S32–S44. Results of RWTY analyses of Phycas MCMC runs of the unmasked 18S rRNA gene + ITS region dataset. [file 12862_2020_1601_MOESM11_ESM.pdf]

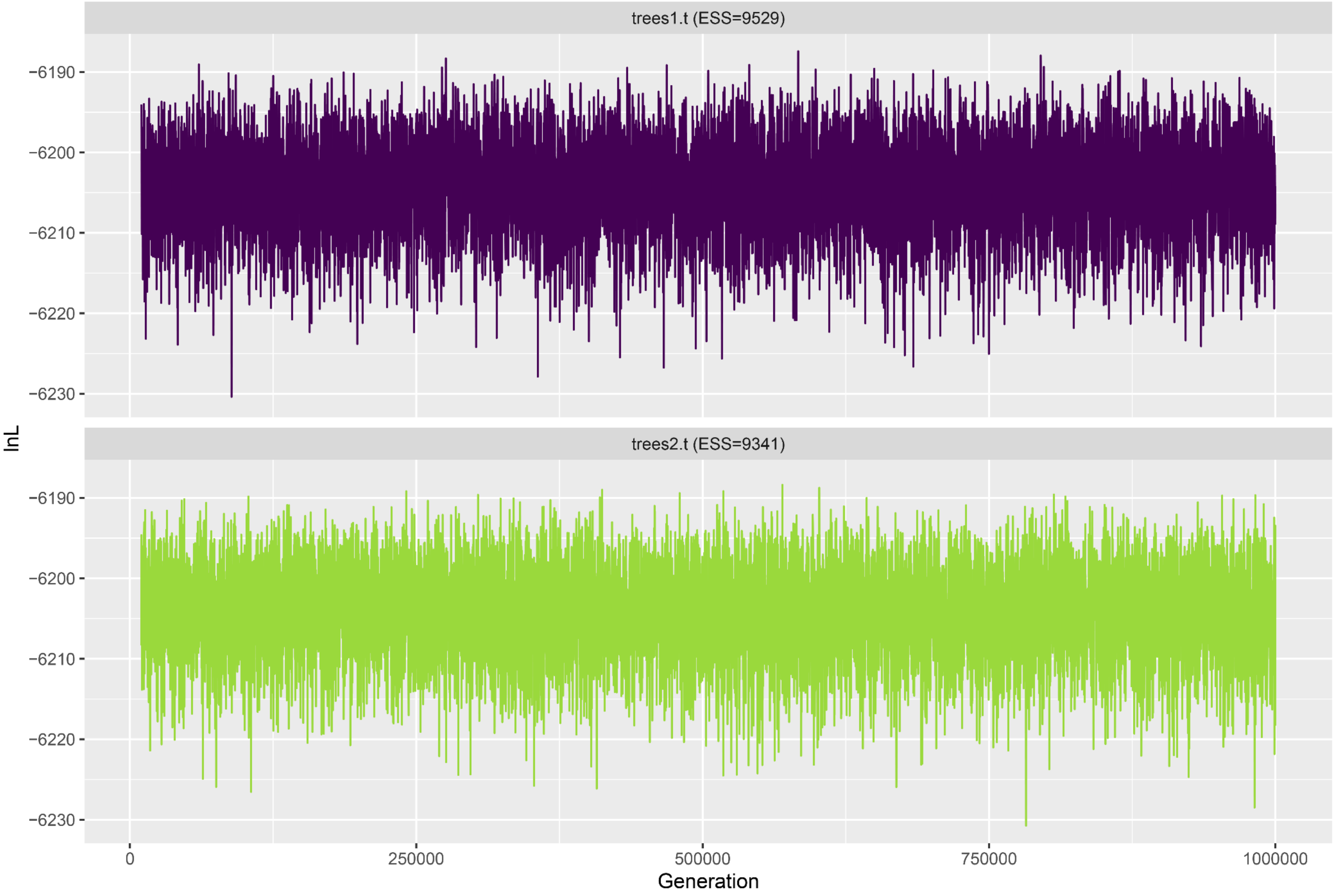

Figure S32. Trace plots of log likelihood (lnL) of phylogenetic trees of two runs of the Phycas MCMC analyses of the unmasked 18S rRNA gene + ITS region dataset

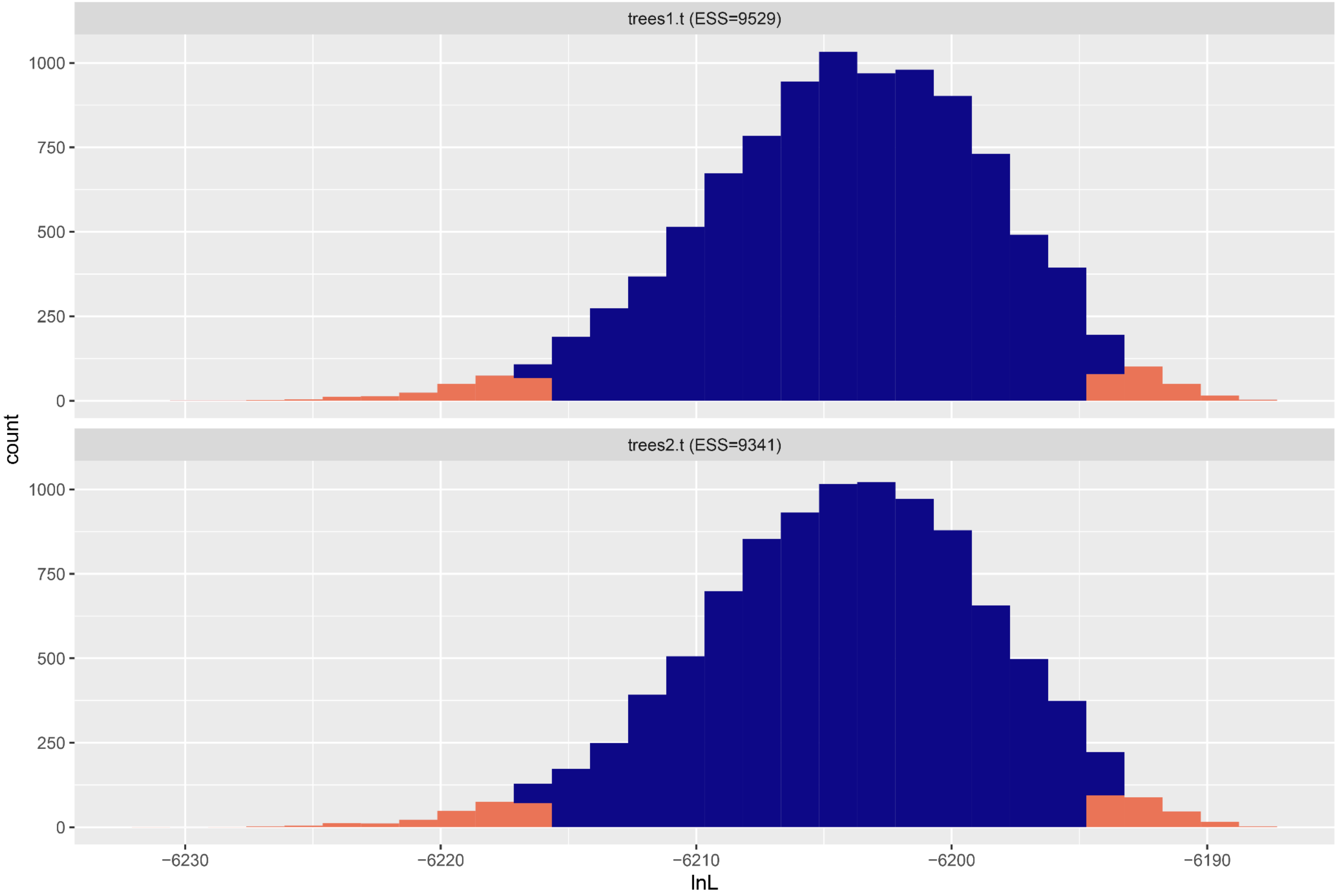

Figure S33. Density plots of log likelihood (lnL) of phylogenetic trees of two runs of the Phycas MCMC analyses of the unmasked 18S rRNA gene + ITS region dataset. Red values indicate values outside the 95% credibility intervals

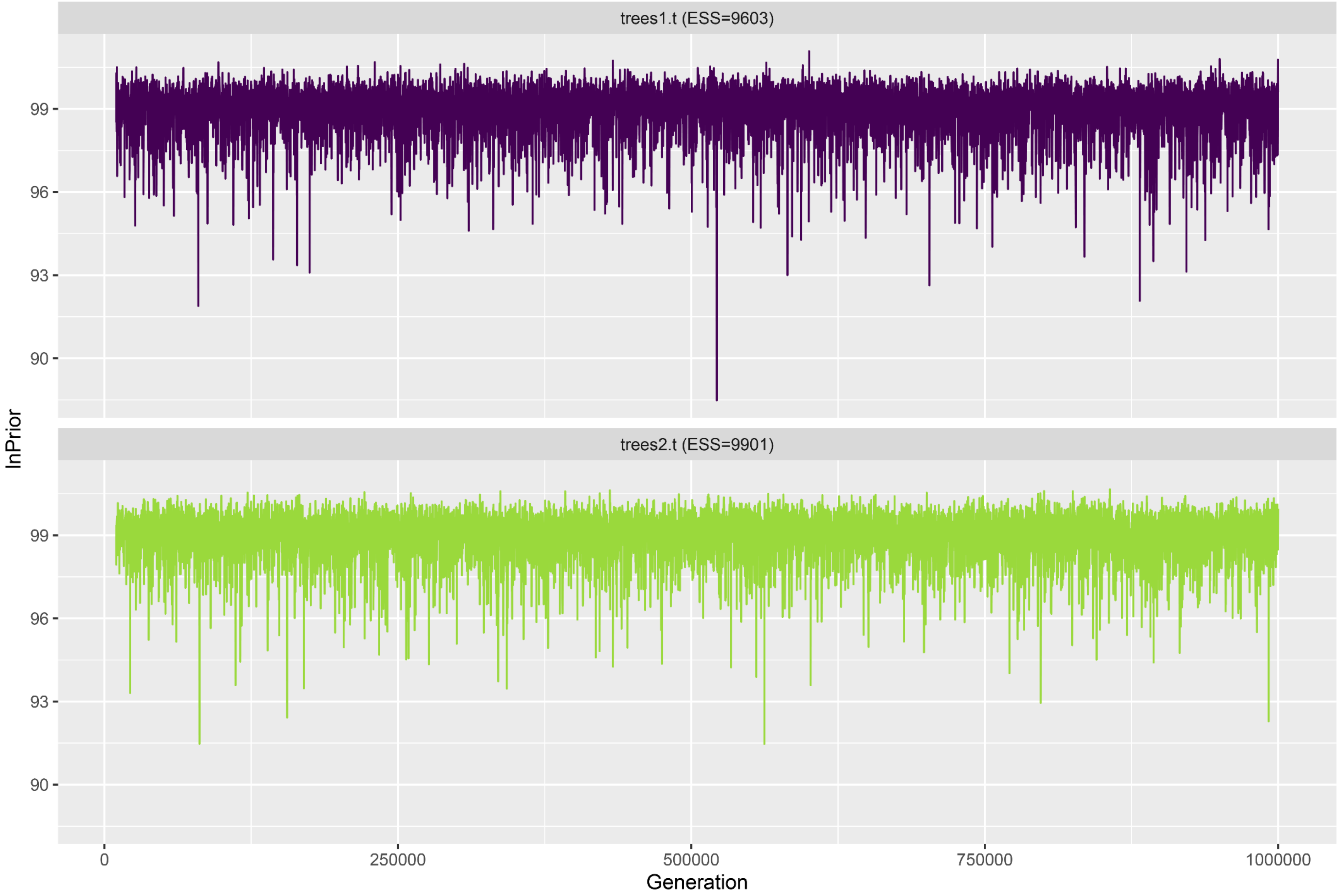

Figure S34. Trace plots of log prior parameters (lnPrior) of two runs of the Phycas MCMC analyses of the unmasked 18S rRNA gene + ITS region dataset

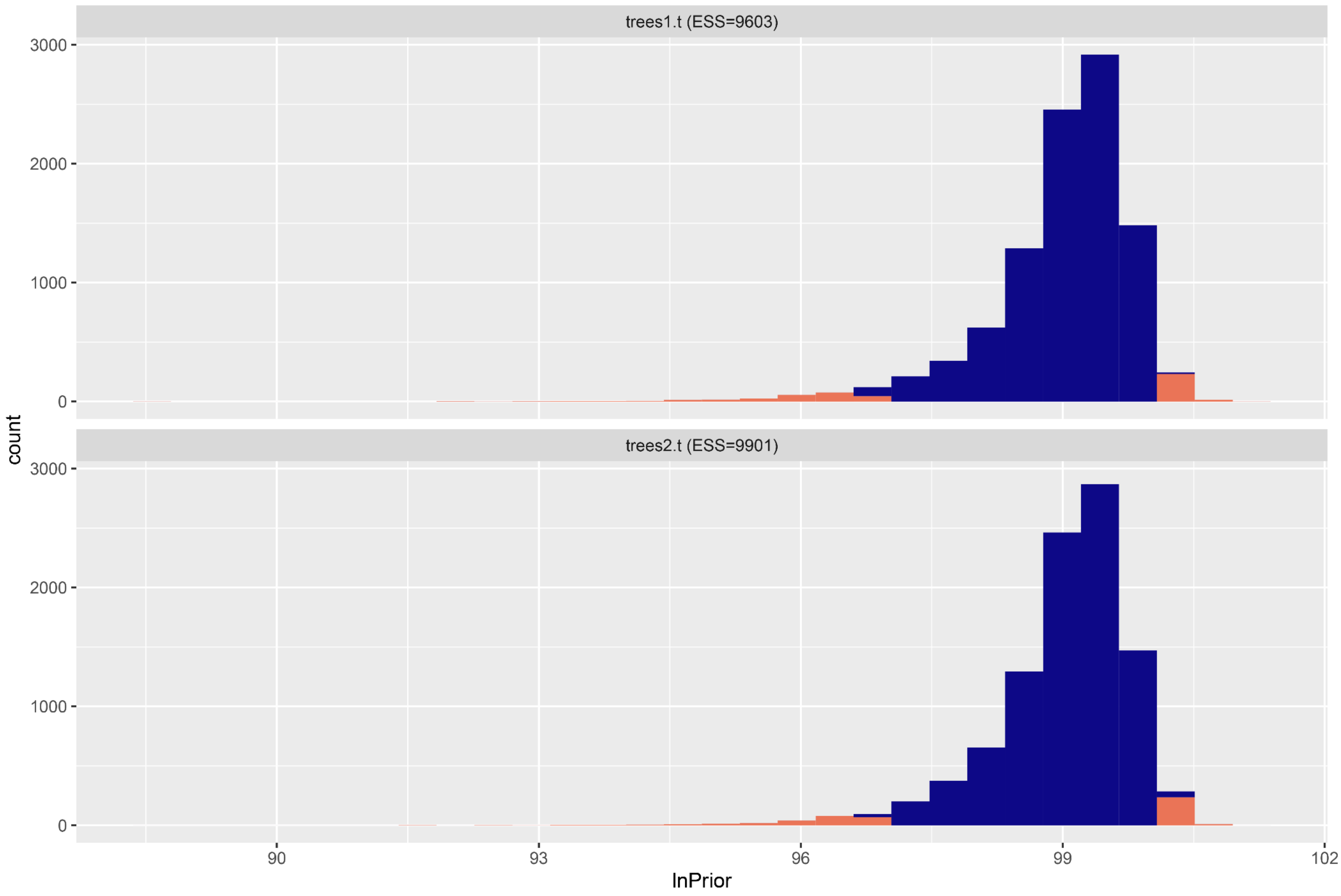

Figure S35. Density plots of log prior parameters (lnPrior) of two runs of the Phycas MCMC analyses of the unmasked 18S rRNA gene + ITS region dataset. Red values indicate values outside the 95% credibility intervals

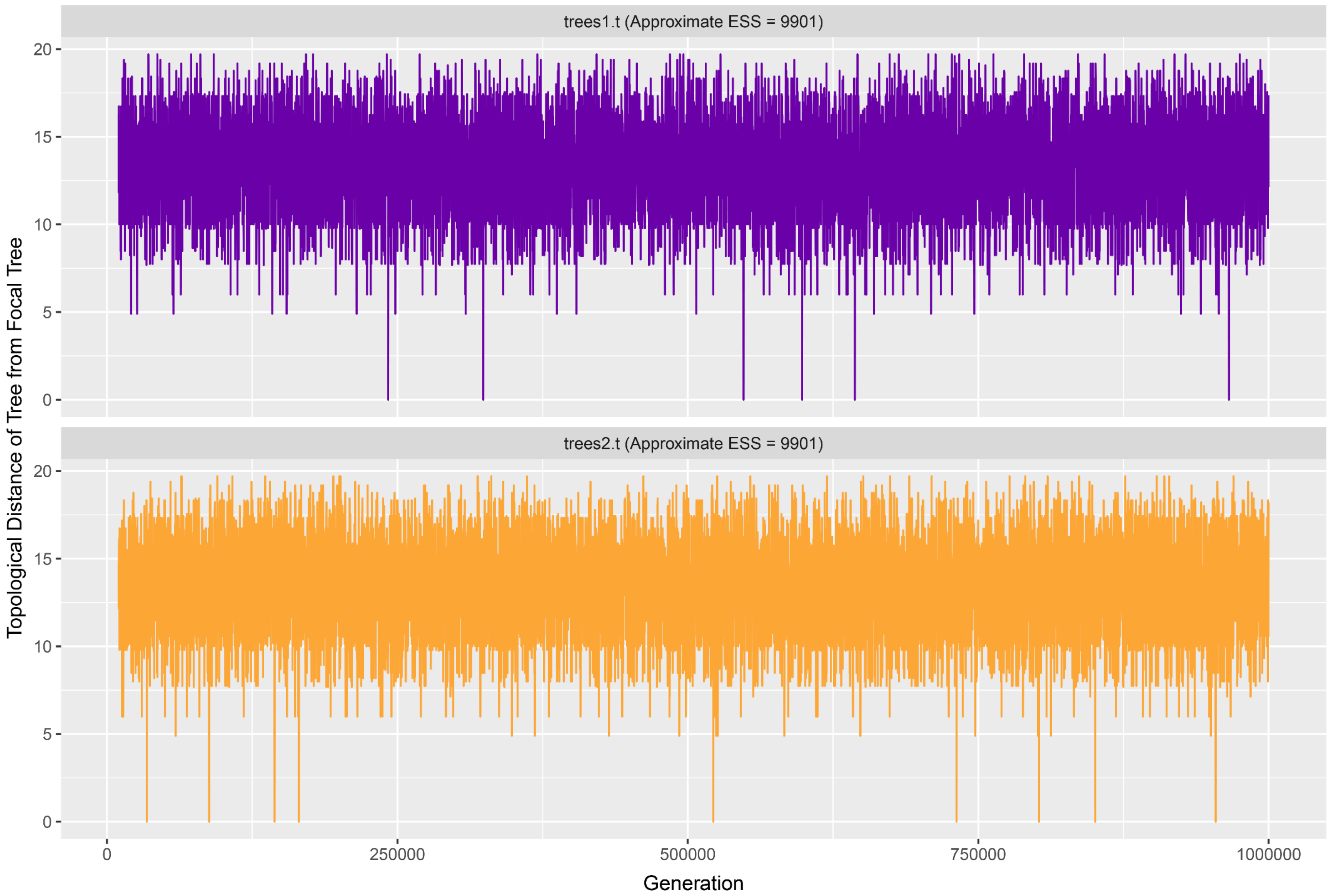

Figure S36. Tree topology trace plots of two runs of the Phycas MCMC analyses of the unmasked 18S rRNA gene + ITS region dataset

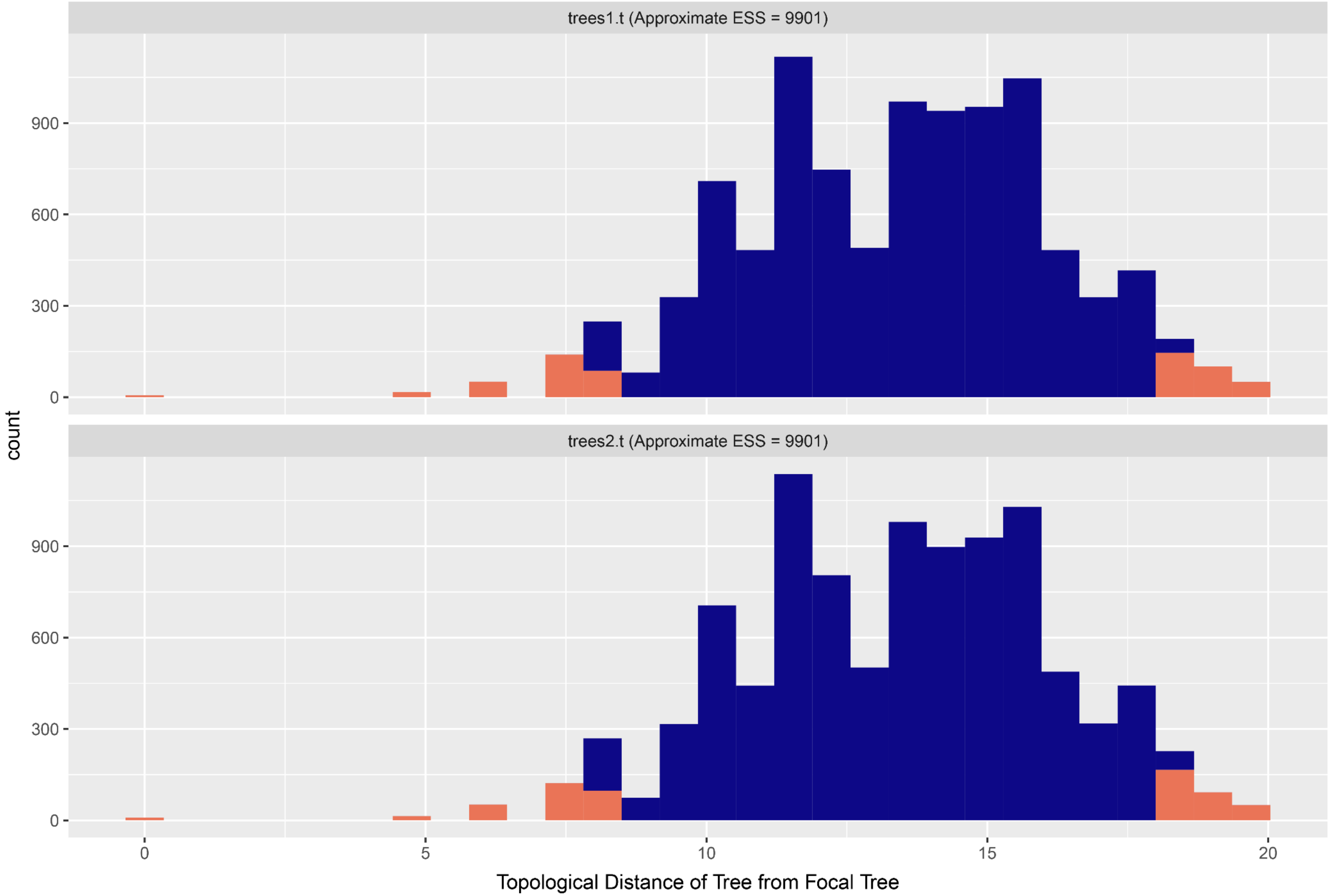

Figure S37. Density plots of tree topology trace of two runs of the Phycas MCMC analyses of the unmasked 18S rRNA gene + ITS region dataset. Red values indicate values outside the 95% credibility intervals

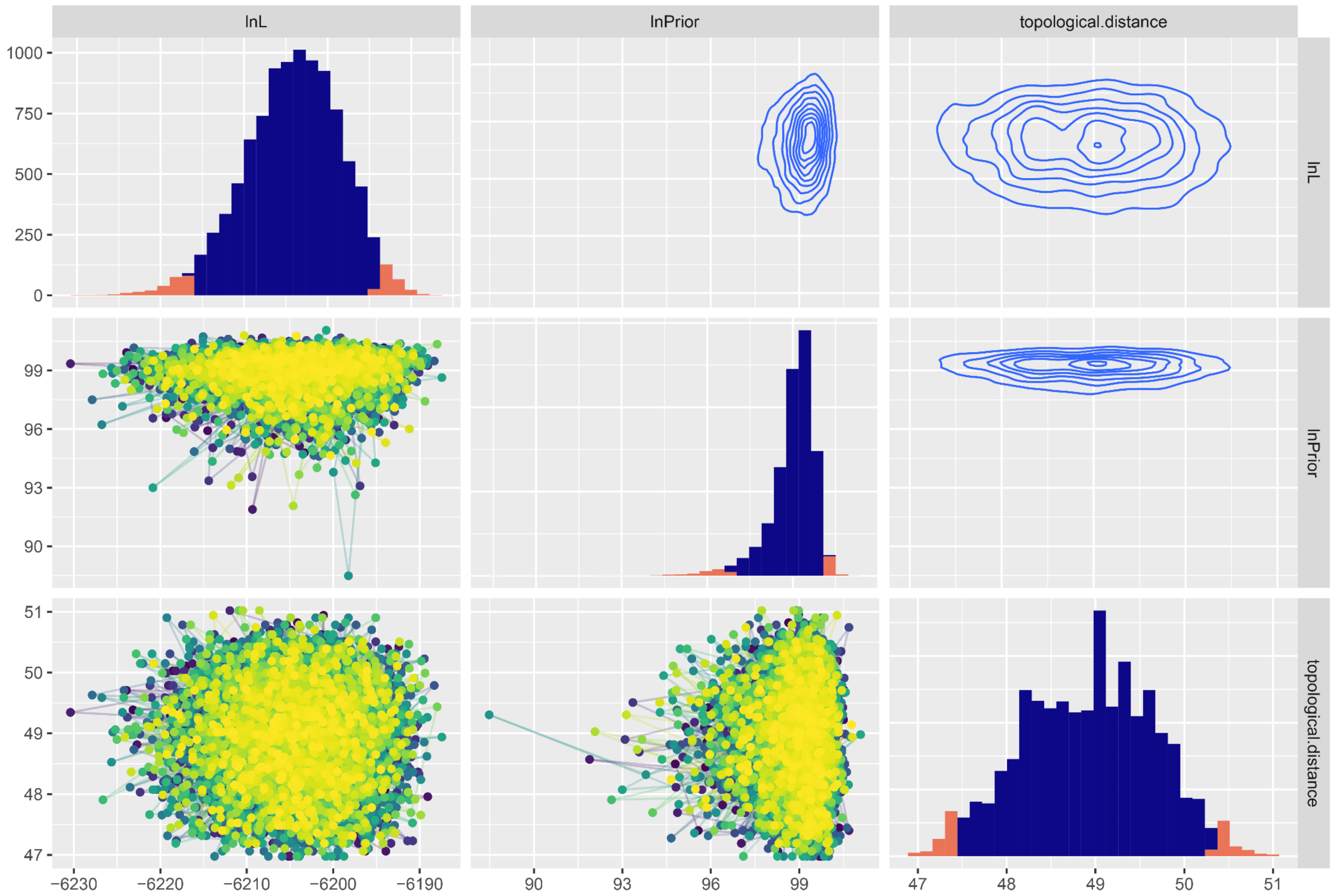

Figure S38. Plots showing correlations between tree topology and continuous model parameters of the first run of the Phycas MCMC analyses of the unmasked 18S rRNA gene + ITS region dataset

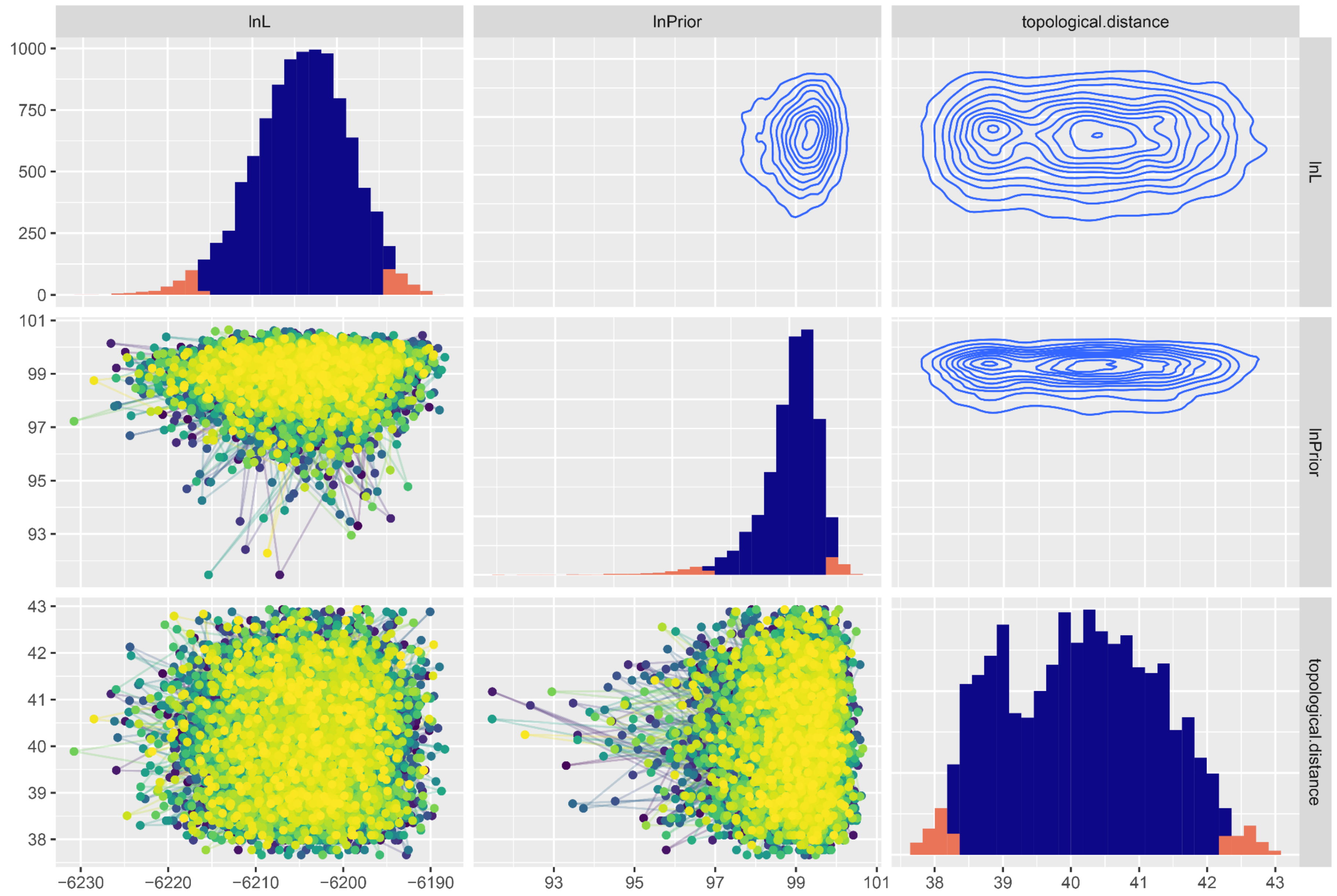

Figure S39. Plots showing correlations between tree topology and continuous model parameters of the second run of the Phycas MCMC analyses of the unmasked 18S rRNA gene + ITS region dataset

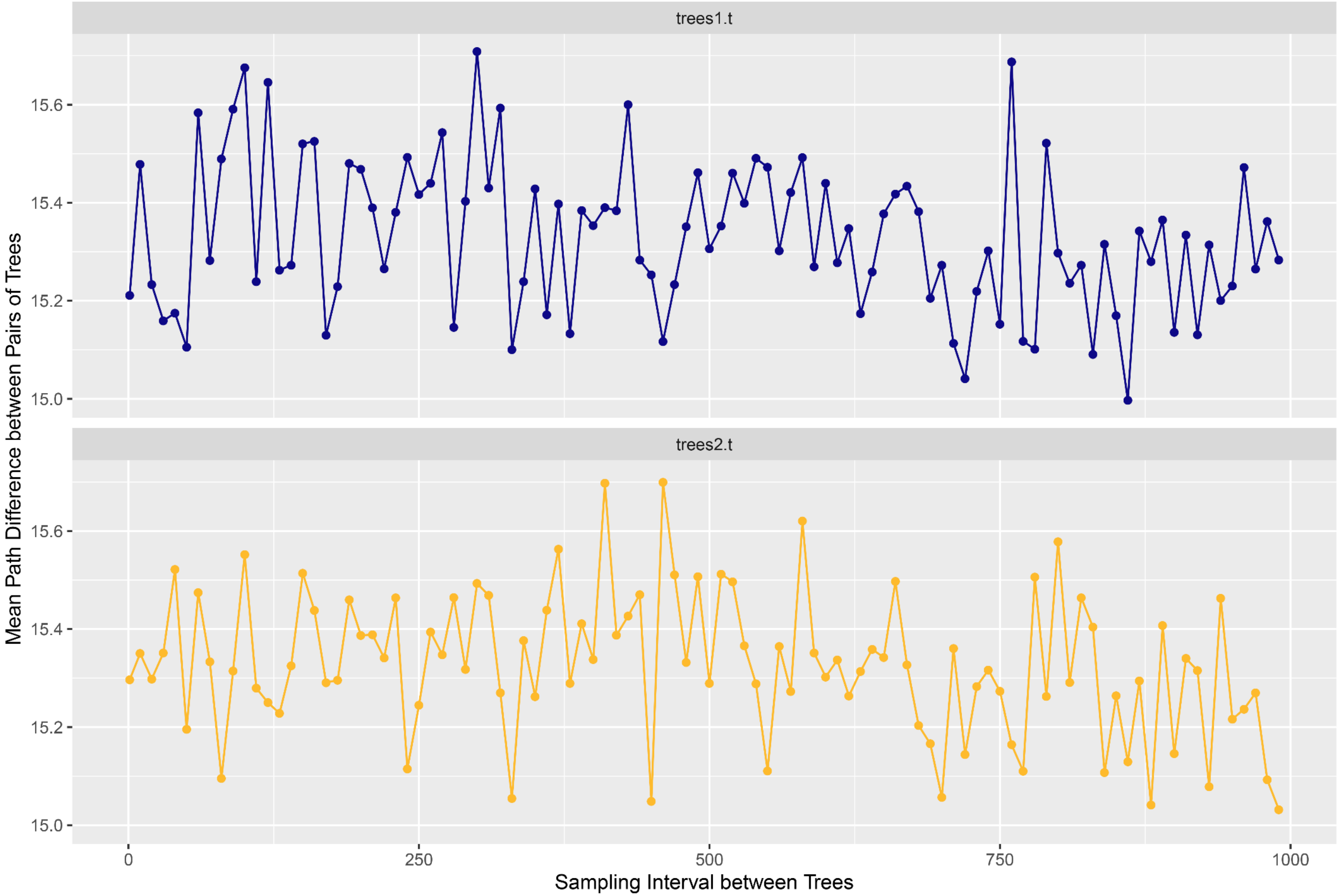

Figure S40. Topological autocorrelation plots of two runs of the Phycas MCMC analyses of the unmasked 18S rRNA gene + ITS region dataset

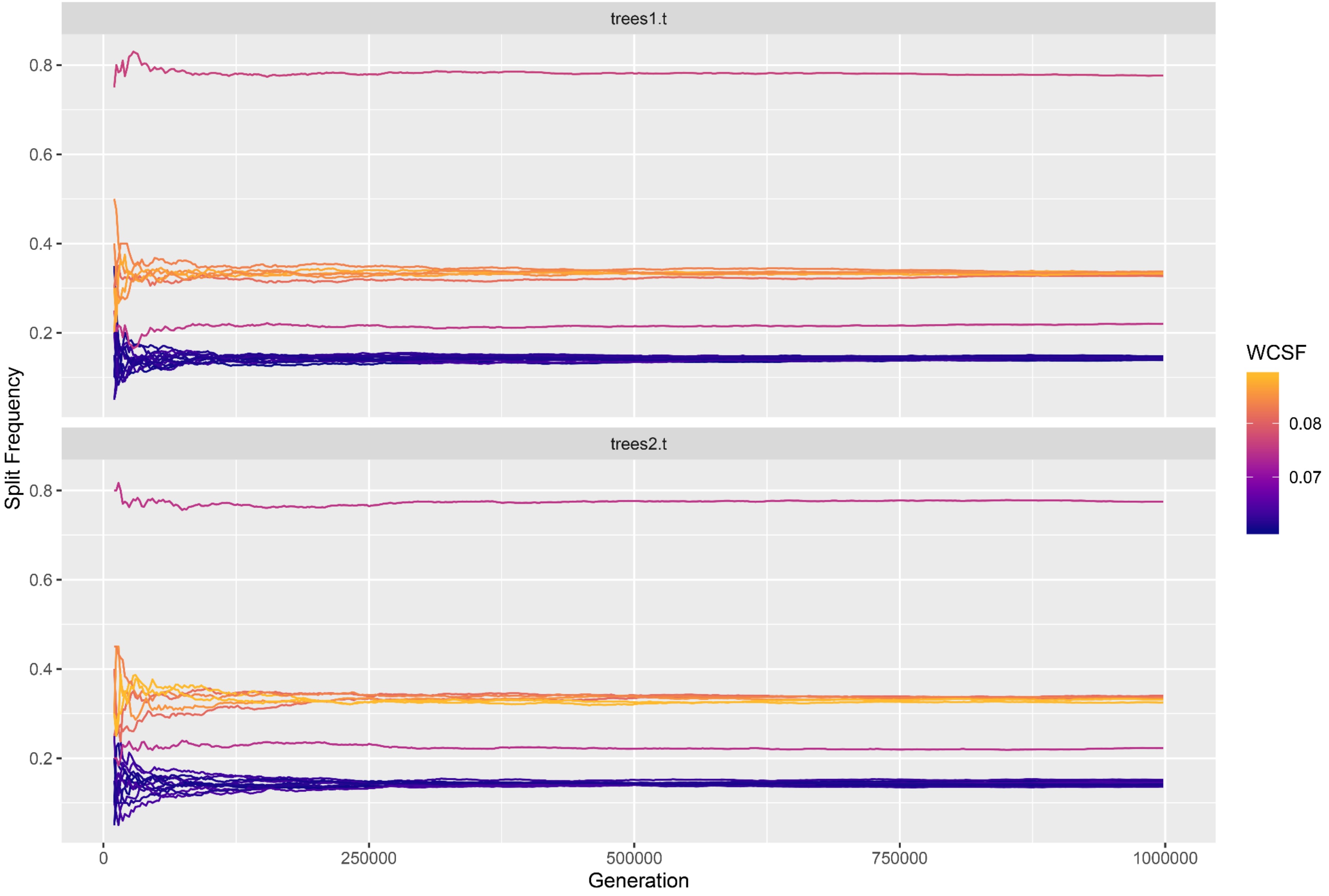

Figure S41. Plots showing cumulative split frequencies for 20 most variable clades of two runs of the Phycas MCMC analyses of the unmasked 18S rRNA gene + ITS region dataset

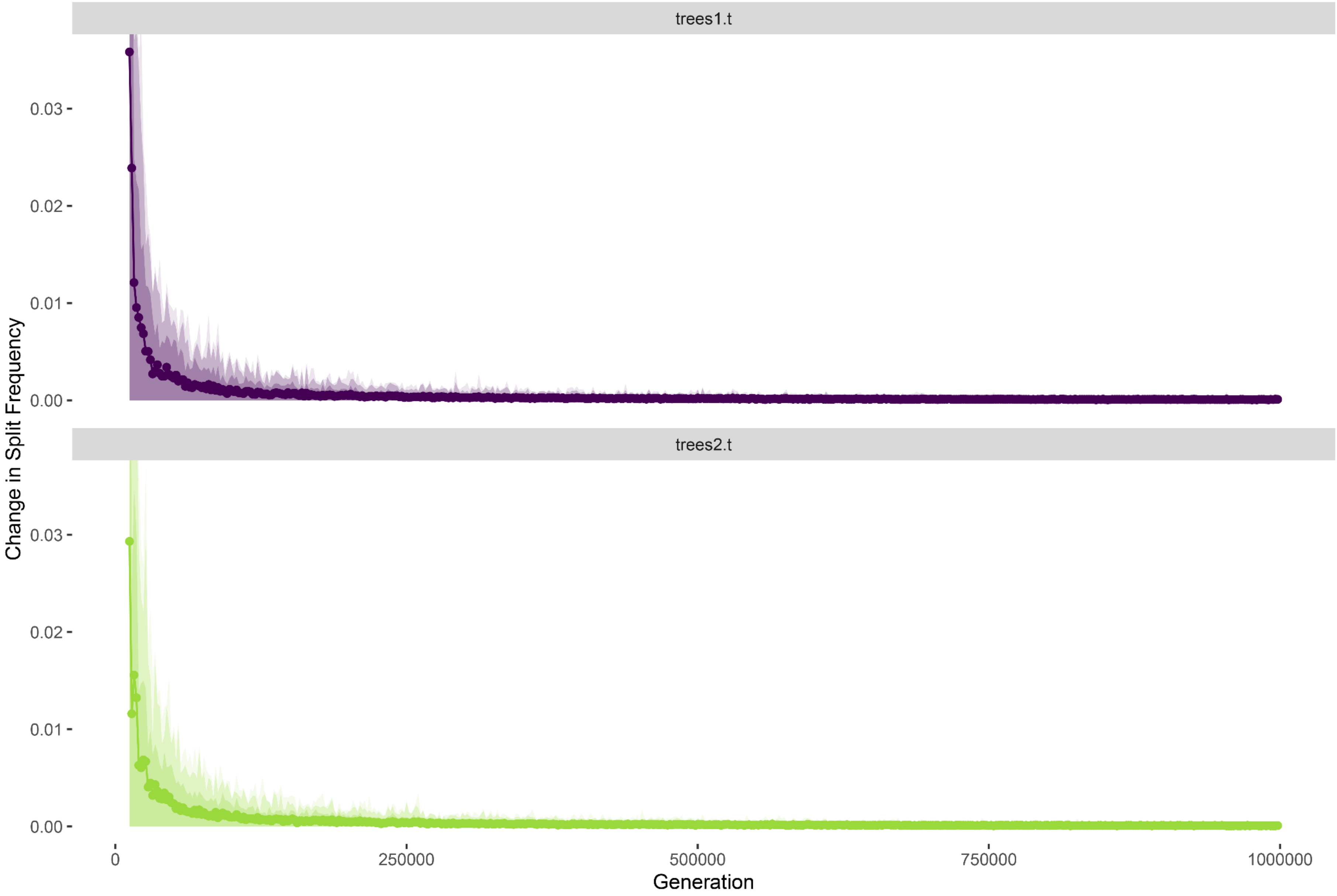

Figure S42. Plots showing cumulative change in split frequencies of two runs of the Phycas MCMC analyses of the unmasked 18S rRNA gene + ITS region dataset

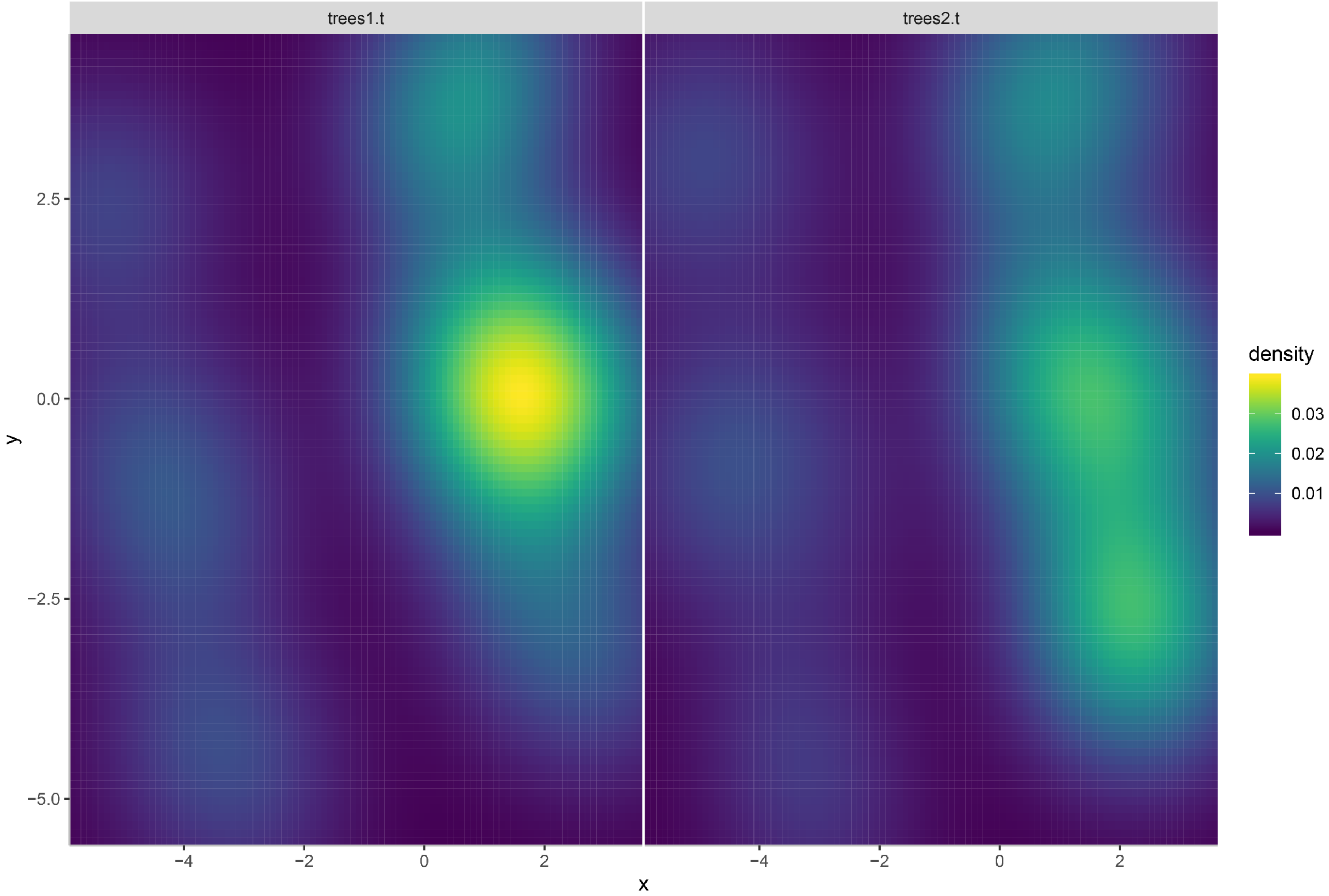

Figure S43. Tree space heatmaps for 100 trees of two runs of the Phycas MCMC analyses of the unmasked 18S rRNA gene + ITS region dataset

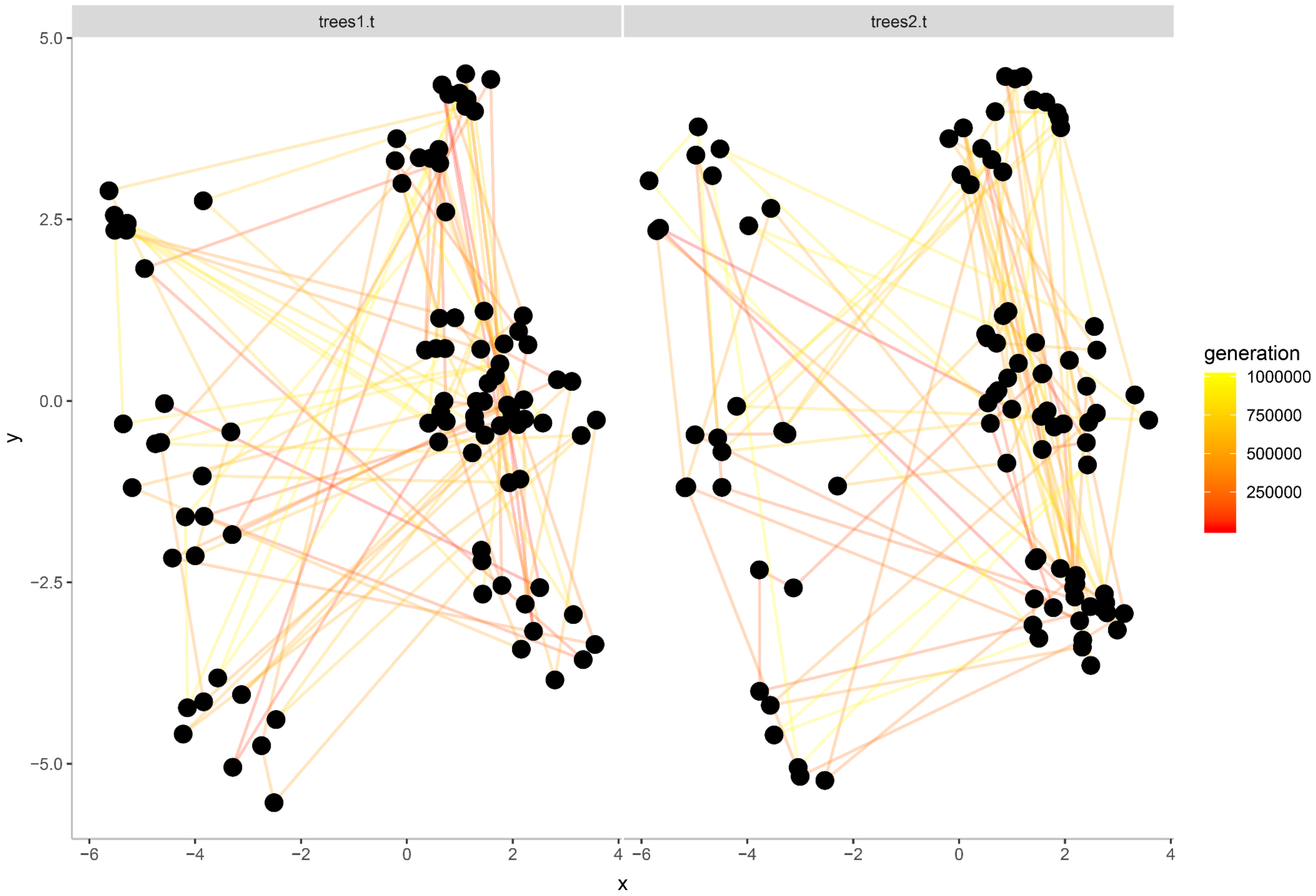

Figure S44. NMDS plots for 100 trees of two runs of the Phycas MCMC analyses of the unmasked 18S rRNA gene + ITS region dataset
