# Supplementary material for: Delimitation of five astome ciliate species isolated from the digestive tube of three ecologically different groups of lumbricid earthworms, using the internal transcribed spacer region and the hypervariable D1/D2 region of the 28S rRNA gene
Source: BMC Evol Biol. 2020 Mar 14;20:37. doi: 10.1186/s12862-020-1601-2 (PMC7071660; doi:10.1186/s12862-020-1601-2)
Supplement: Supplementary file 12 — Additional file 12: Figures S45–S57. Results of RWTY analyses of Phycas MCMC runs of the 18S + 5.8S +28S rRNA gene dataset masked with a cut-off value of 0.93. [file 12862_2020_1601_MOESM12_ESM.pdf]

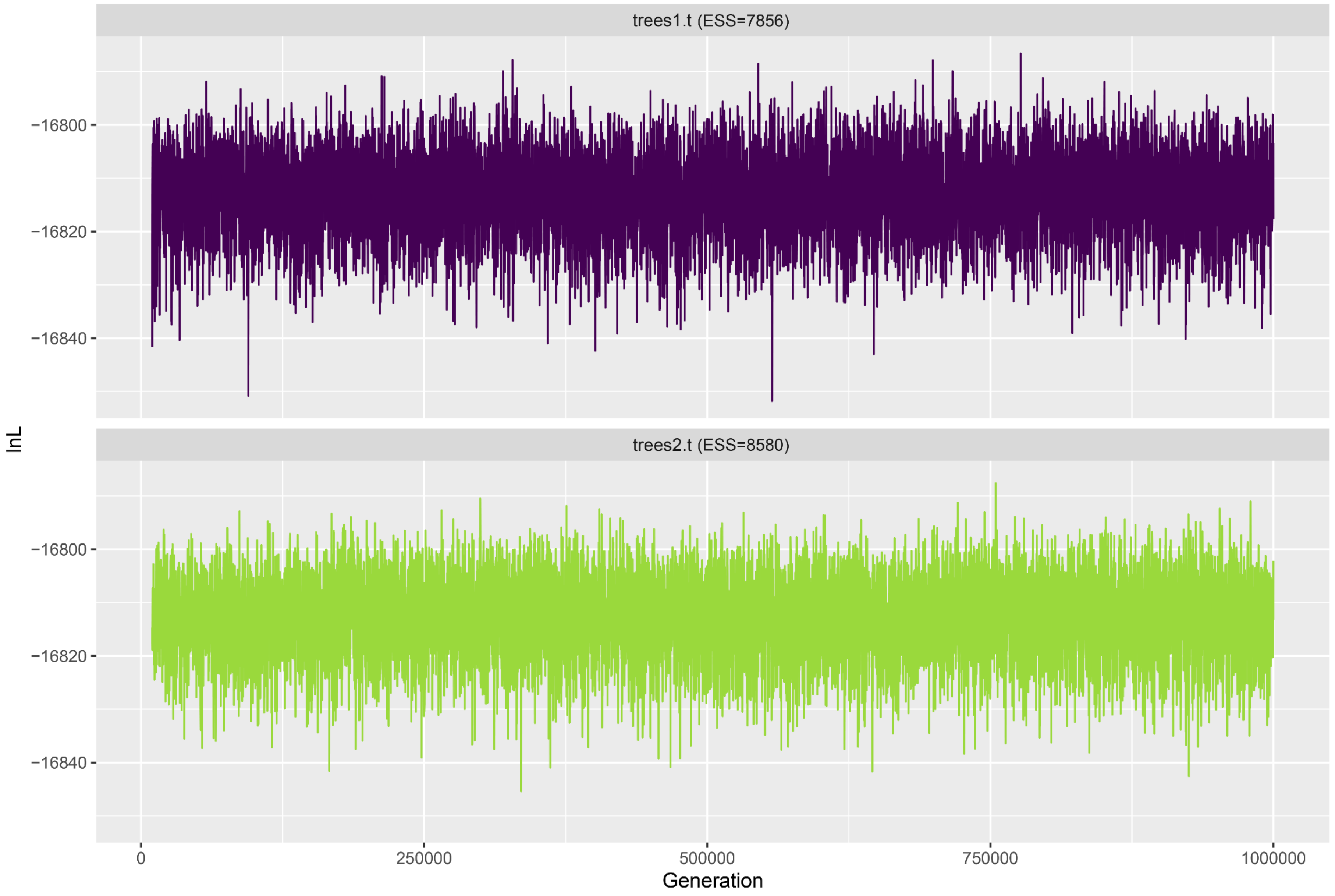

Figure S45. Trace plots of log likelihood (lnL) of phylogenetic trees of two runs of the Phycas MCMC analyses of the 18S + 5.8S +28S rRNA gene dataset masked with a cut-off value of 0.93

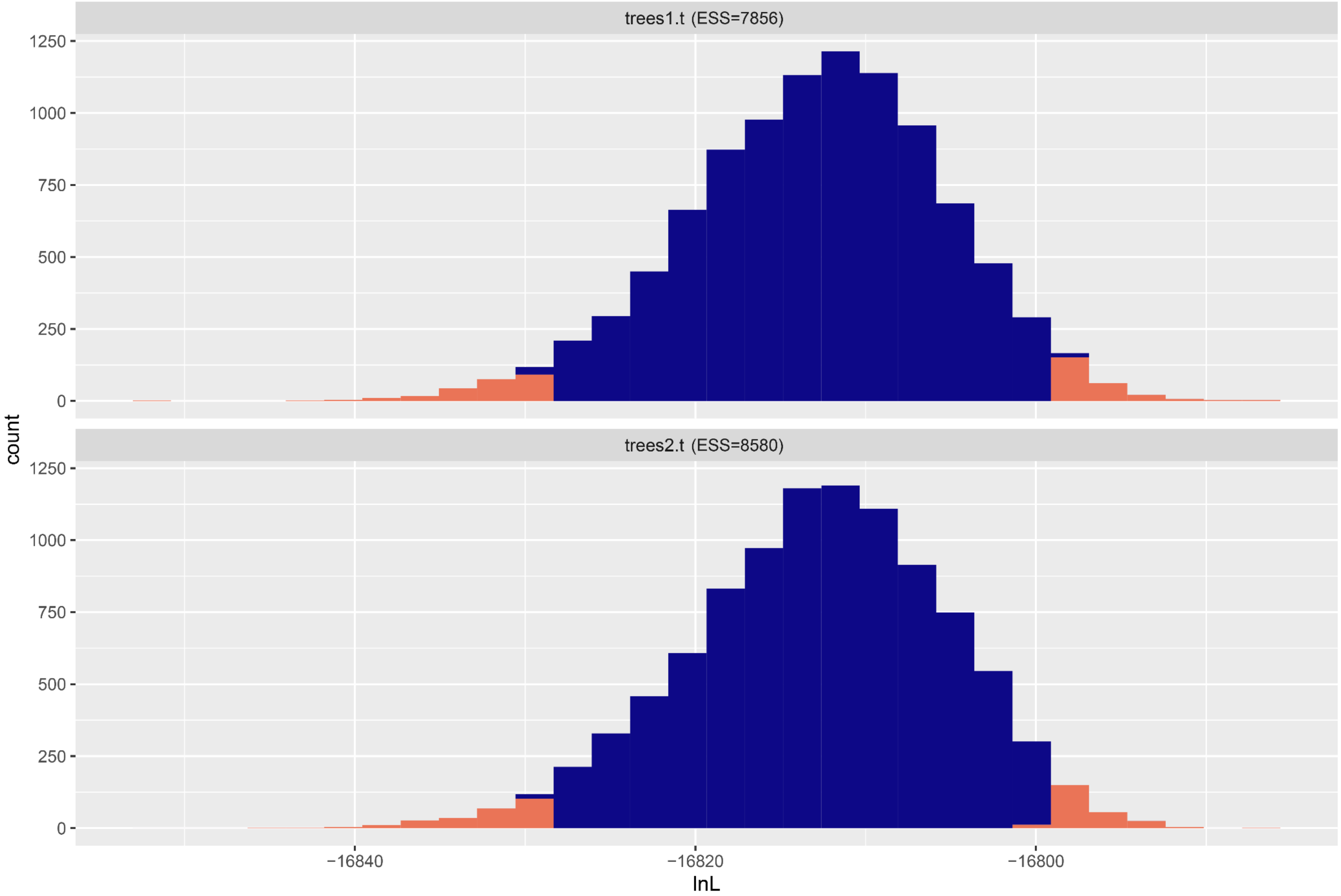

Figure S46. Density plots of log likelihood (lnL) of phylogenetic trees of two runs of the Phycas MCMC analyses of the 18S + 5.8S +28S rRNA gene dataset masked with a cut-off value of 0.93. Red values indicate values outside the 95% credibility intervals

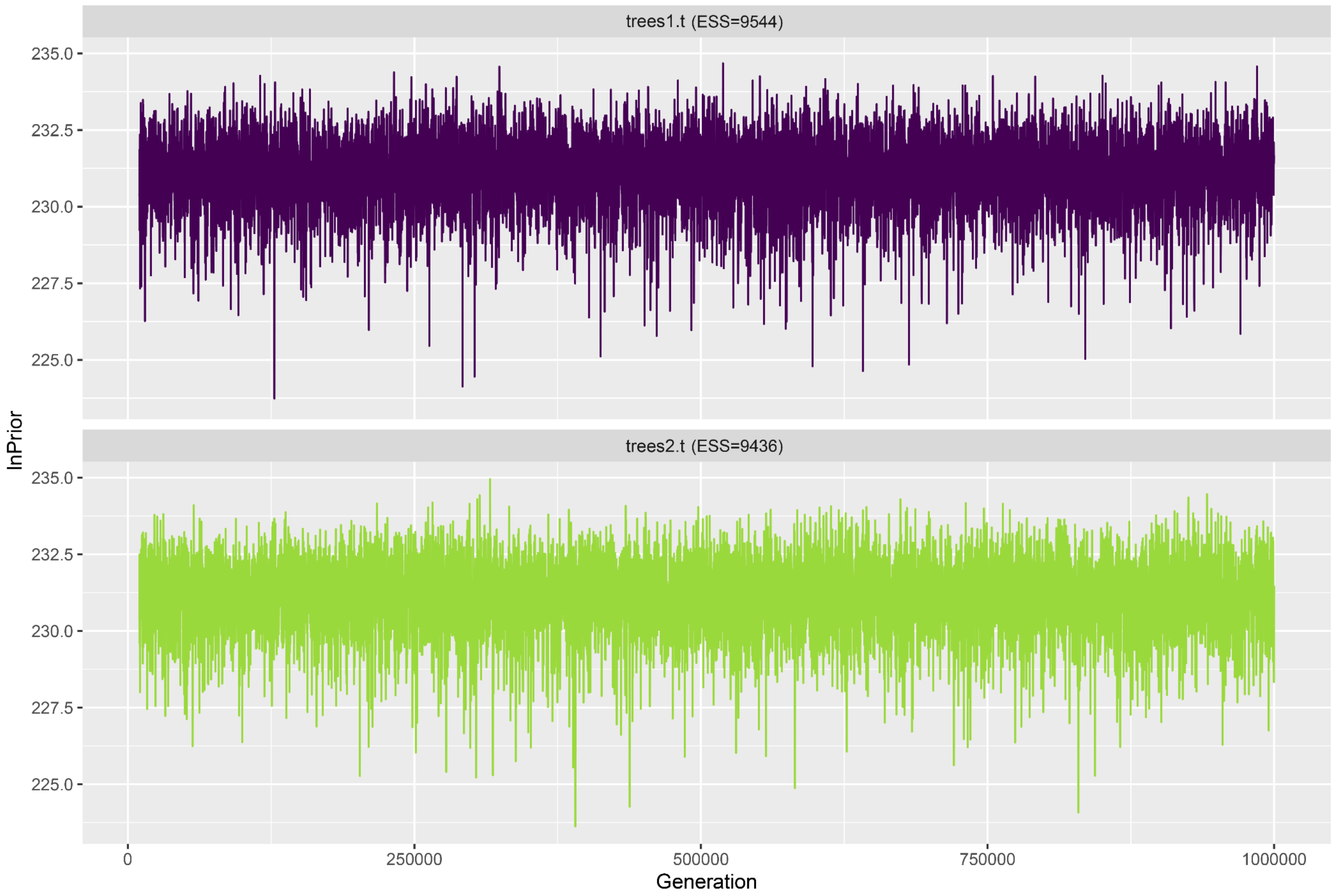

Figure S47. Trace plots of log prior parameters (lnPrior) of two runs of the Phycas MCMC analyses of the 18S + 5.8S +28S rRNA gene dataset masked with a cut-off value of 0.93

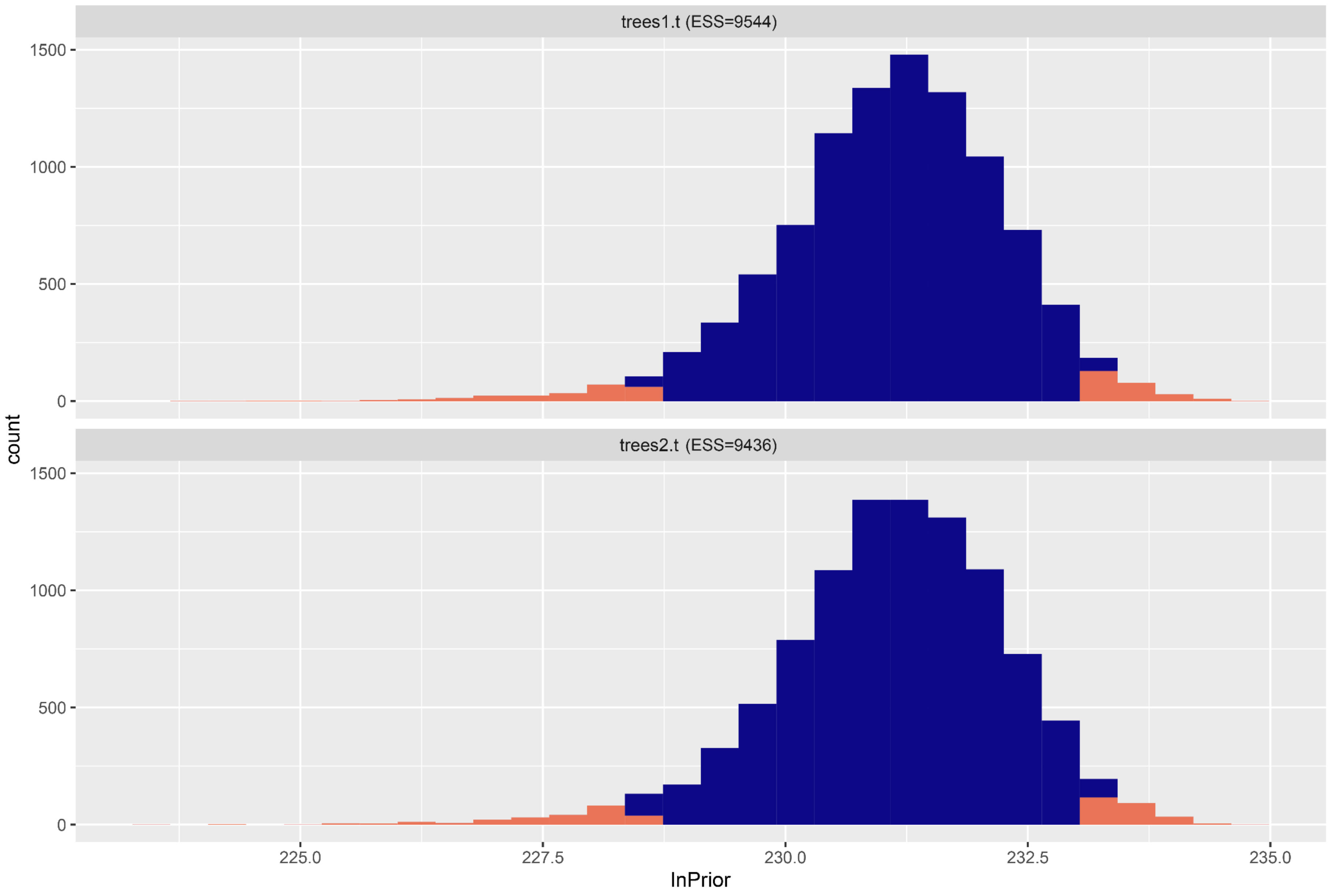

Figure S48. Density plots of log prior parameters (lnPrior) of two runs of the Phycas MCMC analyses of the 18S + 5.8S +28S rRNA gene dataset masked with a cut-off value of 0.93. Red values indicate values outside the 95% credibility intervals

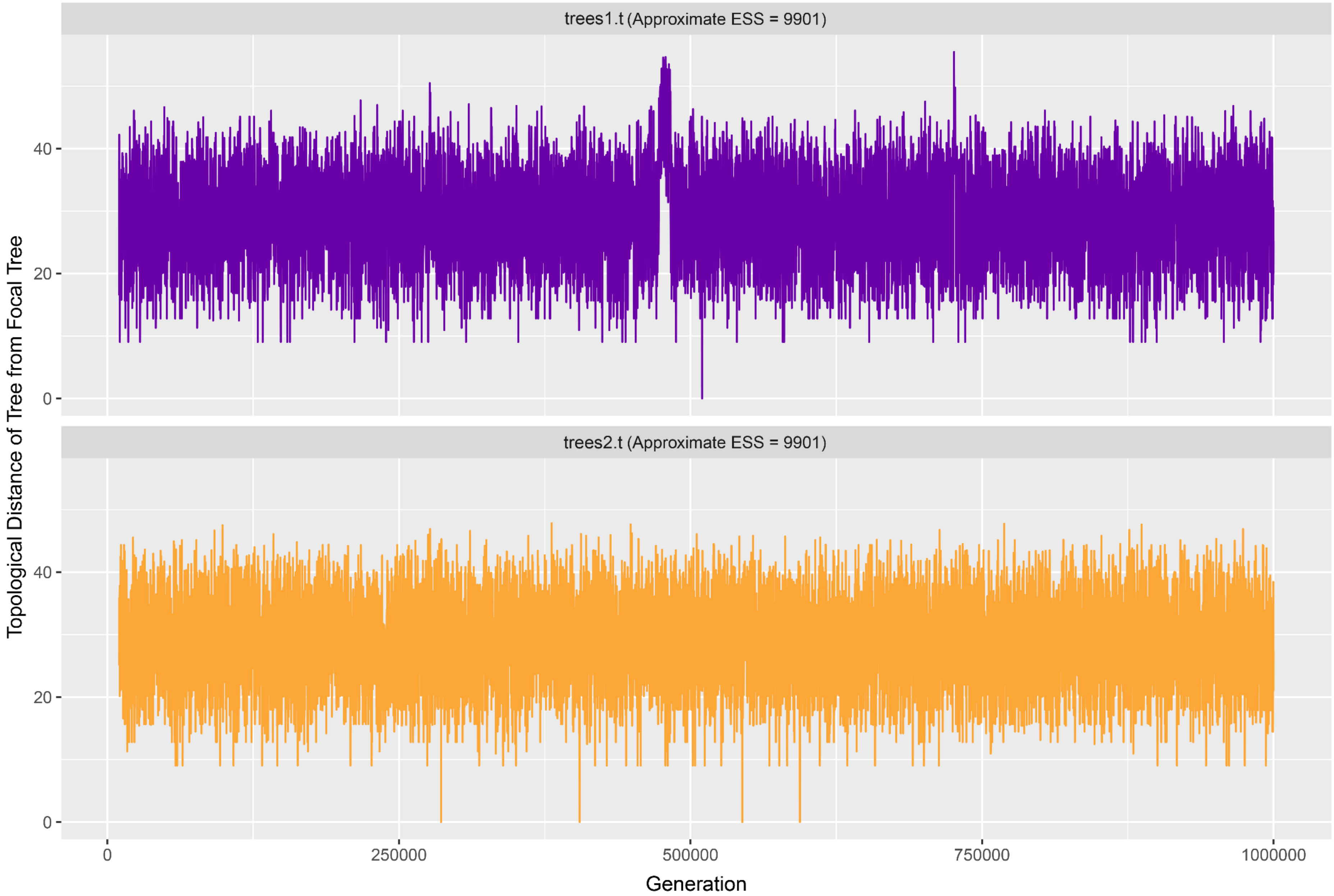

Figure S49. Tree topology trace plots of two runs of the Phycas MCMC analyses of the 18S + 5.8S +28S rRNA gene dataset masked with a cut-off value of 0.93

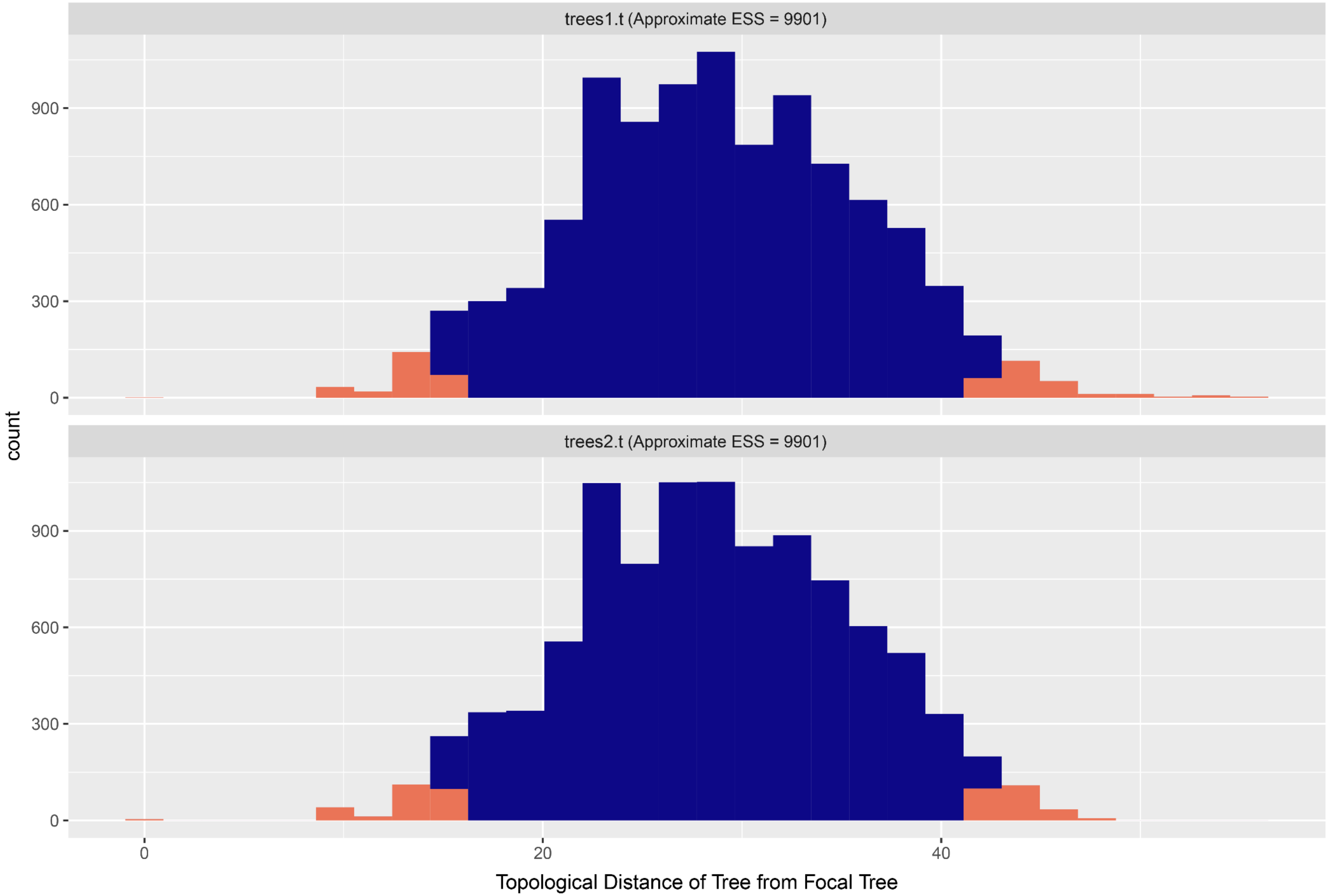

Figure S50. Density plots of tree topology trace of two runs of the Phycas MCMC analyses of the 18S + 5.8S +28S rRNA gene dataset masked with a cut-off value of 0.93. Red values indicate values outside the 95% credibility intervals

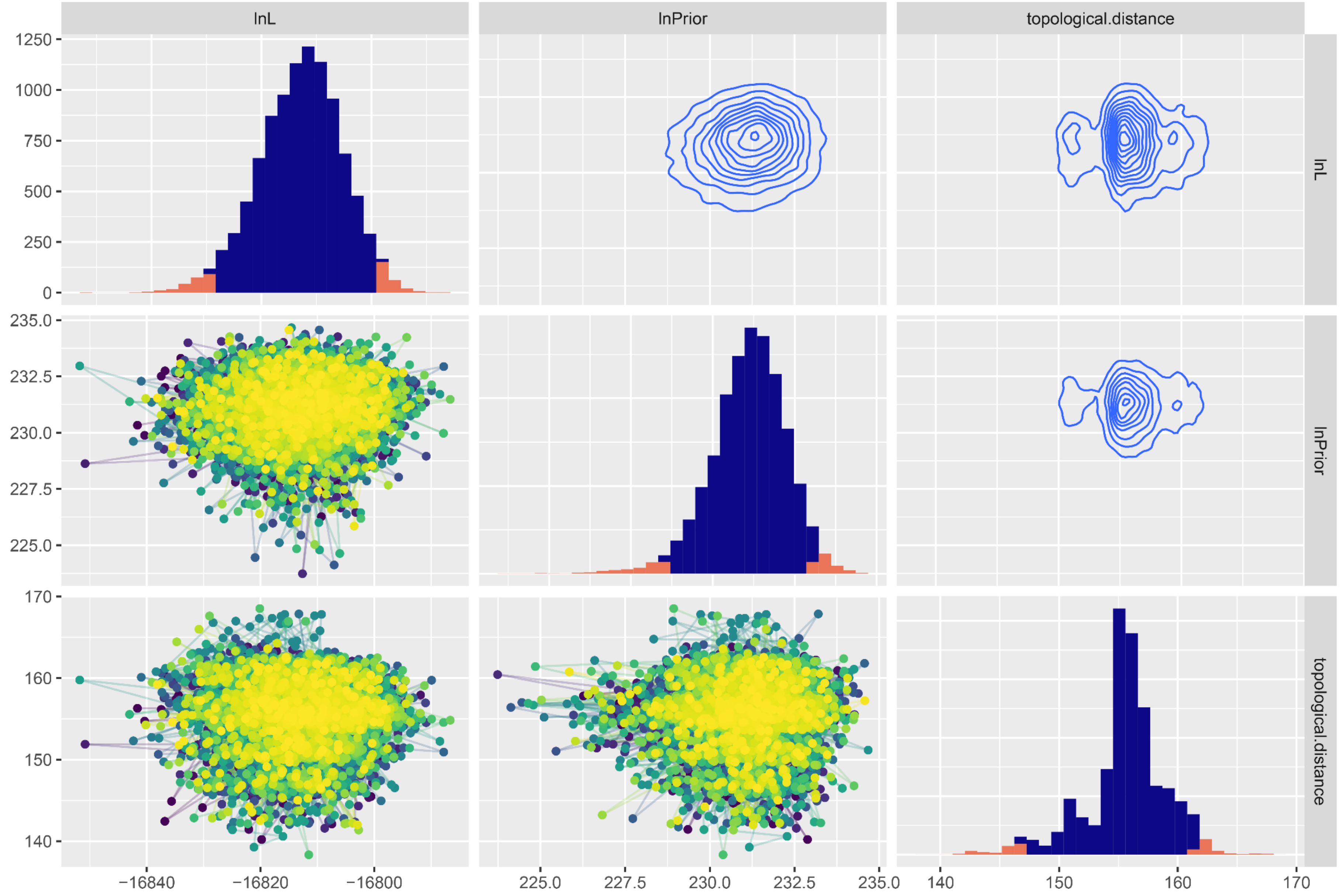

Figure S51. Plots showing correlations between tree topology and continuous model parameters of the first run of the Phycas MCMC analyses of the 18S + 5.8S +28S rRNA gene dataset masked with a cut-off value of 0.93

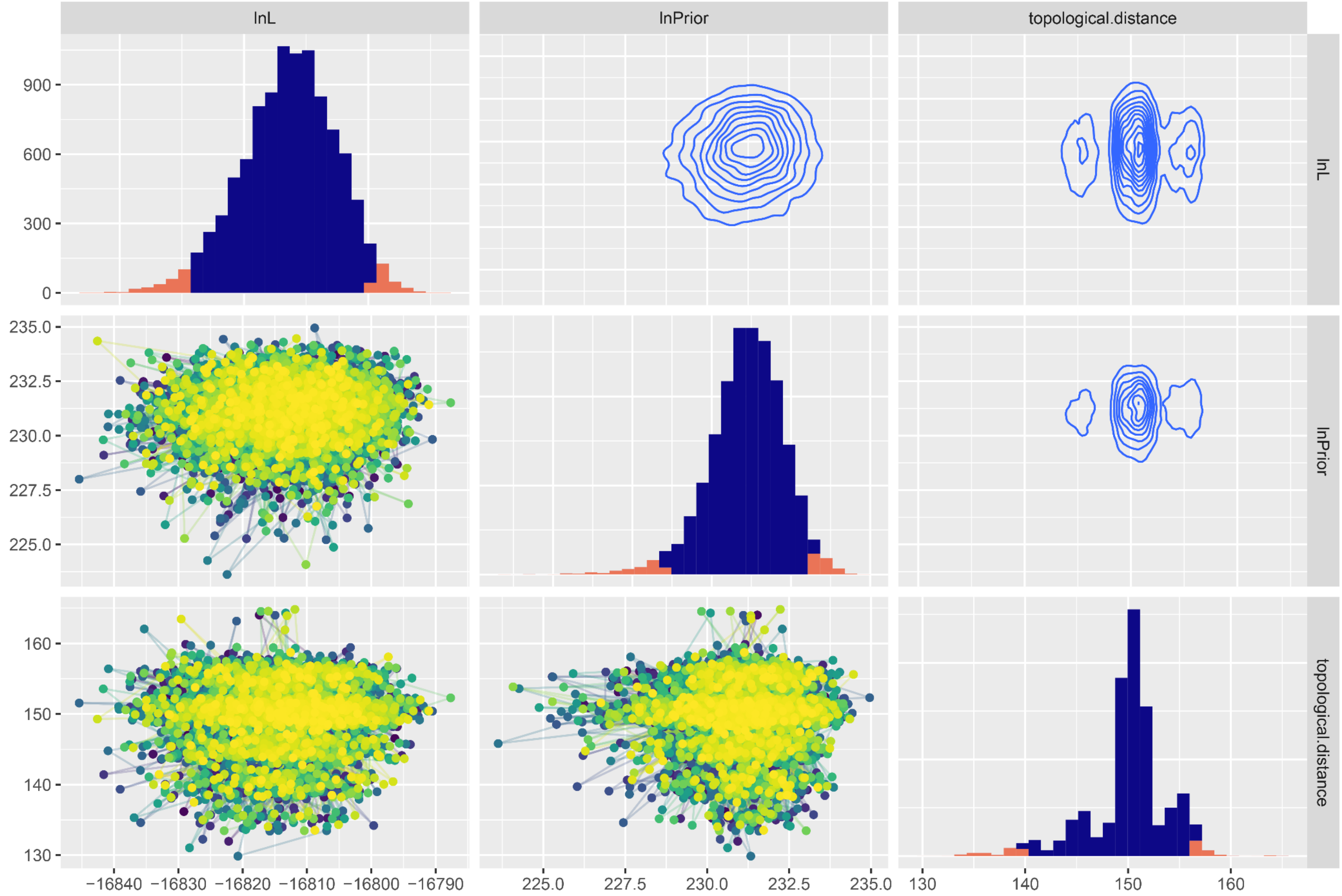

Figure S52. Plots showing correlations between tree topology and continuous model parameters of the second run of the Phycas MCMC analyses of the 18S + 5.8S +28S rRNA gene dataset masked with a cut-off value of 0.93

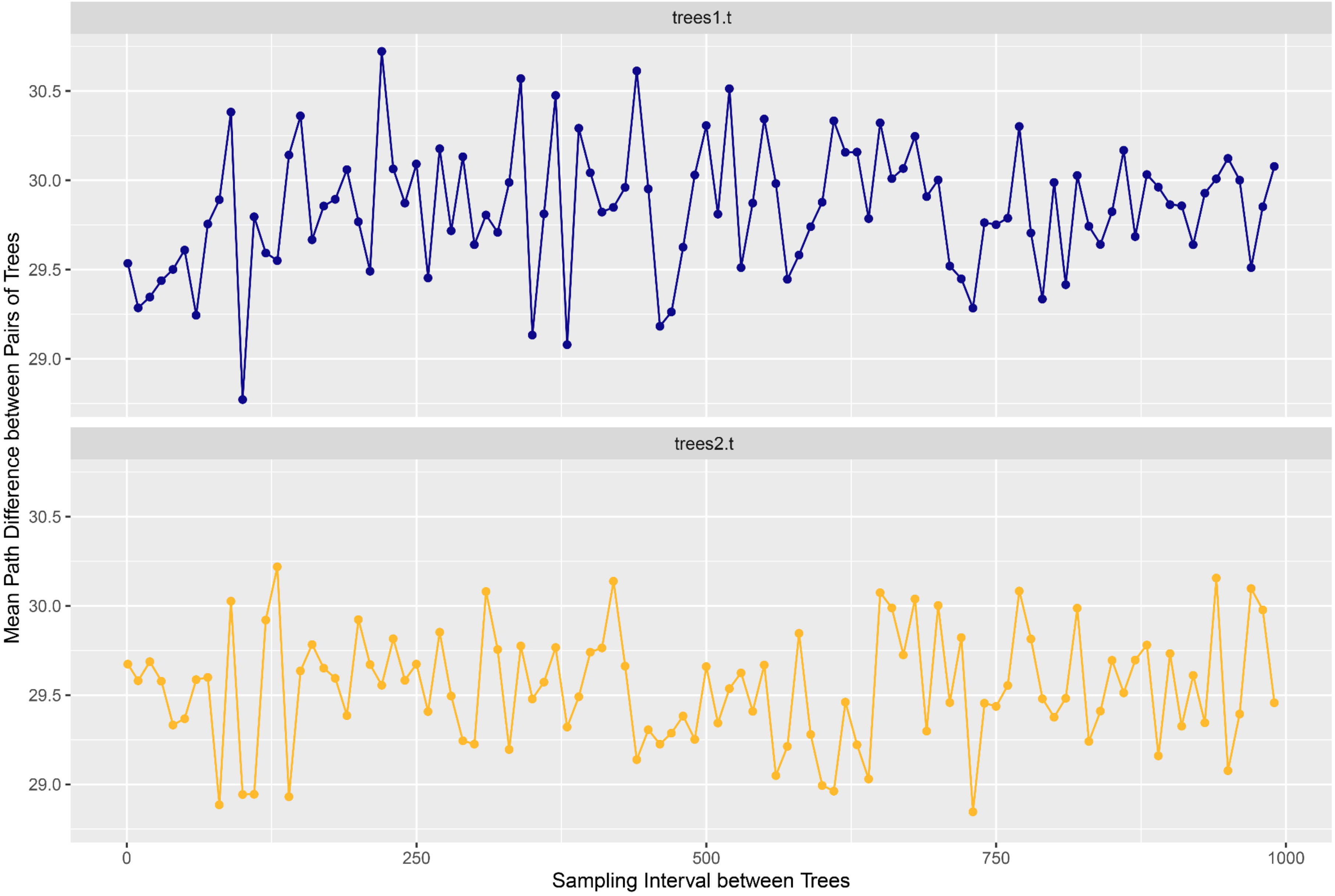

Figure S53. Topological autocorrelation plots of two runs of the Phycas MCMC analyses of the 18S + 5.8S +28S rRNA gene dataset masked with a cut-off value of 0.93

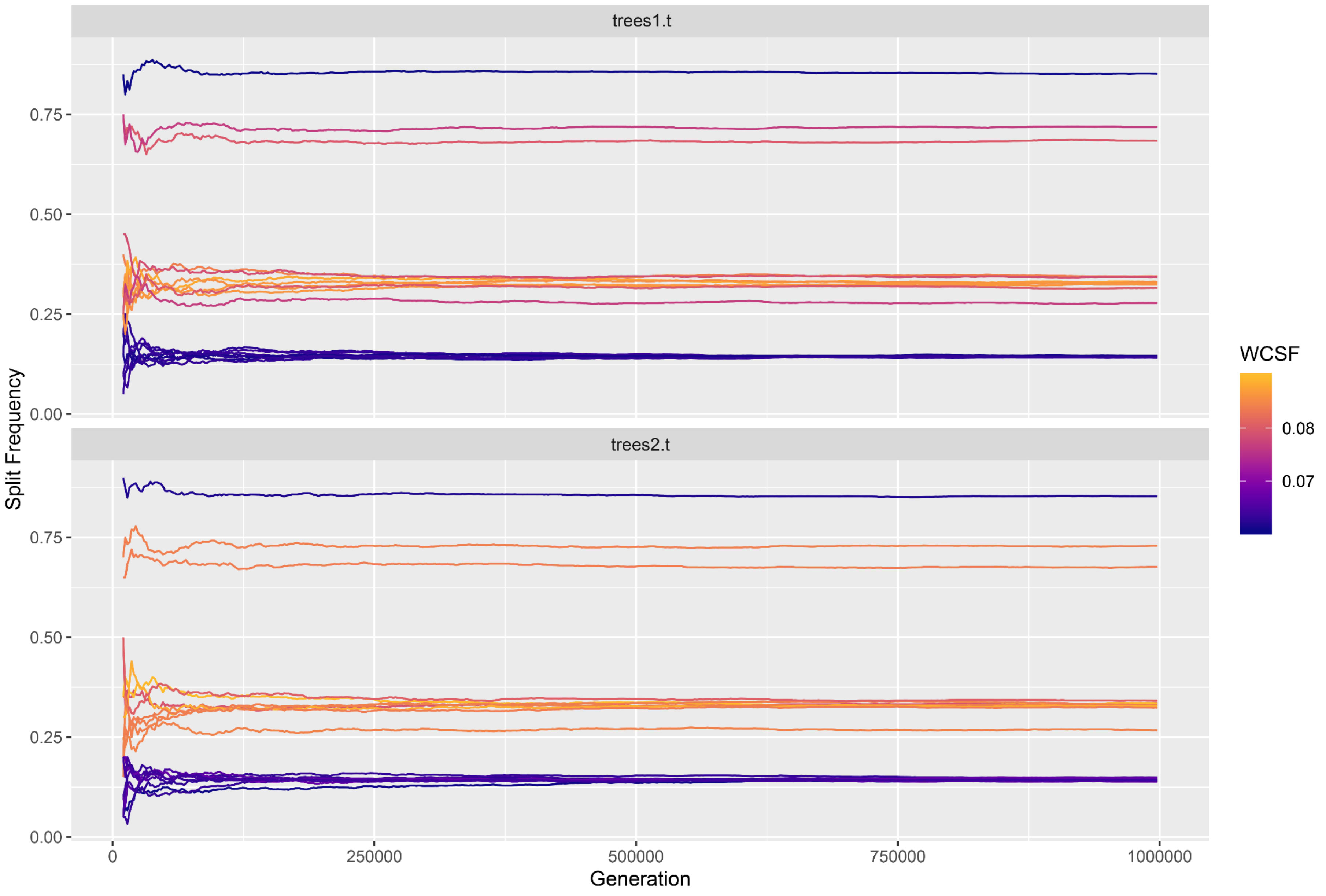

Figure S54. Plots showing cumulative split frequencies for 20 most variable clades of two runs of the Phycas MCMC analyses of the 18S + 5.8S +28S rRNA gene dataset masked with a cut-off value of 0.93

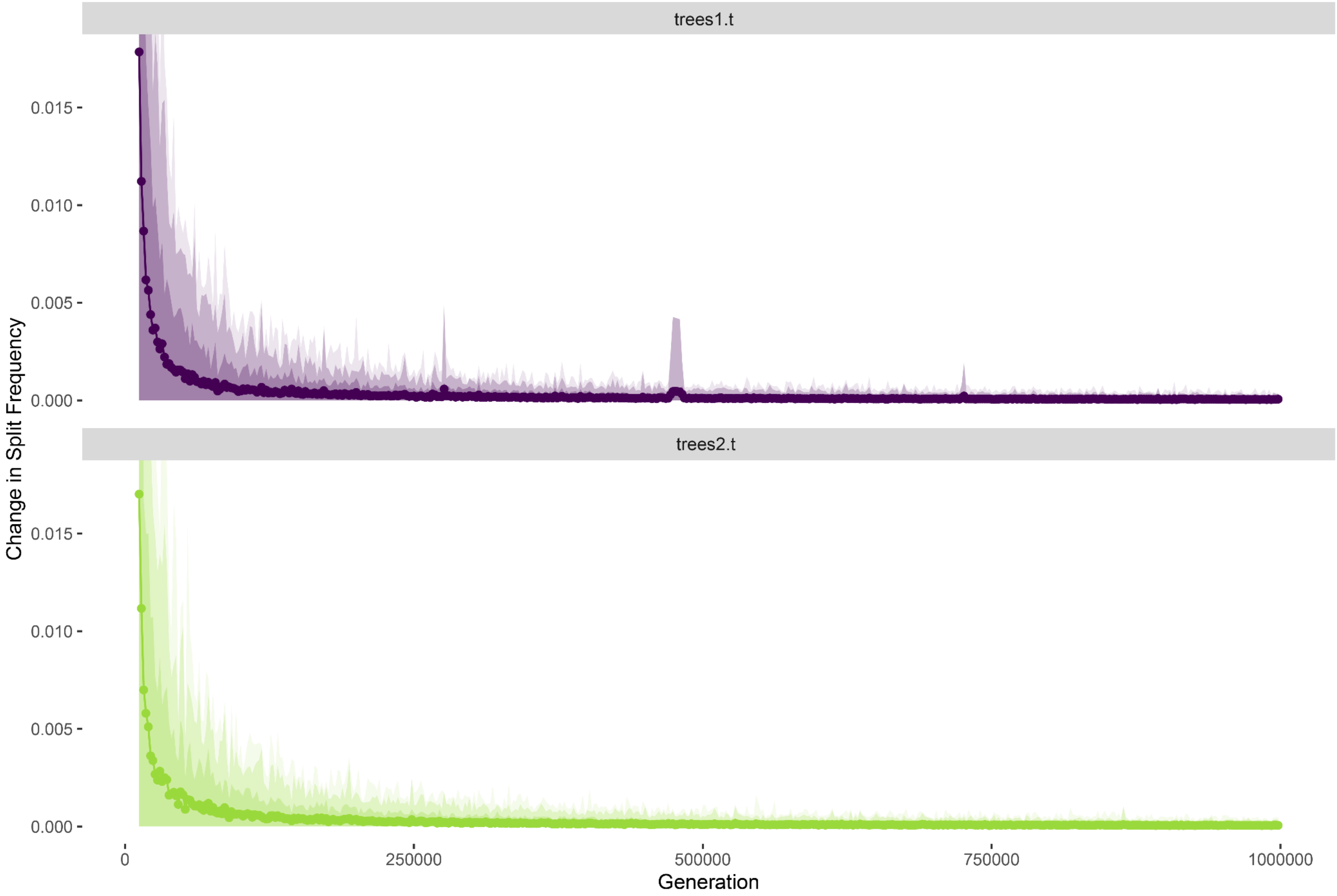

Figure S55. Plots showing cumulative change in split frequencies of two runs of the Phycas MCMC analyses of the 18S + 5.8S +28S rRNA gene dataset masked with a cut-off value of 0.93

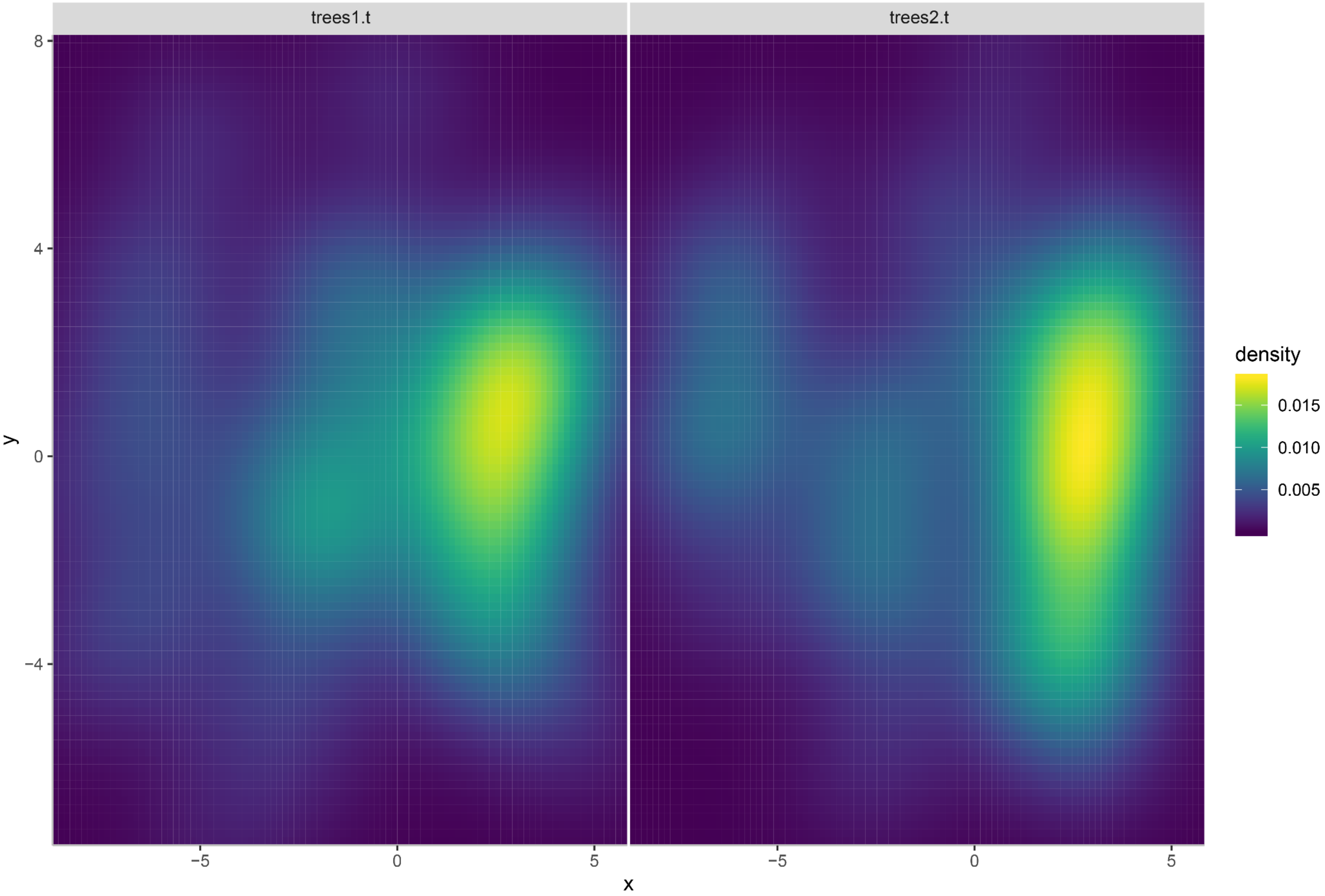

Figure S56. Tree space heatmaps for 100 trees of two runs of the Phycas MCMC analyses of the 18S + 5.8S +28S rRNA gene dataset masked with a cut-off value of 0.93

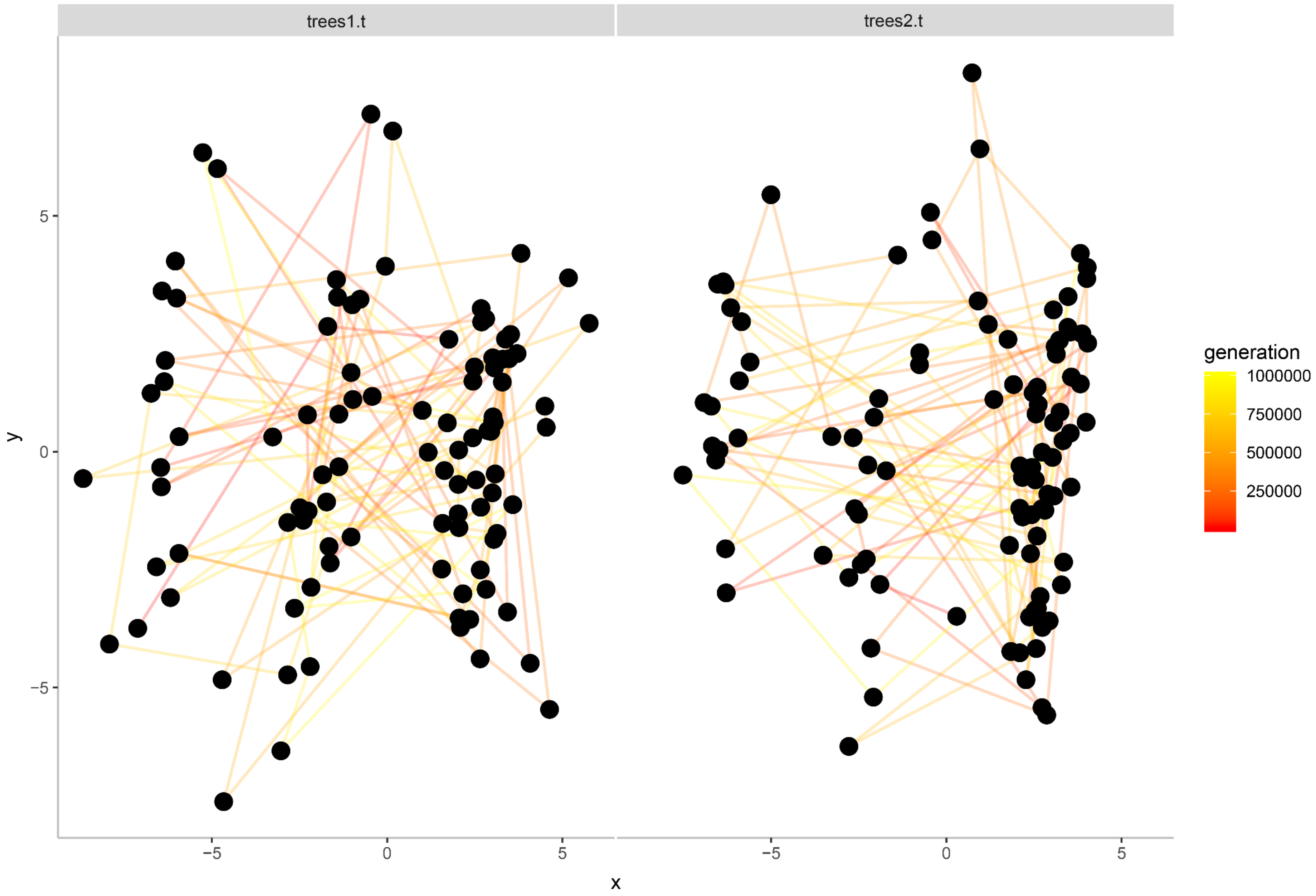

Figure S57. NMDS plots for 100 trees of two runs of the Phycas MCMC analyses of the 18S + 5.8S +28S rRNA gene dataset masked with a cut-off value of 0.93
